# Impaired RelA signaling and lipid metabolism dysregulation in hepatocytes: driving forces in the progression of metabolic dysfunction-associated steatotic liver disease

**DOI:** 10.1038/s41420-025-02312-3

**Published:** 2025-02-05

**Authors:** Yihuai He, Jinlian Jiang, Lili Ou, Yunfen Chen, Aikedaimu Abudukeremu, Guimei Chen, Weiwei Zhong, Zhigang Jiang, Nuerbiye Nuermaimaiti, Yaqun Guan

**Affiliations:** 1 State Key Laboratory of Pathogenesis, Prevention and Treatment of High Incidence Diseases in Central Asia, Xinjiang Key Laboratory of Molecular Biology for Endemic Diseases, Department of Pathology, School of Basic Medical Sciences, Xinjiang Medical University, Urumqi, Xinjiang China; 2 Department of Infectious Diseases, Affiliated Hospital of Zunyi Medical University, Zunyi, Guizhou China; 3 Department of Infectious Diseases, Jingmen Central Hospital, Jingmen, Hubei China; 4 School of Public Health, Zunyi Medical University, Zunyi, Guizhou China; 5 Xinjiang Second Medical College, Karamay, Xinjiang China

**Keywords:** Mechanisms of disease, Non-alcoholic fatty liver disease

## Abstract

RelA, also known as nuclear factor kappa B p65, plays a crucial role in the pathogenesis of various liver diseases. However, the specific role of RelA in hepatocytes during the progression of metabolic dysfunction-associated steatotic liver disease (MASLD) is not well understood. This study explored the relationship between impaired RelA signaling and lipid metabolism disorders in hepatocytes, and how they synergistically contribute to the advancement of MASLD. We assessed the changes, regulatory relationships, and impacts of RelA signaling and lipid metabolism remodeling on disease progression both in vitro and in vivo. During MASLD, there was a decrease in the expression of RelA and hepatocyte nuclear factor 1 alpha (HNF1α), with both factors showing mutual enhancement of each other’s expression under normal conditions. This synergistic effect was absent during hepatocyte steatosis. RelA or HNF1α depletion in hepatocytes intensified MASLD symptoms, whereas overexpression of *RELA* or treatment with necrostatin-1 (a necroptosis inhibitor) or Z-VAD (a caspase inhibitor) significantly mitigated these effects. Mechanistically, during hepatic steatosis, altered lipid profiles exhibited lipotoxicity, inducing hepatocyte apoptosis and necroptosis, whereas endoplasmic reticulum (ER) stress triggered lipid remodeling processes similar to those observed in MASLD. RelA signaling upregulated the expression of activating transcription factor 4 and glucose-regulated protein 78, thereby alleviating ER stress. Impaired RelA signaling remodeled the ER stress response and lipid metabolism, and enhanced lipid accumulation and lipid toxicity. In conclusion, impaired RelA signaling and disrupted lipid metabolism form a detrimental feedback loop in hepatocytes that promotes MASLD progression. Lipid accumulation suppresses RelA signaling, remodeling the ER stress response and exacerbating lipid metabolism disorder, ultimately leading to hepatocyte apoptosis and necroptosis.

## Introduction

Metabolic dysfunction-associated steatotic liver disease (MASLD), formerly known as non-alcoholic fatty liver disease (NAFLD) or metabolic-associated fatty liver disease (MAFLD), is characterized by excessive hepatic lipid accumulation caused by metabolic dysfunction [1–3]. It progresses from simple fatty liver to steatohepatitis, liver fibrosis, and cirrhosis [4, 5]. Cirrhosis significantly increases both the mortality and the incidence of hepatocellular carcinoma in patients with MASLD [6, 7], but effective treatments are lacking [8], highlighting the need for research into disease progression mechanisms and intervention targets.

Lipid metabolism disorder plays a pivotal role in the progression of MASLD. When the liver’s capacity for lipid uptake and synthesis exceeds its ability to break down and excrete these lipids, accumulation within hepatocytes leads to hepatic steatosis [9]. This metabolism disorders can induce endoplasmic reticulum (ER) stress and mitochondrial oxidative stress (MOS), culminating in hepatocyte death via apoptosis and necroptosis [10, 11]. Apoptosis is characterized by the activation of caspase and the formation of apoptotic bodies. Necroptosis is often mediated by inflammatory signals and is characterized by cell membrane lysis, exacerbating inflammatory damage [12]. Mixed lineage kinase domain-like pseudokinase (MLKL) plays a crucial role in necroptosis, with phosphorylation of MLKL (p-MLKL) serving as a classical marker [13]. Ceramide (Cer) and sphingolipids (SPH) are known to increase lysosomal membrane permeability, intensifying necroptosis [14, 15]. Additionally, abnormal lipid metabolism may disrupt the oxidation/reduction balance, further increasing lysosomal membrane permeability, intensifying the inflammatory response, and disrupting mitochondrial function, all of which contribute to necroptosis [16–19]. These insights suggest that lipid metabolism remodeling may trigger hepatocyte necroptosis. Therefore, investigating the mechanisms that drive lipid metabolism disorders holds promise for identifying therapeutic targets for MASLD.

ER stress is closely associated with lipid metabolism disorders. It is caused by ER dysfunction, activates the unfolded protein response (UPR) to restore ER balance [20]. UPR involves pathways like inositol requiring enzyme 1 (IRE1), activating transcription factor 6 (ATF6), and protein kinase R-like ER kinase (PERK)/eukaryotic translational initiation factor 2 alpha (eIF2α)/activating transcription factor 4 (ATF4) pathways [21]. Normally, glucose-regulated protein 78 (GRP78) keeps UPR sensors inactive, but during ER stress, it binds unfolded or misfolded proteins, activating UPR sensors to enhance protein folding and degradation [22]. If these effects of UPR cannot rebuild the homeostasis of the ER, they will activate signaling pathways such as CCAAT-enhancer-binding protein homologous protein homologous protein (CHOP) and caspase-12, mediating cell apoptosis [23–25]. Notably, most studies suggest that severe and prolonged ER stress can also increase lipid synthesis by activating signaling such as sterol regulatory element binding protein 1 (SREBP1), promoting hepatic steatosis [26–28]. In addition, ER stress can damage the electron transport chain (ETC) of mitochondria, causing an increase in reactive oxygen species (ROS) and promoting lipid peroxidation [29, 30]. Therefore, although the activation of some UPR-related signaling promotes ER homeostasis, the sustained ER stress states will trigger the progression of MASLD, which may be related to reshaping lipid metabolism.

RelA, also known as nuclear factor kappa B p65 (NF-κB p65), is a vital member of the NF-κB family [31]. Evidence has shown that RelA can regulate the transcriptional activity of hundreds of genes and play regulatory roles in various biological processes, including energy homeostasis and metabolisms [32]. Its effects may differ depending on cellular location. RelA is involved in liver injury, fibrosis, and cirrhosis through its ability to regulate the function of different liver cells [33, 34]. Additionally, RelA participates in liver regeneration and cancer development by influencing hepatocyte proliferation and death [35]. RelA is a protective factor in hepatocytes, playing a critical protective role in apoptosis [36]. Preliminary research indicates that RelA was upregulated under ER stress in hepatocytes; the upregulated RelA increases the expression of hepatocyte nuclear factor 1 alpha (HNF1α) and UPR-related proteins (such as ATF4, GRP78) to provide feedback and alleviate ER stress in liver injury [37]. These findings suggest that RelA may affect the homeostasis of lipid metabolism by modulating the response of hepatocytes to ER stress.

HNF1α, which is abundant in hepatocytes, plays a critical role in regulating cellular metabolism and inflammation by controlling genes associated with detoxification, glucose and lipid metabolism, bile acid metabolism, and the synthesis of plasma proteins [38, 39]. Recent research suggests that HNF1α promotes fatty acid oxidation, enhances glucose utilization, and improves lipid metabolism and insulin sensitivity [40]. However, its expression is diminished in steatotic hepatocytes [41], which is known to activate RelA transcription [42]. Therefore, there is a potential reciprocal enhancement of transcriptional activity between HNF1α and RelA in hepatocytes. However, when excessive lipid accumulation occurs within hepatocytes, this mutual promotion may be inhibited, leading to impairment of the RelA signaling and consequently exacerbating lipid metabolic disorders. This study aims to investigate how hepatocyte RelA signaling affects the progression of MASLD.

## Results

### Steatotic hepatocytes exhibit decreased levels of RelA and HNF1α in MASLD

**Fig. 1:**
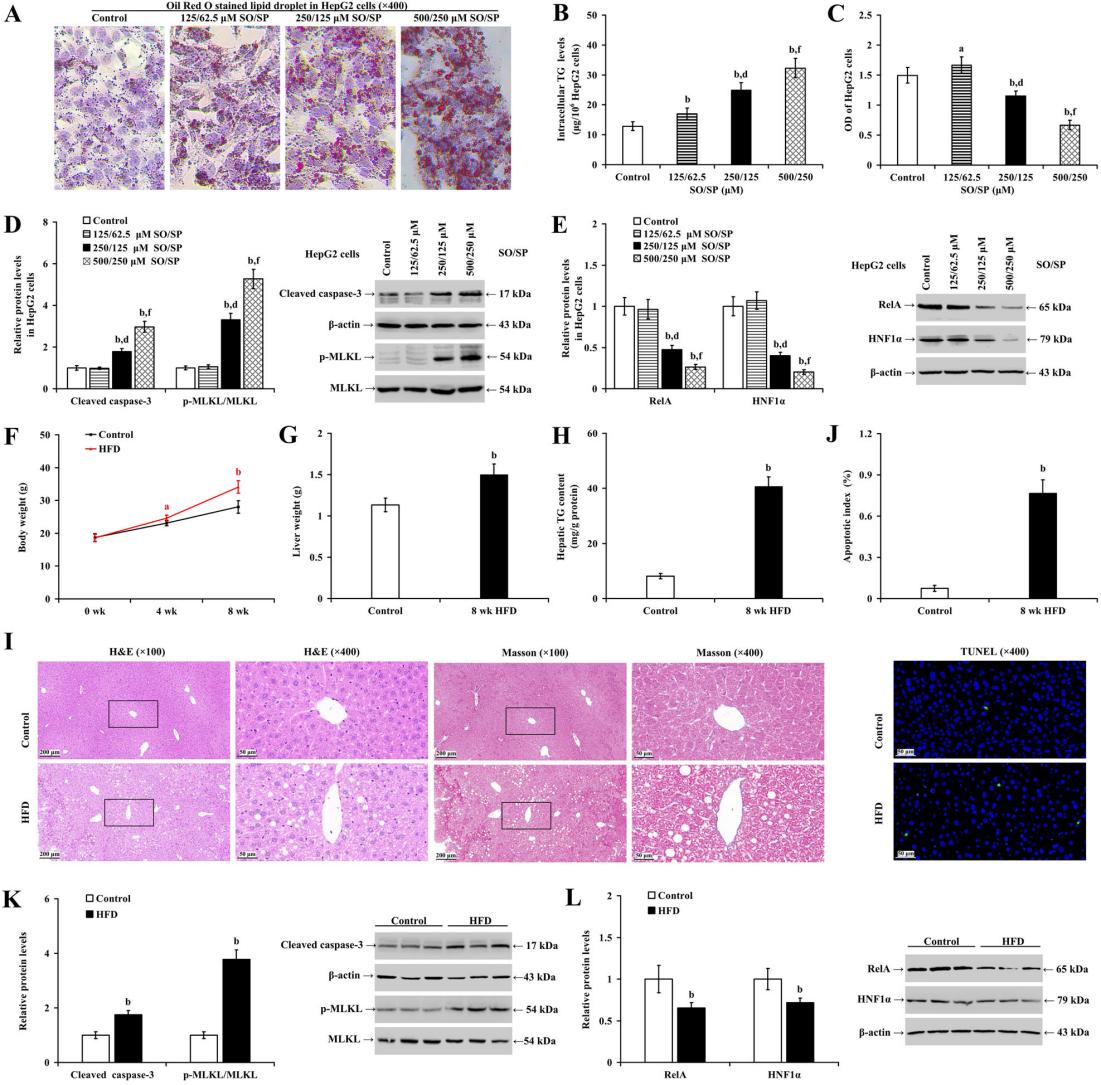
Steatotic hepatocytes exhibit reduced levels of RelA and HNF1α in MASLD. **A**–**E** HepG2 cells were treated with SO/SP or vehicle at the indicated concentrations to induce steatosis. Under this condition, **A** lipid droplet accumulation, **B** intracellular TG levels, **C** cell viability and the expression of **D** cleaved caspase-3, p-MLKL, and **E** RelA and HNF1α were evaluated. Comparisons of **F** body weight, **G** liver weight, and **H** hepatic TG content were evaluated and analyzed in mice (n = 12) fed a normal diet or HFD. **I** Representative images of H&E (left panels) and Masson staining (right panels) of mouse livers from the indicated groups. **J** Hepatic apoptotic index (upper panel) and representative images of TUNEL staining (lower panels). Western blot analysis and quantification showing the expression of **K** cleaved caspase-3, p-MLKL, and **L** RelA and HNF1α in mouse livers under different conditions. a *P* < 0.05, b *P* < 0.01, vs. control group; d *P* < 0.01, vs. SO/SP group (125/62.5 μM); f *P* < 0.01, vs. SO/SP group (250/125 μM). HFD high-fat diet, SO/SP sodium oleate and sodium palmitate, μM μmol/L, wk week.

To establish in vitro steatosis, different concentrations of SO/SP (125/62.5, 250/125, and 500/250 μM) were used to induce hepatocyte lipid degeneration over 48 h. In HepG2 (Fig. 1A–E) and LO2 cells (Supplementary Fig. 1A–E), SO/SP treatment concentration-dependently caused lipid accumulation (Fig. 1A and Supplementary Fig. 1A) and increased intracellular TG levels (Fig. 1B and Supplementary Fig. 1B; *P* < 0.01). Cell viability was increased at 125/62.5 μM SO/SP (*P* < 0.05), but it was significantly suppressed at 250/125 and 500/250 μM SO/SP (Fig. 1C and Supplementary Fig. 1C; *P* < 0.01). These observations indicate that the 250/125 and 500/250 μM SO/SP successfully induced hepatocyte steatosis. We observed that SO/SP significantly promoted apoptosis and necroptosis, as reflected by increased cleaved caspase-3 and p-MLKL, respectively (Fig. 1D and Supplementary Fig. 1D; *P* < 0.01). Meanwhile, SO/SP downregulated RelA and HNF1α (Fig. 1E and Supplementary Fig. 1E; *P* < 0.01). Notably, at a concentration of 500/250 μM SO/SP, the viability inhibition rate of hepatocytes exceeded 50%, indicating significant cytotoxicity. To ensure the validity of the experiments and to avoid compromising the accuracy of the results due to excessive toxicity, we adopted 250/125 μM SO/SP for subsequent experiments.

To establish the MASLD model, mice were fed an HFD for eight weeks. Compared to the control group, the HFD group showed a significant increase in body weight and liver weight (Fig. 1F and G; *P* < 0.01), as well as serum ALT (Supplementary Fig. 1F; *P* < 0.01), and TC levels (Supplementary Fig. 1G; *P* < 0.01). However, there were significant decrease in serum TG levels (Supplementary Fig. 1H; *P* < 0.05). Mouse liver TG content increased in the HFD group (Fig. 1H; *P* < 0.01). H&E, Masson staining, and TUNEL assay revealed significant lipid degeneration and apoptosis (*P* < 0.01), but no significant fibrosis or inflammatory cell infiltration was observed (Fig. 1I and J). Western blot analyses showed that HFD elevated the cleavage of caspase-3 and p-MLKL (Fig. 1K; *P* < 0.01) and reduced RelA and HNF1α expression in mouse liver at week 8 (Fig. 1L; *P* < 0.01).

We used an in vitro model of ER stress induced by THA as the positive control. The results showed that THA significantly inhibited cell viability (Supplementary Fig. 2A; *P* < 0.01) and upregulated the expression of RelA, HNF1α, and GRP78 (indicative of ER stress activation) in HepG2 cells (Supplementary Fig. 2B; *P* < 0.01).

To determine the expression of RelA and HNF1α in the positive control livers under ER stress in vivo, mice were treated with TM or vehicle (DMSO) for eight weeks. TM resulted in a significant body weight loss (Supplementary Fig. 2C; *P* < 0.01) and a reduction in serum TC levels (*P* < 0.01) in mice. However, it elevated their liver weight (*P* < 0.01), serum ALT levels (*P* < 0.01), and TG levels (Supplementary Fig. 2D–G; *P* < 0.01). Some hepatocytes underwent microvascular steatosis, with less than 5% of hepatocytes showing lipid degeneration. Significant pathological changes were observed in the liver, including hepatocyte necrosis, fibrosis, infiltration of inflammatory cells, and apoptosis (Supplementary Fig. 2H and I; *P* < 0.01). Western blot analysis revealed that the levels of RelA, HNF1α, and GRP78 were significantly increased in the liver (Supplementary Fig. 2J; *P* < 0.01).

### Lack of reciprocal transcriptional activation between RelA and HNF1α in hepatocyte steatosis

**Fig. 2:**
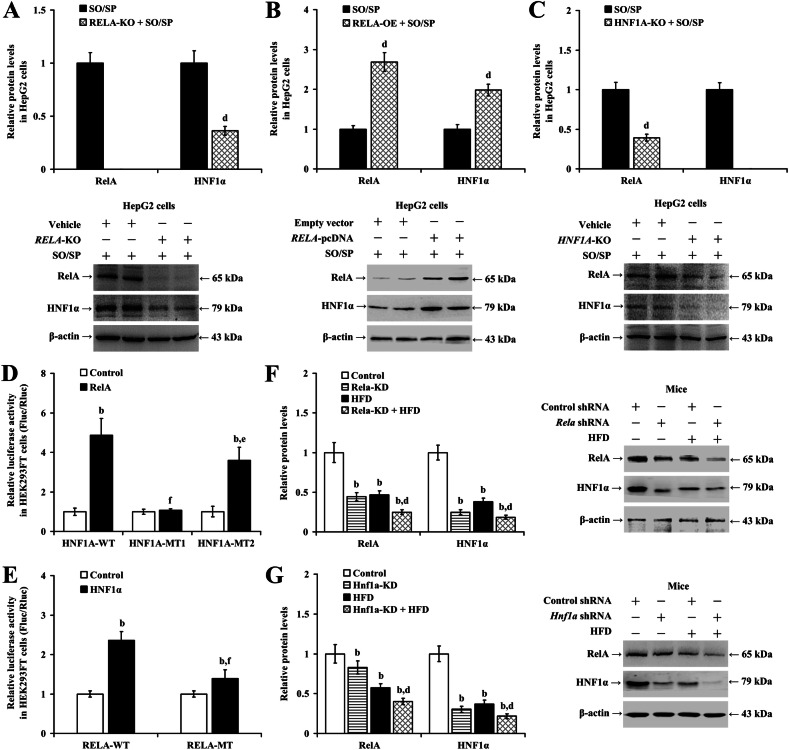
Lack of mutual transcriptional activation between RelA and HNF1α during hepatocyte steatosis. **A**–**C** Quantification (upper panels) and representative images of western blot analysis (lower panels) showing the expression of RelA and HNF1α in HepG2 cells under the indicated conditions. Results of luciferase reporter assay evaluating the **D** bindings between RelA and *HNF1A* promoter and **E** the bindings between HNF1α and *RELA* promoter. Analyzed data (left panels) and representative images (right panels) of Western blot analysis showing the expression of RelA and HNF1α in mouse livers with **F** *Rela*-KD and **G** *Hnf1a*-KD. b *P* < 0.01, vs. control group; d *P* < 0.01, vs. SO/SP group, or HFD group; e *P* < 0.05, f *P* < 0.01, vs. *HNF1A*-WT group, or *RELA*-WT group. HFD high-fat diet, KD knockdown, KO knockout, MT mutated, OE overexpression, SO/SP sodium oleate and sodium palmitate, WT wild type.

Our experiments using HepG2 and LO2 cells noted significant findings related to RelA and HNF1α interactions following specific genetic manipulations and treatments. In both HepG2 (Fig. 2A; *P* < 0.01) and LO2 (Supplementary Fig. 3A; *P* < 0.01) cells, the reduction of HNF1α induced by SO/SP treatment was exacerbated by either knocking out or silencing the *RELA* gene. Conversely, overexpression of *RELA* significantly increased HNF1α expression in steatotic cells in vitro (Fig. 2B and Supplementary Fig. 3B; *P* < 0.01). Additionally, the SO/SP-induced reduction in RelA was intensified by *HNF1A* knockout in HepG2 cells (Fig. 2C; *P* < 0.01) and by *HNF1A* knockdown in LO2 cells (Supplementary Fig. 3C; *P* < 0.01).

Based on these observations, we proposed a mutual transcriptional activation between RelA and HNF1α in hepatocytes. To test this hypothesis, a dual luciferase reporter assay was conducted. The assay revealed that, compared to the control group, the interaction of the wild-type *HNF1A* promoter reporter vector (*HNF1A*-WT) with overexpressed *RELA* led to an increase in the Fluc/Rluc value (Fig. 2D; *P* < 0.01). In contrast, interaction with a mutant promoter reporter vector significantly decreased the Fluc/Rluc value compared with that in the *HNF1A*-WT group (*P* < 0.05), indicating that RelA likely binds to the WT *HNF1A* promoter to enhance transcription. Further, co-transfection of the *RELA* promoter reporter vector with an *HNF1A-*overexpressing construct significantly increased the Fluc/Rluc value, while a mutation in the *RELA* promoter resulted in a weakened effect (Fig. 2E; *P* < 0.01), supporting the notion that HNF1α similarly enhances *RELA* transcriptional activity.

In vivo MASLD mice were generated to confirm these findings. We observed that HFD administration reduced the expression of RelA and HNF1α in the mouse liver (Fig. 2F and G; *P* < 0.01). Moreover, the knockdown of *Rela* with shRNA resulted in further reduced protein levels of HNF1α in the liver (Fig. 2F; *P* < 0.01), and vice versa (Fig. 2G; *P* < 0.01).

These findings suggest that HNF1α upregulated RelA, and RelA upregulated HNF1α in hepatocytes. However, this interaction was inhibited during steatosis.

### Hepatocyte RelA mitigates hepatic steatosis

**Fig. 3:**
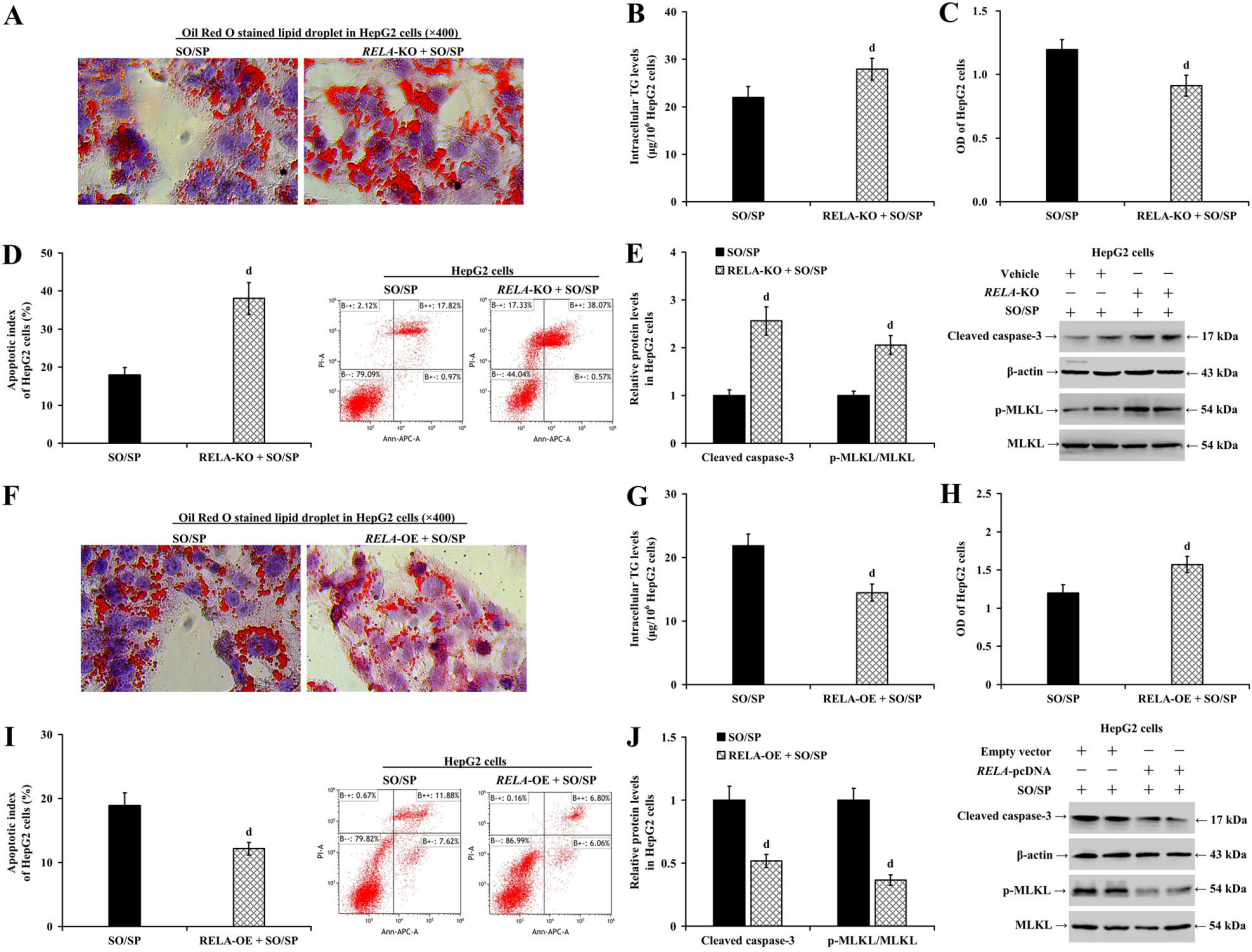
RelA decreases steatosis in SO/SP-induced HepG2 cells. HepG2 cells with or without *RELA*-KO were treated with SO/SP. Under this condition, **A** lipid droplet accumulation, **B** intracellular TG levels, **C** cell viability, **D** cell apoptosis, and **E** the expression of cleaved caspase-3 and p-MLKL were determined and compared. HepG2 cells with or without *RELA*-OE were treated with SO/SP, and **F** lipid droplet accumulation, **G** intracellular TG levels, **H** cell viability, **I** cell apoptosis, and **J** the expression of cleaved caspase-3 and p-MLKL were determined and analyzed. d *P* < 0.01, vs. SO/SP group. KO knockout, OE overexpression, SO/SP sodium oleate and sodium palmitate.

We utilized an in vitro steatosis model with or without RelA knockout in HepG2 cells or RelA silencing in LO2 cells to validate our findings. In both HepG2 (Fig. 3A–E) and LO2 cells (Supplementary Fig. 4A–E), RelA depletion exacerbated SO/SP-induced cell lipid degeneration (Fig. 3A and Supplementary Fig. 4A), increased intracellular TG levels (Fig. 3B and Supplementary Fig. 4B; *P* < 0.01), and reduced cell viability, further exaggerating the SO/SP-mediated reduction in cell viability (Fig. 3C and Supplementary Fig. 4C; *P* < 0.05). Additionally, it significantly increased the apoptotic index (Fig. 3D and Supplementary Fig. 4D; *P* < 0.01) and the protein expression of cleaved caspase-3 and p-MLKL (Fig. 3E and Supplementary Fig. 4E; *P* < 0.01).

To further confirm our findings, we performed gain-of-function assays by overexpressing *RELA* in HepG2 (Fig. 3F–J) and LO2 cells (Supplementary Fig. 4F–J) and treating them with SO/SP. Overexpression of *RELA* reduced cell lipid degeneration (Fig. 3F and Supplementary Fig. 4F) and intracellular TG levels (Fig. 3G and Supplementary Fig. 4G; *P* < 0.01). It also restored the cell viability reduced by SO/SP exposure (Fig. 3H and Supplementary Fig. 4H; *P* < 0.05) and reduced cell apoptosis (Fig. 3I and Supplementary Fig. 4I; *P* < 0.01). In addition, *RELA* overexpression decreased the protein expression of cleaved caspase-3 and p-MLKL (Fig. 3J and Supplementary Fig. 4J; *P* < 0.01).

**Fig. 4:**
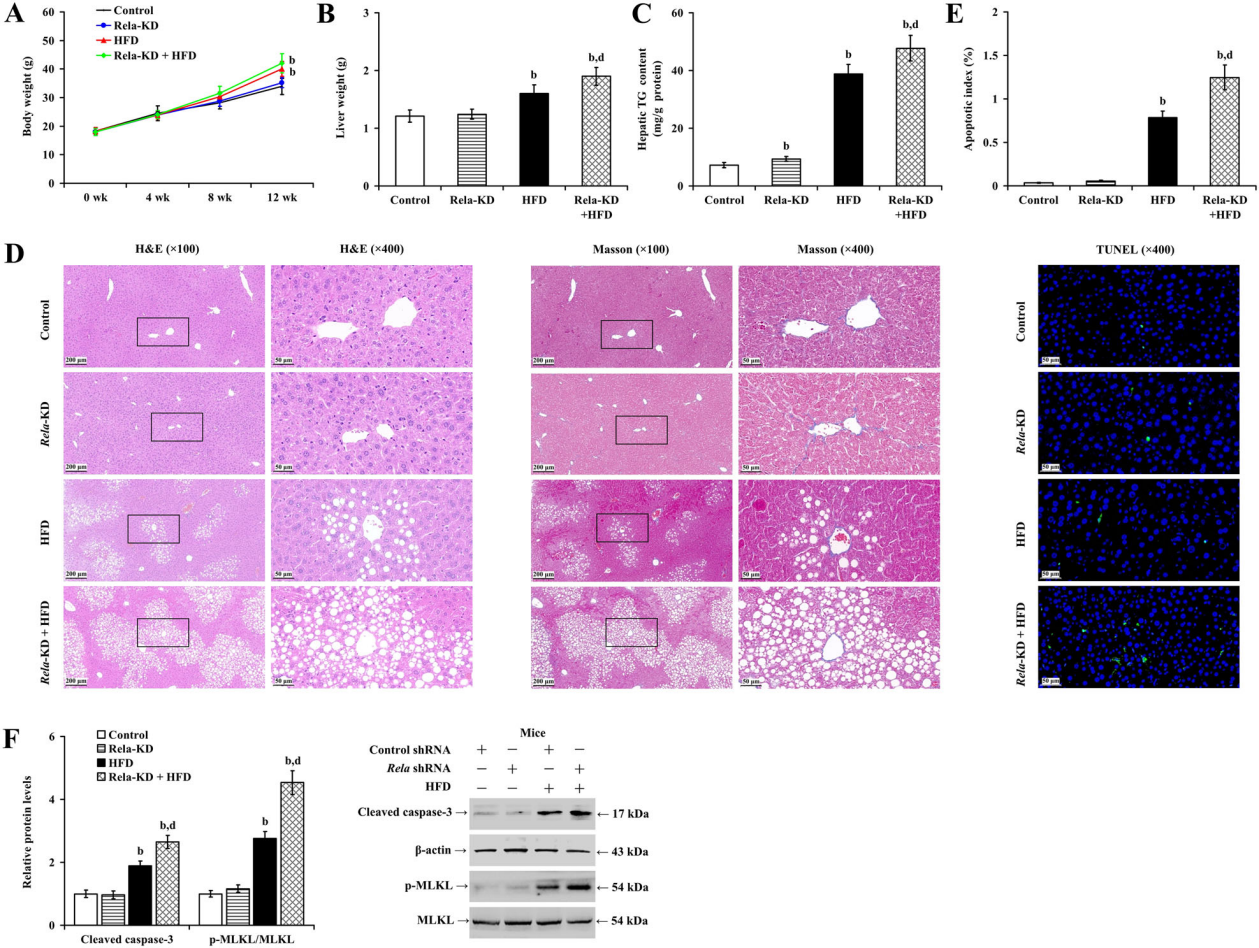
Knockdown of *Rela* exacerbates HFD-induced hepatic steatosis in mice. Analyzed data showing the **A** body weight changes, **B** liver weights, and **C** hepatic TG content of the mice (n = 12) under indicated conditions. **D** Representative images of H&E staining (left panels) and Masson staining (right panels) depicting the pathologic changes of mouse livers under different treatments. **E** The calculated apoptotic index (upper panels) and representative images of TUNEL staining (lower panel). **F** Analyzed data and representative images of Western blot analysis showing the protein expression of cleaved caspase-3 and p-MLKL in mouse livers under different conditions. b *P* < 0.01, vs. control group; d *P* < 0.01, vs. HFD group. HFD high-fat diet, KD knockdown.

To understand the role of hepatocyte RelA expression in MASLD in vivo, we induced hepatic steatosis in mice with or without RelA depletion. Compared with the control group, silencing *Rela* did not change body weight or liver weight (Fig. 4A and B; *P* > 0.05), hepatic TG content (Fig. 4C; *P* > 0.05), or serum ALT, TC, and TG levels (Supplementary Fig. 4K–M; *P* > 0.05) in mice on a normal diet. RelA depletion only resulted in mild hepatocyte steatosis, with less than 5% of degenerative hepatocytes and no detectable inflammatory cell infiltration, fibrotic tissue proliferation, or hepatic apoptosis (Fig. 4D and E; *P* > 0.05). Compared to the HFD group, the knockdown of *Rela* did not alter mouse body weight (Fig. 4A; *P* > 0.05), and serum TG levels (Supplementary Fig. 4M; *P* > 0.01). However, depletion of RelA significantly increased liver weight (Fig. 4B; *P* < 0.01), liver TG content (Fig. 4C; *P* < 0.01), serum ALT (Supplementary Fig. 4K; *P* < 0.01), and TC levels (Supplementary Fig. 4L; *P* < 0.05), thus exacerbating the effects of HFD-induced hepatic steatosis, and apoptosis (Fig. 4D, E; *P* < 0.01). At the molecular level, while transfection with *Rela* shRNA alone did not alter the levels of cleaved caspase-3 or p-MLKL, it significantly enhanced HFD-induced apoptosis and necroptosis, as evidenced by increased levels of cleaved caspase-3 and p-MLKL in the liver (Fig. 4F; *P* < 0.01). These results underscore the role of hepatocyte RelA in mitigating MASLD.

### Hepatocyte HNF1α reduces hepatic steatosis

**Fig. 5:**
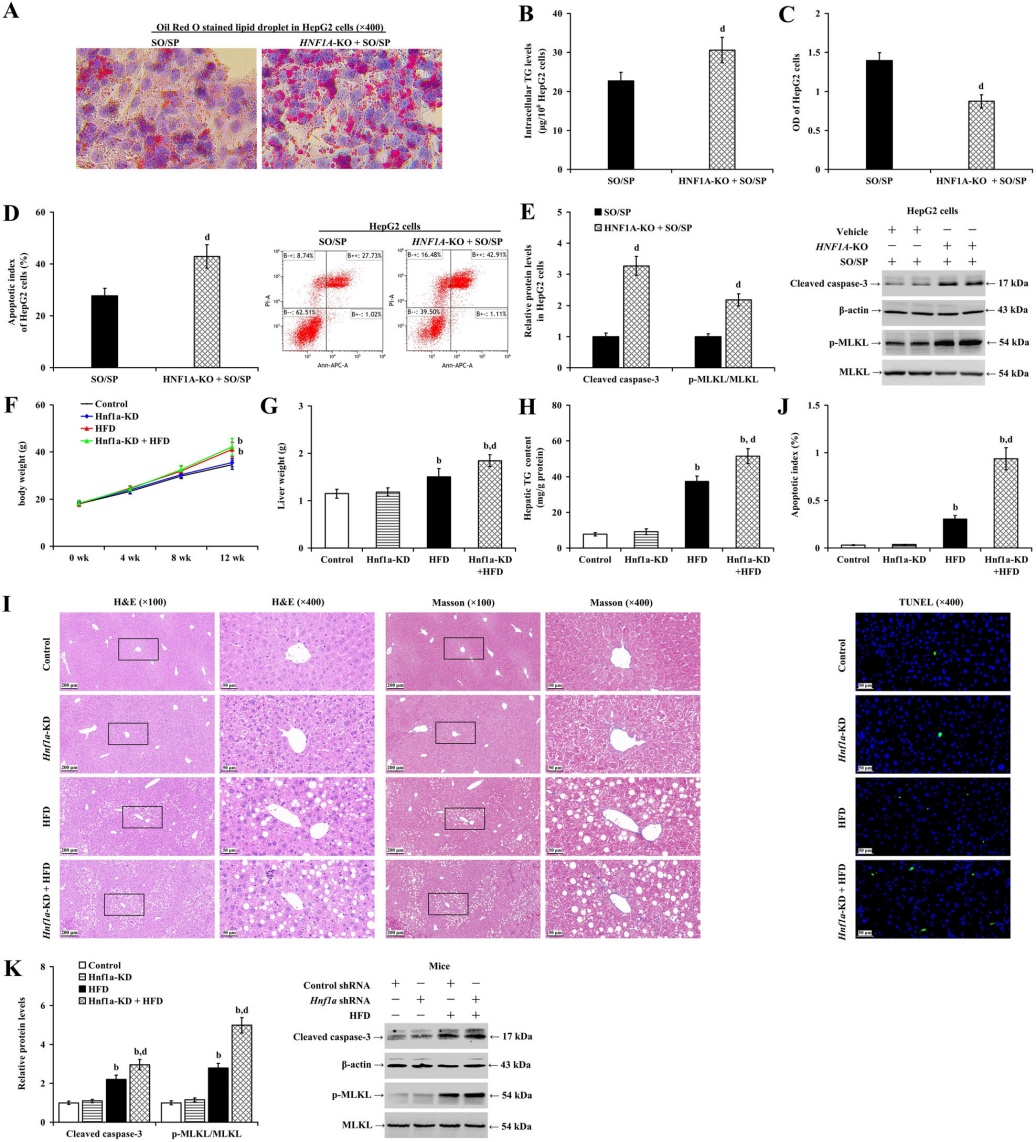
HNF1α depletion exacerbates hepatic steatosis. HepG2 cells with or without *HNF1A*-KO were treated with SO/SP. Under this condition, **A** intracellular lipid droplet accumulation, **B** TG content, **C** cell viability, **D** cell apoptosis, and **E** the expression of cleaved caspase-3 and p-MLKL were determined and compared. Mice (n = 12) with or without *Hnf1a*-KD were fed a normal diet or HFD. Their **F** body weight changes, **G** liver weights, and **H** hepatic TG content were evaluated and analyzed. **I** Representative images of H&E staining (left panels), and Masson staining (right panels) depicting the pathologic changes of mouse livers. **J** The apoptotic index of mouse liver cells calculated from the results of TUNEL staining (lower panel). **K** Analyzed data and representative images of western blotting revealing the relative expression of cleaved caspase-3 and p-MLKL. b *P* < 0.01, vs. control group; d *P* < 0.01, vs. SO/SP group or HFD group. HFD high-fat diet, KD knockdown, KO knockout, SO/SP sodium oleate and sodium palmitate.

To further explore the role of HNF1α in the progression of MASLD, we conducted experiments involving *HNF1A* knockout in HepG2 cells (5A–E) and *HNF1A* knockdown in LO2 cells (Supplementary Fig. 5A–E). The depletion of HNF1α led to increased SO/SP-induced lipid degeneration (Fig. 5A and Supplementary Fig. 5A) and intracellular TG levels (Fig. 5B and Supplementary Fig. 5B; *P* < 0.05). Additionally, it reduced cell viability and exacerbated the suppressive effects of SO/SP treatment on cell viability (Fig. 5C and Supplementary Fig. 5C; *P* < 0.05). The apoptosis index also increased following HNF1α depletion (Fig. 5D and Supplementary Fig. 5D; *P* < 0.01). Furthermore, we observed increased apoptosis- and necroptosis-related proteins, such as caspase-3 cleavage and MLKL phosphorylation, following HNF1α depletion (Fig. 5E and Supplementary Fig. 5E; *P* < 0.01).

We utilized a targeted approach to modulate the expression of HNF1α and then evaluated hepatic steatosis in control and MASLD mice. Upon *Hnf1a* knockdown, there was no significant difference in body weight (Fig. 5F; *P* > 0.05), liver weight (Fig. 5G; *P* > 0.05), hepatic TG content (Fig. 5H; *P* > 0.05), or serum ALT (Supplementary Fig. 5F; *P* > 0.05), TC (Supplementary Fig. 5G; *P* > 0.05), and TG levels (Supplementary Fig. 5H; *P* > 0.05) compared with the control group. Mild microvascular steatosis of hepatocytes was observed, with less than 5% of hepatocytes undergoing fatty degeneration, and no signs of hepatocyte necrosis, inflammatory cell infiltration, fibrosis, or apoptosis were evident (Fig. 5I and J; *P* > 0.05). *Hnf1a* knockdown did not significantly influence the protein expression of cleaved caspase-3 and p-MLKL in the mouse liver (Fig. 5K; *P* > 0.05). Compared to the HFD group at eight weeks, *Hnf1a* knockdown had no significant effect on body weight (Fig. 5F; *P* > 0.05), and serum ALT (Supplementary Fig. 5F; *P* > 0.05) and TG levels in the HFD mice (Supplementary Fig. 5H; *P* > 0.05). However, it significantly increased liver weight (Fig. 5G; *P* < 0.01), hepatic TG content (Fig. 5H; *P* < 0.01), hepatocyte fatty degeneration (Fig. 5I), the apoptotic index (Fig. 5J; *P* < 0.01), and serum TC levels (Supplementary Fig. 5G; *P* < 0.01). Moreover, knockdown of *Hnf1a* significantly increased HFD-fed the protein expression of cleaved caspase-3 and p-MLKL (Fig. 5K; *P* < 0.01). These findings suggest the role of hepatocyte HNF1α alleviating MASLD, possibly by diminishing hepatocyte apoptosis and necroptosis.

### Reshaped lipids in hepatic steatosis exhibit lipotoxicity, triggering hepatocyte apoptosis and necroptosis, and accelerating disease progression

**Fig. 6:**
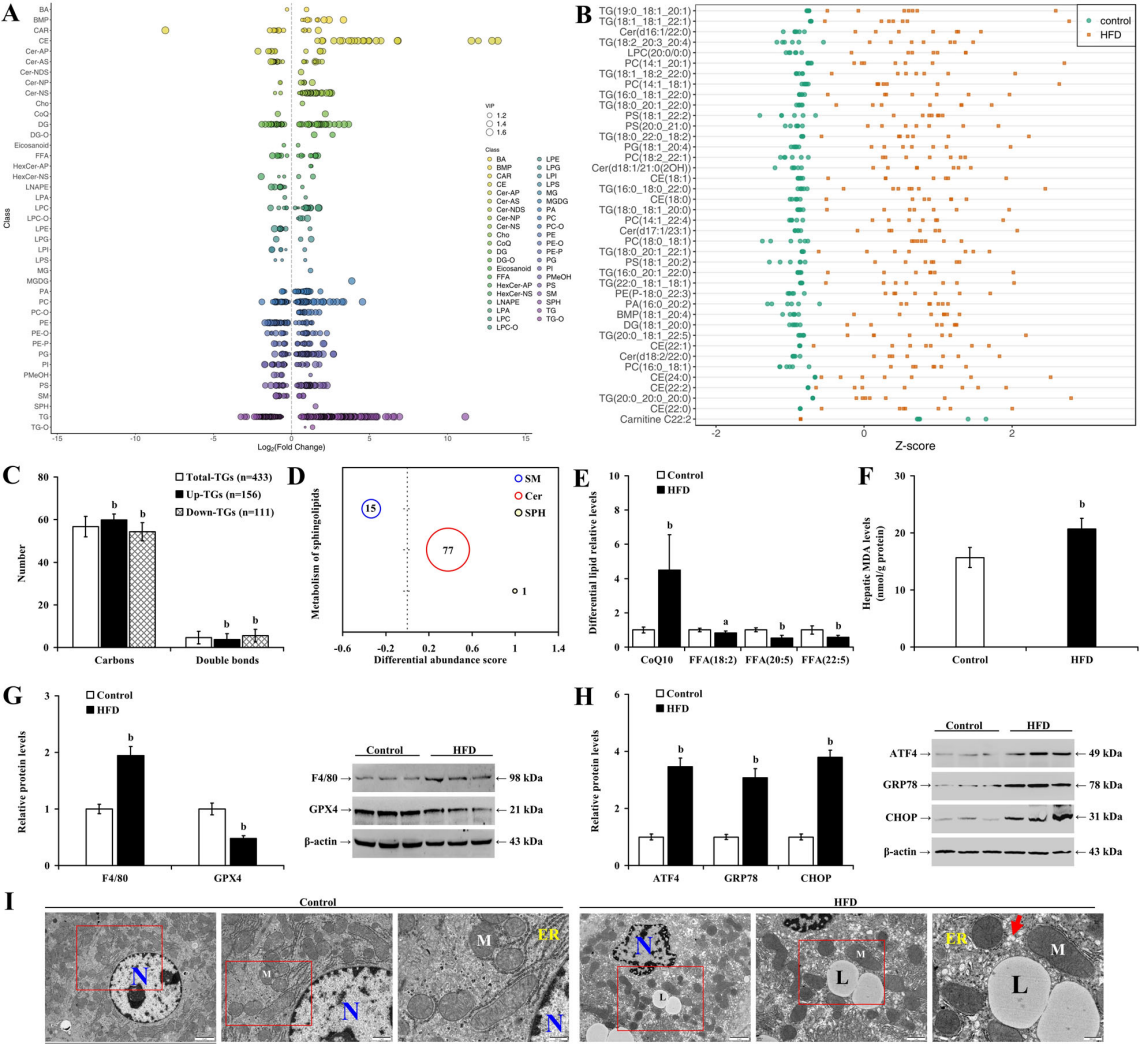
Reshaped lipids in hepatic steatosis exhibit lipotoxicity, triggering trigger hepatocyte apoptosis and necroptosis. LC-MS/MS measurement of liver tissue lipidomics demonstrating **A** differential lipids based on log_2_FC, **B** top 40 differential lipids by Z scores, **C** average carbon and double bond counts in differential TGs, **D** differential abundance scores for SM, Cer, and SPH (the diameter of the circles corresponds to the quantity of distinct lipid types, and the values on the horizontal axis indicate the proportion of upregulated differential lipids, with 1 representing a 100% increase) and **E** the levels of oxidized CoQ10 and FFAs. Comparisons of **F** MDA levels, and the relative expression levels of **G** GPX4, F4/80, **H** ATF4, GRP78, and CHOP in mice fed with normal diet or HFD. **I** Representative TEM images showing the hepatocyte ultrastructure. ER endoplasmic reticulum, L lipid droplet, M mitochondria, N nucleus, the arrow points to the dilated ER. a *P* < 0.05, b *P* < 0.01, vs. control group or total TGs group. FC fold change, HFD high-fat diet.

Lipidomics analysis revealed significant alterations in hepatic lipid composition in HFD-fed mice, marked by elevated TG levels (Fig. 6A and B). In mice with MASLD, 433 distinct TGs were identified, including 156 that were upregulated and 111 that were downregulated (Fig. 6C; *P* < 0.01). Notably, the upregulated TGs had higher carbon content and fewer double bonds than the total TG pool. Metabolites of sphingomyelin (SM), such as Cer and sphingosine (SPH), are crucial in LMP and inflammation. In HFD-fed mice, SM levels decreased, while Cer and SPH levels increased (Fig. 6D), suggesting that altered sphingolipids may contribute to hepatocyte damage. Furthermore, an increase in oxidized coenzyme Q10 and a decrease in FFAs 18:2, 20:5, and 22:5 (Fig. 6E; *P* < 0.05) indicated heightened lipid oxidation and a pro-inflammatory effect during MASLD progression. Concurrently, HFD feeding significantly raised hepatic MDA (Fig. 6F; *P* < 0.01) and GSSG levels (*P* < 0.01) but reduced GSH levels, ETC-CI and ETC-CIII activities (Supplementary Fig. 6A–D; *P* < 0.01). Additionally, eight weeks of HFD administration downregulated glutathione peroxidase 4 (GPX4) expression (*P* < 0.01), a key enzyme in preventing lipid peroxidation of cell membranes, while upregulating macrophage activation marker (F4/80) and ER stress-related proteins (ATF4, GRP78, and CHOP) in the liver (Fig. 6G and H; *P* < 0.01). HFD exposure also induced significant changes in hepatocyte ultrastructure in mouse liver, with cells showing condensed chromatin—suggestive of cellular distress and a potential shift towards apoptosis or necrosis. Noticeable mitochondrial shrinkage indicated mitochondrial dysfunction typically associated with cellular stress or damage, while dilated ER and accumulated lipid droplets in the cytoplasm pointed to ER stress and disrupted lipid metabolism, respectively, culminating in steatosis (Fig. 6I).

In HepG2 cells, SO/SP treatment increased the intracellular ROS, MDA, GSSG levels, and LMP, reducing the GSH levels and ETC-CI and ETC-CIII activities (Supplementary Fig. 6E–K; *P* < 0.01). Western blot analyses showed that SO/SP treatments decreased the expression of GPX4 (Supplementary Fig. 6L; *P* < 0.01) and elevated the expression of ATF4, GRP78, and CHOP (Supplementary Fig. 6M; *P* < 0.01).

**Fig. 7:**
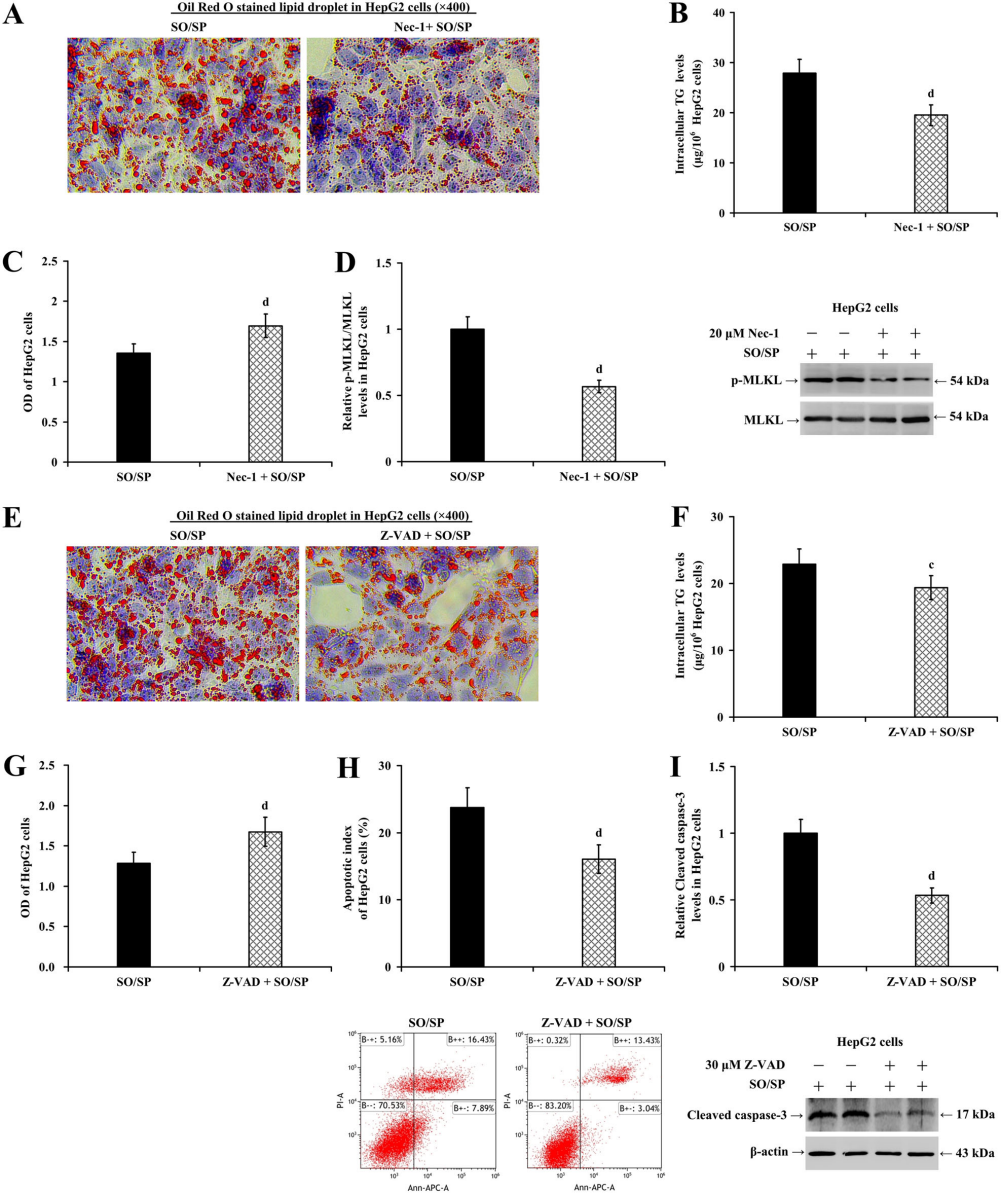
Necrostatin-1 and Z-VAD alleviate SO/SP-induced hepatocyte injury. HepG2 cells were treated with SO/SP in the presence or absence of necrostatin-1 (20 μM). Under this condition, **A** lipid droplet accumulation, **B** intracellular TG levels, **C** cell viability, and **D** the expression of p-MLKL were determined. HepG2 cells were treated with SO/SP in the presence or absence of Z-VAD (30 μM). Under this condition, **E** lipid droplet accumulation, **F** intracellular TG levels, **G** cell viability, **H** cell apoptosis, and **I** the expression of cleaved caspase-3 were determined. c *P* < 0.05, d *P* < 0.01, vs. SO/SP group. Nec-1 necrostatin-1, SO/SP sodium oleate and sodium palmitate, μM μmol/L, Z-VAD Z-Val-Ala-Asp(Ome)-fluoromethylketone.

To elucidate the roles of necroptosis and apoptosis in lipotoxicity-mediated cell injury induced by lipid accumulation in hepatocytes, gain-of-function assays were performed using necrostatin-1 (20 μM) or Z-VAD (30 μM, an inhibitor of caspases) in HepG2 (Fig. 7A–I) and LO2 cells (Supplementary Fig. 7A–I), both treated with or without SO/SP. Necrostatin-1 significantly reduced cell lipid degeneration (Fig. 7A and Supplementary Fig. 7A) and intracellular TG levels (Fig. 7B and Supplementary Fig. 7B; *P* < 0.01), restored cell viability (Fig. 7C and Supplementary Fig. 7C; *P* < 0.01), and reduced MLKL phosphorylation (Fig. 7D and Supplementary Fig. 7D; *P* < 0.01). Similarly, Z-VAD reduced cell lipid degeneration (Fig. 7E and Supplementary Fig. 7E) and intracellular TG levels (Fig. 7F and Supplementary Fig. 7F; *P* < 0.05), restored cell viability (Fig. 7G and Supplementary Fig. 7G; *P* < 0.01), and reduced cell apoptosis (Fig. 7H and Supplementary Fig. 7H; *P* < 0.01) and caspase-3 cleavage (Fig. 7I and Supplementary Fig. 7I; *P* < 0.01). These results suggest that necroptosis and apoptosis are programmed cell death modes associated with hepatocyte steatosis and may be involved in the progression of MASLD.

### ER stress-induced hepatic lipid remodeling mirrors processes in MASLD

**Fig. 8:**
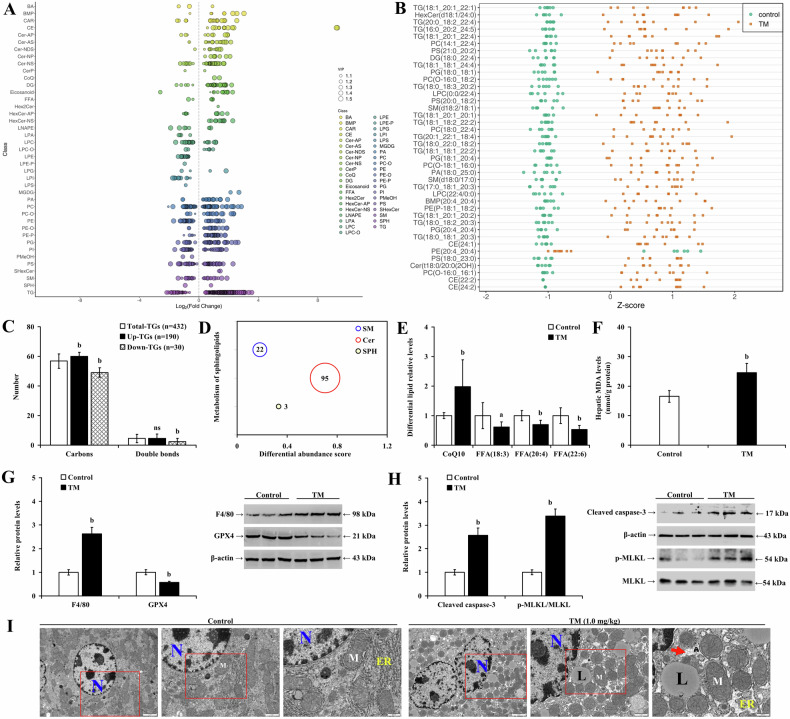
ER stress-induced hepatic lipid remodeling mirrors processes in MASLD. Lipidomic analysis of mouse livers by LC-MS/MS in TM-treated mice demonstrating **A** the differential lipids based on log_2_FC, **B** top 40 differential lipids by Z scores, **C** the average carbon and double bond counts in total and differential TGs, **D** differential abundance scores for SM, Cer, and SPH, and **E** the levels of oxidized CoQ10 and FFAs. In mice treated with or without TM, **F** MDA content and the expression of **G** GPX4, F4/80, **H** cleaved caspase-3, and p-MLKL were determined in the livers. **I** Representative TEM images showing the ultrastructure of the liver (red rectangle indicates the magnified area). ER endoplasmic reticulum, L lipid droplet, M mitochondria, N nucleus; arrow indicates dilated ER. ns *P* > 0.05, a *P* < 0.05, b *P* < 0.01, vs. control group or total TGs. FC fold change, TM tunicamycin.

To determine the impact of the ER stress on liver lipid remodeling, mice were treated with TM or DMSO for 8 weeks. Lipidomics analysis demonstrated significant alternations in hepatic lipid composition in TM-treated mice, resembling characteristics of the HFD-induced MASLD model. There was a significant increase in TG content (Fig. 8A, B), with 432 differential TG identified, comprising 190 upregulated and 30 downregulated lipids (Fig. 8C). TM-treatment also upregulated lipids included SMs, Cers, and SPH, with Cer showing the most significant increase (Fig. 8D), potentially leading to increased LMP, imbalanced inflammatory response, and mitochondrial dysfunction. TM induction also increased oxidated CoQ10 levels but decreased certain FFAs associated with anti-lipid peroxidation and anti-inflammation (Fig. 8E; *P* < 0.05). Moreover, TM induction increased liver MDA (Fig. 8F; *P* < 0.01), TG (Supplementary Fig. 8A; *P* < 0.01), and GSSG content, reduced hepatic GSH levels (Supplementary Fig. 8B, C; *P* < 0.01), and impaired ETC-CI and ETC-CIII activities (Supplementary Fig. 8D and E; *P* < 0.01). TM also downregulated GPX4 protein levels and upregulated F4/80, cleaved caspase-3 and p-MLKL protein expression (Fig. 8G, H; *P* < 0.05). Ultrastructure imaging of the hepatocytes revealed chromatin condensation, mitochondrial swelling, ER dilation, and lipid droplet deposition in the cytoplasm (Fig. 8I). These results indicate that ER stress induces hepatic lipid remodeling and exhibits potential lipotoxicity, resembling MASLD.

Using THA in hepatocytes, we also studied the cytotoxic characteristics of ER stress affecting cell apoptosis, and necroptosis. The results showed that THA significantly increased levels of intracellular ROS, MDA, GSSG, and LMP while downregulated the GSH levels and the activities of ETC-CI and ETC-CIII (Supplementary Fig. 8F–L; *P* < 0.01). Moreover, THA treatment also reduced the protein levels of GPX4 and promoted cell apoptosis and necroptosis as reflected by increased caspase-3 cleavage and MLKL phosphorylation, respectively (Supplementary Fig. 8M; *P* < 0.01). In addition, THA significantly increased apoptosis (Supplementary Fig. 8N; *P* < 0.01) and intracellular TG levels within 48 h (Supplementary Fig. 8O; *P* < 0.01). However, no typical lipid droplet formation was observed within HepG2 cells (Supplementary Fig. 8P).

### Impaired RelA signaling reprograms lipid metabolism and promotes lipid accumulation in hepatocytes with steatosis

Our findings suggest that lipid remodeling may result from altered expression of key metabolic-regulating proteins. For instance, medium-chain acyl-CoA dehydrogenase (MCAD), which is involved in fatty acid β-oxidation [43], and SREBP1, which promotes lipid production, are significant contributors. Changes in lipid export proteins, such as microsomal triglyceride transfer protein (MTP), may also lead to lipid accumulation [44, 45].

**Fig. 9:**
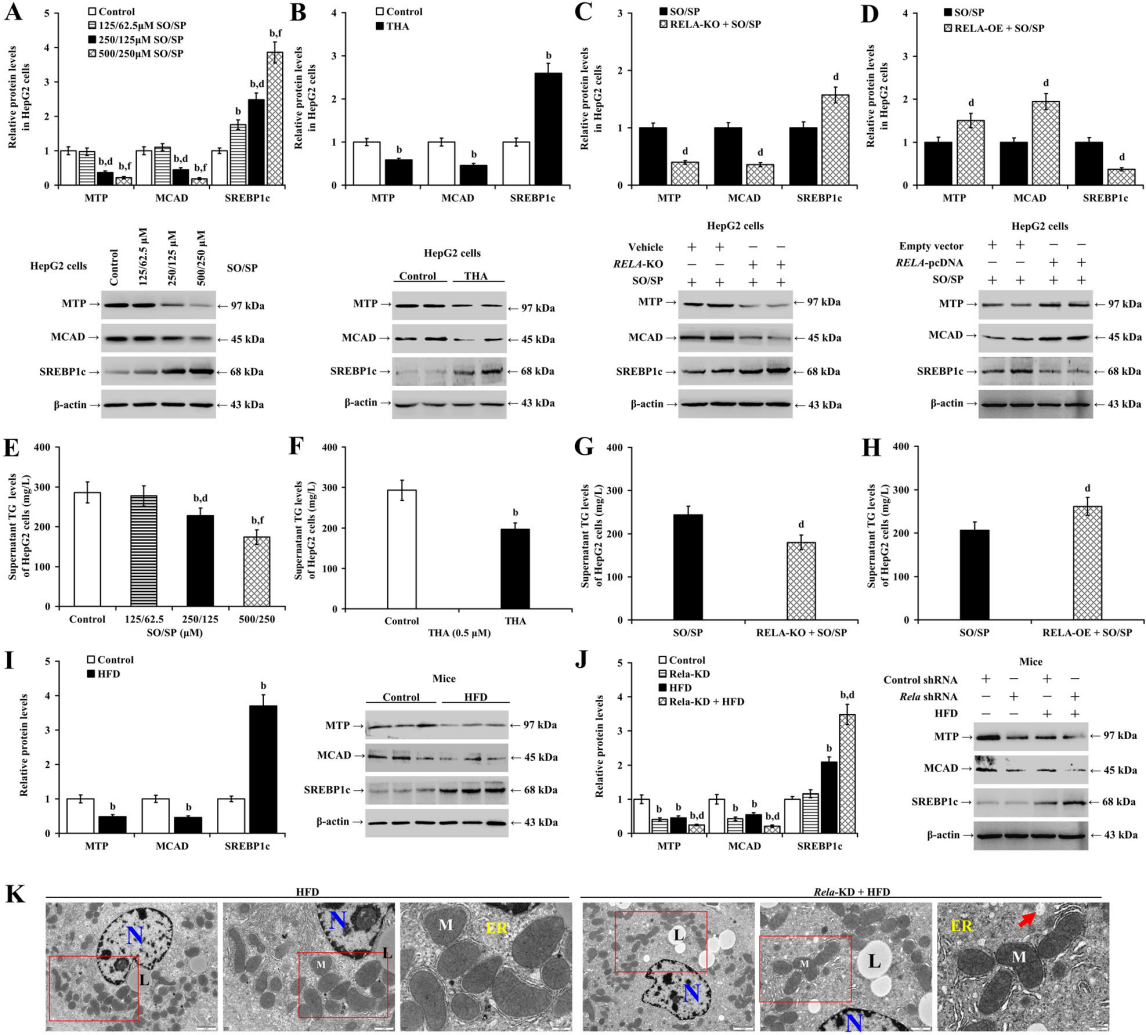
Impaired RelA signaling reprograms lipid metabolism and promotes lipid accumulation in hepatocytes with steatosis. The expression of lipid metabolism-related proteins (MTP, MCAD, SREBP1c) in HepG2 cells under the conditions of **A** SO/SP treatment, **B** THA treatment (0.5 μM), and SO/SP treatment with or without **C** *RELA*-KO or **D** *RELA*-OE. Extracellular TG levels were measured under the conditions of **E** SO/SP treatment, **F** THA treatment, and SO/SP treatment with or without **G** *RELA*-KO or (**H**) *RELA*-OE. **I** Analyzed data and representative images of Western blot analysis showing the expression of lipid metabolism-related proteins in the livers from mice fed with or without HFD. **J** Analyzed data and representative images of Western blot analysis showing the expression of lipid metabolism-related proteins in the livers from mice with or without *Rela*-KD under normal diet or HFD. **K** Representative TEM images showing the ultrastructure of mouse liver under *Rela*-KD. Red rectangles indicate magnified area. ER: endoplasmic reticulum; L lipid droplet, M mitochondria, N nucleus; arrow indicates dilated ER. b *P* < 0.01, vs. control group; d *P* < 0.01, vs. SO/SP group (125/62.5 μM), the corresponding SO/SP group, or HFD group; f *P* < 0.01, vs. SO/SP group (250/125 μM). HFD high-fat diet, KD knockdown, KO knockout, OE overexpression, SO/SP sodium oleate and sodium palmitate, THA thapsigargin, μM μmol/L.

Our in vitro investigations revealed that SO/SP and THA significantly downregulated MTP and MCAD expression (*P* < 0.01) while upregulating cleaved SREBP1 (SREBP1c) in HepG2 (Fig. 9A and B; *P* < 0.01) and LO2 cells (Supplementary Fig. 9A and B; *P* < 0.01). Unexpectedly, in *RELA*-knockout HepG2 cells and *RELA*-depleted LO2 cells, both MTP and MCAD expressions were decreased, and SREBP1c protein levels were increased following treatment with SO/SP (Fig. 9C and Supplementary Fig. 9C; *P* < 0.01). Conversely, overexpression of *RELA* in both HepG2 and LO2 cells increased the protein expression of MTP and MCAD and decreased the levels of SREBP1c (Fig. 9D and Supplementary Fig. 9D; *P* < 0.01).

SO/SP and THA significantly reduced extracellular TG levels in HepG2 (Fig. 9E, F; *P* < 0.01) and LO2 cells (Supplementary Fig. 9E, F; *P* < 0.01). The suppressive effects of SO/SP on TG secretion in hepatocytes were further enhanced by *RELA* knockout (Fig. 9G; *P* < 0.01) or knockdown (Supplementary Fig. 9G; *P* < 0.01), whereas *RELA* overexpression restored the extracellular TG levels in hepatocytes (Fig. 9H and Supplementary Fig. 9H; *P* < 0.01).

Moreover, mice fed with HFD for eight weeks showed significantly downregulated MTP and MCAD while upregulating SREBP1c in the liver (Fig. 9I; *P* < 0.01). Also, significantly reduced MTP and MCAD and increased SREBP1c expression in the liver were observed in TM-treated mice (Supplementary Fig. 9I; *P* < 0.01). Additionally, in MASLD mice, depleting RelA or HNF1α caused a more significant decrease in MTP and MCAD while increasing SREBP1c in the liver at protein levels (Fig. 9J and Supplementary Fig. 9J; *P* < 0.01). Furthermore, TEM analysis revealed that the knockdown of *Rela* or *Hnf1a* exacerbated mitochondrial shrinkage, ER dilation, and lipid droplet deposition in the hepatocytes in HFD-fed mice (Fig. 9K and Supplementary Fig. 9K).

To investigate the impact of SREBP1 on lipogenesis during hepatocyte steatosis, fatostatin (20 μM) was administered into HepG2 cells with SO/SP treatment. The results showed that fatostatin significantly decreased cell lipid accumulation (Supplementary Fig. 10A), intracellular TG content (Supplementary Fig. 10B; *P* < 0.01), and cell viability (Supplementary Fig. 10C; *P* < 0.01) while reducing SREBP1c expression. Meanwhile, it increased caspase-3 cleavage, MLKL phosphorylation (Supplementary Fig. 10D; *P* < 0.01), and cell apoptosis (Supplementary Fig. 10E; *P* < 0.01).

### *Impaired RelA signaling* remodels the ER stress response and exacerbates lipid toxicity during hepatocyte steatosis

**Fig. 10:**
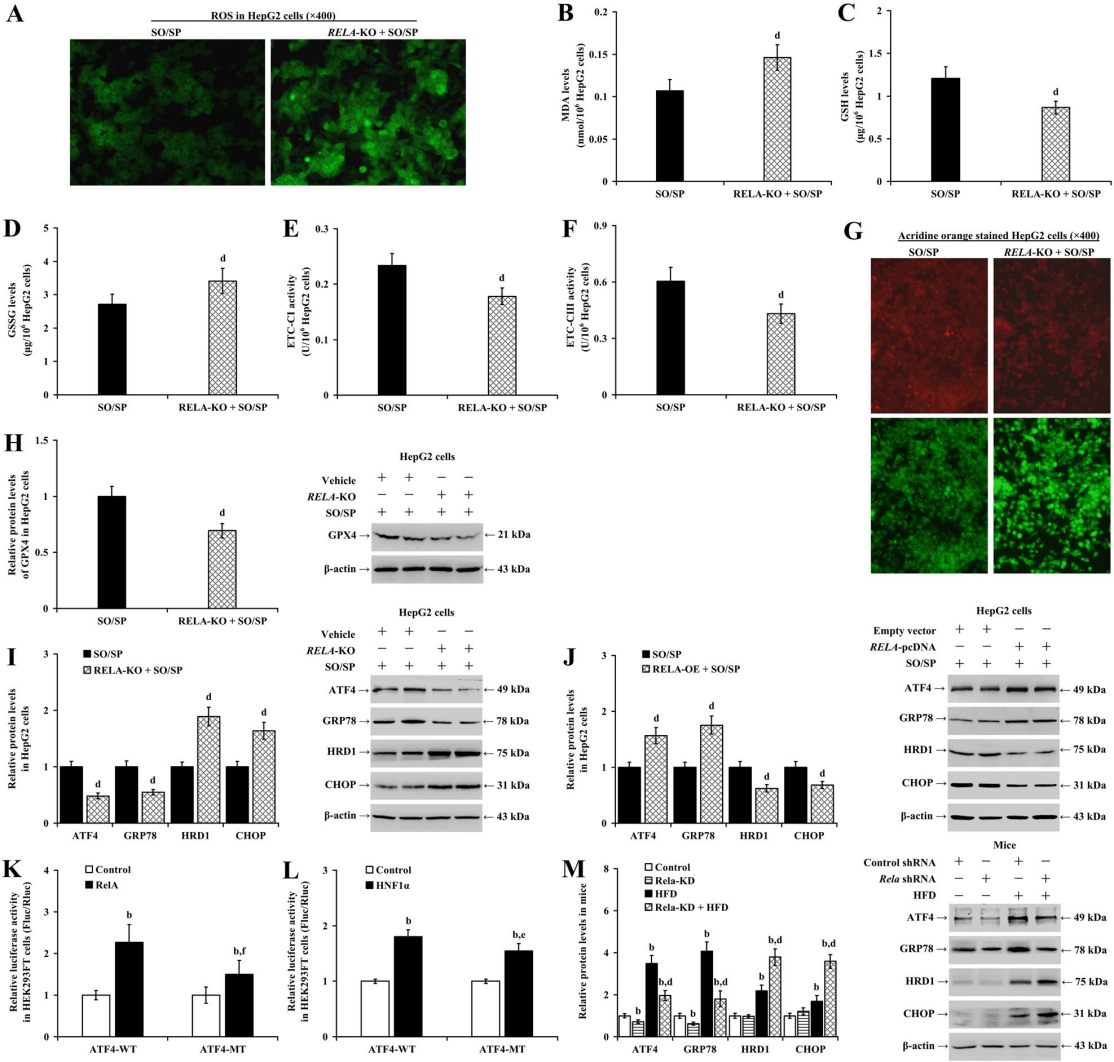
Impaired RelA signaling remodels the ER stress response and exacerbates lipid toxicity during hepatocyte steatosis. HepG2 cells with or without *RELA*-KO were treated with SO/SP. Under this condition, **A** ROS levels, **B** MDA levels, **C** GSH levels, **D** GSSG levels, **E** ETC-CI activity, **F** ETC-CIII activity, **G** LMP status, and **H** GPX4 expression were evaluated. Expression of ER stress markers in SO/SP-treated HepG2 cells with **I** *RELA*-KO or **J** *RELA*-OE. Results of luciferase reporter assay demonstrating **K** the interaction between RelA and *ATF4* promoter region and **L** the interaction between HNF1α and *ATF4* promoter region. **M** Analyzed data and representative images of Western blot analysis showing the expression of ER stress-related proteins in control or MASLD mouse livers with or without *Rela*-KD. b *P* < 0.01, vs. control group; d *P* < 0.01, vs. SO/SP group or HFD group, e *P* < 0.05, f *P* < 0.01, vs. *ATF4*-WT group. KO knockout, OE overexpression, HFD high-fat diet, SO/SP sodium oleate and sodium palmitate.

Our data illustrated that knockout (Fig. 10A–H) or knockdown (Supplementary Fig. 11A–H) of *RELA* enhanced the promotive effects of SO/SP on ROS (Fig. 10A and Supplementary Fig. 11A), MDA (Fig. 10B and Supplementary Fig. 11B; *P* < 0.01), GSSG (Fig. 10D and Supplementary Fig. 11D; *P* < 0.01), and LMP levels (Fig. 10G and Supplementary Fig. 11G) and their suppressive effects on GSH levels (Fig. 10C and Supplementary Fig. 11C; *P* < 0.01), ETC-CI and ETC-CIII activities (Fig. 10E and F and Supplementary Fig. 11E and F; *P* < 0.01), and GPX4 expression levels in hepatocytes (Fig. 10H and Supplementary Fig. 11H; *P* < 0.01).

In addition, knockout (Fig. 10I; *P* < 0.01) or knockdown of *RELA* (Supplementary Fig. 11I; *P* < 0.01) decreased the expression of SO/SP-upregulated ATF4 and GRP78 but increased HRD1 (indicative of ER-associated degradation (ERAD) activation) and CHOP protein levels. In contrast to *RELA* knockout, *RELA* overexpression increased the protein expression of ATF4 and GRP78 but decreased HRD1 and CHOP in HepG2 (Fig. 10J; *P* < 0.01) and LO2 cells (Supplementary Fig. 11J; *P* < 0.01). Consistent with our observations in *RELA*-knockout cells, depletion of HNF1α resulted in a decrease in SO/SP-induced ATF4 and GRP78 expression, and increases in HRD1 and CHOP levels without affecting RelA expression in HepG2 cells (Supplementary Fig. 11K; *P* < 0.01).

We performed a dual luciferase reporter assay to evaluate the transcription activation of the RelA signaling on the *ATF4* promoter. The results showed that when the wild-type *ATF4* promoter reporter vector (*ATF4*-WT) was co-transfected with the constructs overexpressing *RELA or HNF1A*, the Fluc/Rluc ratio increased compared to the control group (Fig. 10K and L; *P* < 0.01). While this upregulated luciferase activity could be compromised when the promoter was mutated (*ATF4*-MT, *P* < 0.05), suggesting that RelA and HNF1α may co-activate the transcription of *ATF4*.

Our in vivo study revealed that in mice fed with normal diet, silencing *Rela* reduced the expression of ATF4 and GRP78 in the liver. Meanwhile, in MASLD mice, *Rela* knockdown caused a reduced magnitude of elevation of ATF4 and GRP78 in the liver. It also increased the expression of HRD1 and CHOP (Fig. 10M; *P* < 0.01). *Hnf1a* knockdown led to a significant decrease in the protein expression of ATF4 and GRP78 in the mouse liver (Supplementary Fig. 11L; *P* < 0.01) without affecting other protein expression (*P* > 0.05). Compared to the HFD group at eight weeks, knockdown of *Hnf1a* significantly decreased the expression of ATF4 and GRP78 in the liver after HFD (Supplementary Fig. 11L; *P* < 0.01) but increased the protein expression of HRD1 and CHOP (*P* < 0.01).

## Discussion

This study explored the progression of MASLD driven by a detrimental cycle of impaired RelA signaling and disrupted lipid metabolism in hepatocytes. During MASLD progression, disruption of RelA signaling within hepatocytes is evident. Lipid accumulation inhibits the reciprocal transcriptional activation between RelA and HNF1α, thereby impairing RelA signaling and further promoting the progression of MASLD. Further investigation revealed that impaired RelA signaling diminishes the expression of ATF4 and GRP78, reshaping hepatocyte’s responses to ER stress and disrupting lipid metabolism. These effects lead to increased lipid accumulation and lipid toxicity, which further aggravate hepatocyte apoptosis and necroptosis.

RelA and HNF1α are transcription factors that respond to various stimuli. The RelA (NF-κB p65) subunit, containing a transactivation domain, is a crucial regulator of NF-κB transcriptional activity [46]. In this study, we observed decreased expression of RelA and HNF1α in steatotic hepatocytes. Conversely, their expression was upregulated in TM-induced mouse livers and THA-treated hepatocytes. These opposing changes may depend on the presence of steatosis in hepatocytes.

Previous studies have shown that HNF1α activates the transcription of *Rela*; the decreased expression of HNF1α in fatty liver suggests a potential mutual transcriptional activation between HNF1α and RelA. However, this interaction may be inhibited during hepatocyte steatosis. Knockdown of RelA in hepatocytes resulted in the downregulation of HNF1α while overexpressing RelA mitigated these effects. Similarly, the knockdown of HNF1α led to decreased expression of RelA. Dual-luciferase reporter assays confirmed the transcriptional activation between RelA and HNF1α, highlighting the lack of reciprocal transcriptional activation between these factors in hepatocyte steatosis.

RelA signaling performs various roles in liver diseases, with diverse functions across different liver cell types. For instance, it promotes tumorigenesis in cancer cells [47], regulates immune stimulation, cell adhesion, chemotaxis, and differentiation in immune cells, and protects against cell apoptosis or injury in hepatocytes [48, 49]. *RELA* fusion mutations can activate NF-κB abnormally, contributing to tumor formation. HNF1α has been found to promote lipid metabolism homeostasis [50–52]. In the current study, we observed that reducing RelA and HNF1α expression in hepatocytes worsened MASLD while overexpressing RelA attenuated hepatocyte fat degeneration, apoptosis, and necroptosis. These results confirm that impaired RelA signaling in hepatocytes drives MASLD progression, possibly by aggravating hepatocyte apoptosis and necroptosis. RelA-mediated NF-κB signaling activation promotes immune cell recruitment and activation, exacerbating hepatic steatosis [53, 54]. This study focused on hepatocytes and investigated the role of RelA signaling in MASLD progression from a different perspective, broadening our understanding of RelA signaling in MASLD.

In non-fat tissue, lipid accumulation can lead to lipotoxicity, damaging cellular organelles chronically [55]. This condition may activate hepatocyte apoptosis and necroptosis due to excessive TG accumulation and increased levels of toxic lipids, such as peroxidized lipids and Cers [56, 57]. Accumulation of TGs can result in ER stress, and toxic lipids can disrupt ETC complexes I and III, lowering ATP production and elevating ROS levels [58, 59]. Excessive ROS overwhelms the antioxidant capacity of GSH, leading to oxidative damage. In our study, liver lipids, particularly TGs, were significantly remodeled in MASLD mice. The remodeled TGs had longer carbon chains and fewer double bonds, indicating reduced fatty acid oxidation and enhanced lipid peroxidation in MASLD. This was supported by our TEM observations of ER dilation and mitochondrial condensation in hepatocytes of experimental MASLD mice.

Moreover, we observed a decrease in polyunsaturated FFAs, GSH, ETC-CI/CIII activity, and GPX4 expression, along with an increase in oxidized CoQ10, MDA, GSSG, and ER-related protein expression, supporting the occurrence of ER stress and MOS. In addition, we found that the level of Cers, and F4/80 expression were increased in MASLD mice along with LMP in steatotic hepatocytes, suggesting impaired lysosomal function and exacerbated inflammation, which aligns with previous studies [60, 61]. We also noticed decreased phosphatidyl inositol levels, which have anti-inflammatory properties [62, 63]. Together, these disrupted oxidation/antioxidant homeostasis, increased Cers and LMP, and intensified inflammatory response can all trigger apoptosis and necroptosis [64, 65]. Additionally, necrostatin-1 and Z-VAD alleviate SO/SP-induced hepatocyte injury, supporting that reshaped lipids trigger hepatocyte apoptosis and necroptosis, thereby accelerating MASLD progression.

The ER and mitochondria are pivotal in lipid metabolism [66]. Excessive accumulation of TGs in the liver triggers ER stress in hepatocytes, which reciprocally impairs mitochondrial function and exacerbates lipid metabolism disorders. ER dysfunction initiates the release of cytoplasmic Ca^2+^, leading to calcium overload. This overload further compromises the function of the ETC, intensifying the production of ROS and exacerbating ER stress [67].

An intriguing observation was the TM-induced ER stress in mouse liver. Although no typical pathological changes indicative of liver lipid degeneration were observed, scattered lipid droplets were present. This suggests that ER stress-mediated lipid accumulation does not exhibit the typical changes associated with hepatocyte steatosis, possibly due to the excessive dispersal of lipid droplets. The lack of typical fatty hepatocytes under ER stress conditions may also result from their susceptibility to apoptosis or necroptosis, leading to premature removal before typical lipid degeneration can form. This phenomenon may partially explain the observed increases in serum TG levels in mice following TM induction, as the accumulated lipids in acute damaged hepatocytes could be released into the bloodstream.

Further hepatic lipidomic analysis revealed lipid remodeling similar to that observed in HFD-fed mice. We also observed TG accumulation, MOS, and heightened LMP in steatotic hepatocytes or THA-treated hepatocytes, suggesting that ER stress drives lipid remodeling in MASLD with the involvement of MOS.

The primary approach by which RelA affects lipid metabolism may be regulating ER stress. The lipids accumulate in the liver when the lipids uptake, and synthesis overwhelm its eliminating capacity. In a chemical-induced liver injury model, the liver displayed activated ER stress, increased hepatic TG content, upregulated SREBP1c, and downregulated MTP and MCAD [68]. Our data consistently revealed reduced MTP and MCAD and increased SREBP1c in MASLD in vitro and in vivo. Moreover, ER stress reduced the expression of MTP and MCAD and boosted SREBP1c expression in hepatocytes without steatosis. These results indicate that ER stress increases lipid synthesis, decreases lipid oxidation, and impedes lipid excretion, potentially leading to lipid accumulation in hepatocytes. The UPR of the ER affects protein expression through several mechanisms: the phosphorylation of eIF2α [69], IRE1-dependent decay [70], and ERAD [71]. The reduction in MTP and MCAD expression by ER stress may relate to the role of the UPR in regulating the intracellular protein load. Furthermore, this study demonstrated that ER stress damages the ETC in mitochondria, increasing ROS production. This suggests that ER stress impedes lipid oxidation and accelerates lipid peroxidation.

Additional studies have confirmed that SREBP1 is associated with ER stress-mediated lipid synthesis [72, 73]. We inhibited SREBP1 with fatostatin to investigate its impact on hepatocyte steatosis. Interestingly, although fatostatin reduced TG production upon SO/SP stimulation, it exacerbated hepatic cell death. This may be due to inhibiting SREBP1’s anti-cell death and pro-survival functions [74, 75]. These findings suggest that using fatostatin to inhibit SREBP1 to combat hepatic steatosis warrants cautious consideration.

We also observed that reducing RelA or HNF1α expression decreased MTP and MCAD and increased SREBP1c expression in hepatocytes. Conversely, overexpressing RelA increased MTP and MCAD and decreased SREBP1c. Ultrastructure of hepatocytes revealed that upon RelA or HNF1α depletion, increased lipid droplets and ER dilation were observed in HFD-fed mice. These findings imply that impaired RelA signaling exacerbates lipid metabolism abnormalities, potentially associated with ER dysfunction. Notably, silencing RelA or HNF1α increased HRD1 and CHOP, while overexpressing RelA decreased HRD1 and CHOP, suggesting that ERAD and ER stress-related apoptosis were promoted following RelA inhibition. ER stress-induced apoptosis represents a decompensatory response attributable to the UPR’s inability to maintain cellular homeostasis, and its occurrence correlates with the severity of ER stress. Therefore, these results substantiate that impaired RelA signaling exacerbates ER stress-induced lipid metabolism disorder.

PERK, IRE1, and ATF6 signaling mediate UPR under ER stress. Inhibition of these processes can hinder cellular homeostatic recovery, leading to sustained ER stress and potential hepatocellular lipid degeneration [76, 77]. Our previous study showed that HNF1α regulates the expression of ATF4 and GRP78 [37]. In this study, we observed that silencing either RelA or HNF1α led to the downregulation of ATF4 and GRP78, paradoxically promoting hepatocyte apoptosis. Conversely, overexpressing RelA upregulated ATF4 and GRP78, alleviating hepatocyte apoptosis. This suggests that RelA signaling in MASLD may reshape hepatocyte responses to ER stress. Due to the lack of HNF1α expression in specific cells, it is still not completely determined whether HNF1α is a required pathway for RelA to regulate ATF4 transcription. We further analyzed the regulatory effect of RelA on ATF4 transcription, and the results of dual-fluorescence reporter gene detection showed that RelA promotes the transcription of ATF4, suggesting that RelA and HNF1α jointly activate the transcription of ATF4, thereby affecting UPR under ER stress.

Recent studies have illustrated the multifaceted role of GRP78 in metabolic processes. Notably, GRP78 has been shown to promote heat production [78]. When overexpressed, it can inhibit the activation of lipogenic genes, thereby alleviating lipid peroxidation and hepatic steatosis [79]. However, the role of ATF4 in MASLD is complex. For instance, using salubrinal to enhance the expression of p-eIF2α and ATF4 mitigates obesity-induced hepatic steatosis in mice [80].

Conversely, ATF4 also promotes liver fat degeneration [81, 82]. Additionally, mice with liver-specific HRD1 deficiency exhibit increased energy expenditure and resistance to HFD-induced obesity and hepatic steatosis [83]. In leptin receptor-*db/db* mice, overexpression of *Hrd1* has been shown to ameliorate liver steatosis [84]. These variations in ATF4 function suggest that it may directly regulate lipid metabolism, act as a biomarker for the activation of the UPR, and participate in the maintenance of ER homeostasis. By modulating the ER stress response, ATF4 indirectly influences lipid metabolism. Furthermore, ATF4 plays a crucial role in managing stress responses and mitochondrial adaptation, maintaining redox balance, and promoting cell survival, as extensively documented in numerous studies [85–87].

This study has some limitations that should be noted. The RelA signaling pathway may be crucial in the interaction between ER stress and the MOS. Moreover, the metabolic changes in cholesterol and ether phospholipids during lipid remodeling are notable, yet their specific significance in MASLD progression remains understood. These unresolved issues await further in-depth research in the future.

## Conclusions

**Fig. 11:**
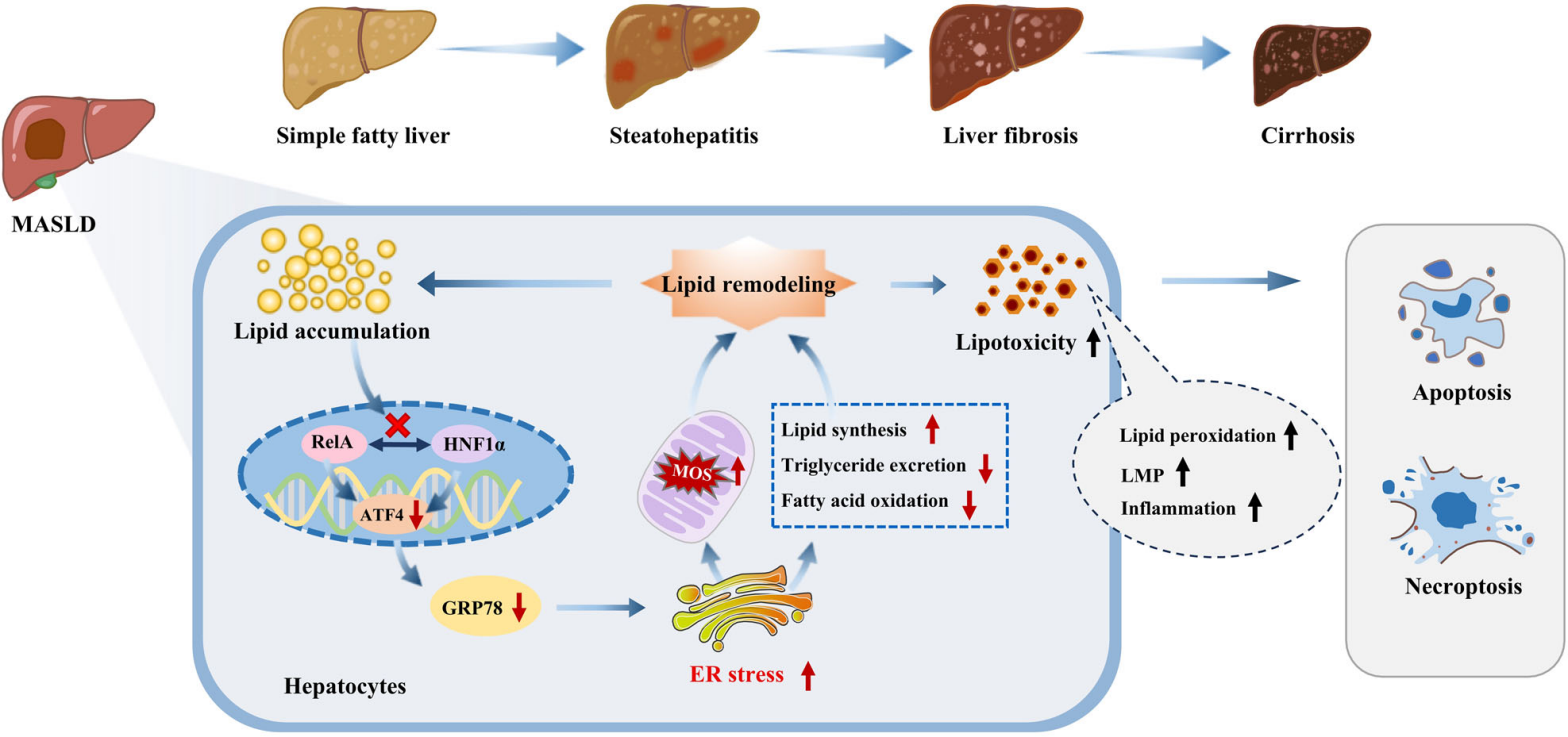
Diagram showing how the hepatocyte RelA signaling regulates MASLD progression. Impaired RelA signaling and disrupted lipid metabolism form a detrimental feedback loop in hepatocytes, advancing MASLD. Accumulated lipids suppress RelA signaling, reducing ATF4 and GRP78, worsening ER stress, and reshaping lipid metabolism, leading to apoptosis and necroptosis.

In MASLD, a detrimental cycle emerges involving impaired RelA signaling and disrupted lipid metabolism in hepatocytes, which drives disease progression. Lipid accumulation within hepatocytes leads to compromised RelA signaling. This impairment results in decreased expression of ATF4 and GRP78, reshaping ER stress responses. These changes contribute further to lipid accumulation and lipotoxicity. This remodeled lipid profile then activates pathways leading to hepatocyte apoptosis and necroptosis, as illustrated in Fig. 11.

## Method

### Animal experiments

All animal studies were approved by the Animal Laboratory Studies Ethics Review Committee of Zunyi Medical University (ZMU21-2107-003 and ZMU22-2303-029). Male C57BL/6 mice (5-6 weeks old, 20.63 ± 1.89 g) were purchased from the Animal Center of Zunyi Medical University (Guizhou, China). Unless noted otherwise, there were at least 12 mice/group in all studies. The mice were randomly assigned to different experimental conditions using a random number table to ensure unbiased allocation.

### Induction of MASLD in mice

To induce MASLD, mice were fed a high-fat diet (HFD, 20% protein, 20% carbohydrate, 60% fat). The mice were randomly divided into two groups (n = 12). The control group was fed a normal diet, and the other group was fed the HFD for eight weeks. The successful establishment of the MASLD mouse model was confirmed by assessing changes in serum biochemical indicators, liver TG content, and tissue pathology.

### Rela or Hnf1a knockdown in vivo

To explore the impact of RelA on MASLD, 48 mice were randomly assigned to four groups based on different small hairpin RNA (shRNA) sequences and diet conditions: a control group (control shRNA, normal diet), *Rela*-knockdown (KD) group (*Rela-*targeting shRNA, normal diet), an HFD group (control shRNA, HFD), and *Rela*-KD + HFD group (*Rela-*targeting shRNA, HFD). Additionally, another set of 48 mice was divided into four groups to investigate the role of HNF1α in MASLD: a control group (control shRNA, normal diet), *Hnf1a*-KD group (*Hnf1a*-targeting shRNA, normal diet), an HFD group (control shRNA, HFD), and *Hnf1a*-KD + HFD group (*Hnf1a*-targeting shRNA, HFD).

Each mouse received a tail vein injection of recombinant adeno-associated virus (serotype 8, Genechem, China) containing the appropriate shRNA (ranging from 5 × 10^10^-1 × 10^11^ viral gene copies per mouse) to induce gene silencing. The specific shRNA sequences used are detailed in Table 1. The mice were placed on an HFD four weeks after transduction to establish the MASLD model. At the end of each designated stage of the experiment, blood samples were collected under anesthesia, and liver samples were harvested post-euthanasia.

**Table 1:** shRNA sequences used in vivo.

Parameter		5ʹ to 3ʹ
RelA	Target sequence	GGACCTATGAGACCTTCAAGA
	*Rela* shRNA	GGACCTATGAGACCTTCAAGA CGAATCTTGAAGGTCTCATAGGTCC
	Control shRNA	AAACGTGACACGTTCGGAGAACGAATTCTCCGAACGTGTCACGTTT
HNF1α	Target sequence	GCGATGAGCTGCCAACTAAGA
	*Hnf1a* shRNA	GCGATGAGCTGCCAACTAAGA CGAA*TCTTAGTTGGCAGCTCATCGC*
	Control shRNA	AAACGTGACACGTTCGGAGAACGAATTCTCCGAACGTGTCACGTTT

### Induction of ER stress in the livers of mice

To induce ER stress in vivo, mice were randomly divided into two groups and administrated either tunicamycin (TM, Sigma, USA) or dimethysulfoxide (DMSO) via an intraperitoneal injection twice a week for eight weeks (n = 12). The groups included a control group (normal diet, DMSO) and TM group (normal diet, TM; 1.0 mg/kg). TM is a frequently utilized ER stress inducer both in vivo and in vitro. The ER stress is triggered by TM when it hampers N-linked glycosylation and obstructs N-glycosidic protein-carbohydrate connections, accumulating misfolded proteins within the ER lumen [88].

Mice were fasted for 6 h before each injection and were anesthetized during the procedure. Blood and liver samples were collected 24 h after the final injection.

### Measurements of serum alanine aminotransferase (ALT), total cholesterol (TC), and TG levels

Blood samples were collected from anesthetized mice, and the serum levels of ALT, TC, and TG were measured using a Beckman Coulter autoanalyzer (AU5800, Beckman Coulter, USA) per standard protocol [89]. Specifically, the serum levels of ALT were quantified using the rate method. TC was measured using the cholesterol oxidase method. TG was assessed using the enzymatic method.

### Histopathological analysis

Liver tissues were fixed with 4% formaldehyde, embedded in paraffin, and sectioned to a thickness of 4 μm. These sections were then deparaffinized and stained using hematoxylin and eosin (H&E) for general histology. For fibrosis detection, Masson’s trichrome staining was performed, involving potassium dichromate, iron hematoxylin to stain muscle fibers, cellulose, and red blood cells, followed by acid fuchsin, phosphomolybdic acid, and aniline blue to specifically stain collagen fibers. The stained liver tissue sections were imaged using a panoramic slice scanner (3DHISTECH CaseViewer, Pannoramic SCAN, Hungary). Image analysis was conducted using CaseViewer v2.4 software at various magnifications, ranging from 1× to 1000×, with particular focus on the target areas under 100× to 400× magnification.

### Terminal deoxynucleotidyl transferase-mediated deoxyuridine triphosphate nick end labeling (TUNEL) assay

A TUNEL assay kit (11684817910; Roche,) assessed apoptosis in liver tissue sections, following the manufacturer’s instructions. Six areas were randomly selected on each slide to calculate the apoptotic index. The apoptotic index was calculated using the formula: apoptotic index = (number of positive cells / total number of cells) × 100%. This method provides a quantitative measure of hepatocyte apoptosis within the tissue sections.

### Liposome detection

Lipid metabolism was analyzed using a liquid chromatography (LC)-tandem mass spectrometry system consisting of an ExionLC system equipped with a QTRAP mass spectrometer (SCIEX). The triple quadrupole MS with multiple reaction monitoring (MRM) modes was used to analyze the extracted samples, screen differential lipids, and elucidate metabolic pathways. Differential metabolites were identified through a two-group analysis utilizing variable importance in projection (VIP; >1) and *P*-value (<0.05) criteria. VIP values were obtained using Orthogonal Partial Least Squares-Discriminant Analysis (OPLS-DA), incorporating score plots and permutation plots generated using the R package MetaboAnalystR. To prevent overfitting, data underwent log-transformation (log2) and mean-centering before conducting OPLS-DA, with a permutation test (200 permutations) applied during the analysis. Furthermore, data were standardized using unite variance scaling (UV; Z-score normalization), resulting in standardized data with a mean of 0 and a standard deviation of 1. In addition, the differential abundance score was calculated as the difference between upregulated and downregulated differential lipids divided by annotated lipids within the pathway, represented by circle size. The relevant abbreviations are detailed in Table 2.

**Table 2:** List of abbreviations.

Abbreviations	Full name
ALT	alanine aminotransferase
APC	allophycocyanin
ATF4	activating transcription factor 4
ATF6	activating transcription factor 6
BA	bile acid
BMP	diacylglycerol phosphate ester
BSA	bovine serum albumin
CAR	carnitine
CCK-8	Cell Counting Kit-8
CE	cholesterol ester
Cer	ceramide
Cer-AP	N-(2-hydroxyacyl)-phytosphingosine
Cer-AS	N-(2-hydroxyacyl)-sphingosine
Cer-NDS	ceramides containing nonhydroxy fatty acid and dihydrosphingosine
Cer-NP	N-acyl-phytosphingosine
Cer-NS	N-acyl-sphingosine
CerP	1-phosphatidylserine
Cho	cholesterol
CHOP	CCAAT-enhancer-binding protein homologous protein
CoQ	coenzyme Q
DCFH-DA	2′, 7′-dichlorodihydrofluorescein diacetate
DG	diglyceride
DG-O	diglyceride (ether bond)
DGDG	digalactosyl diglyceride
DMSO	dimethysulfoxide
eIF2α	eukaryotic translational initiation factor 2 alpha
ER	endoplasmic reticulum
ERAD	ER-associated degradation
ETC	electron transport chain
ETC-CI	electron transport chain complex I
ETC-CIII	electron transport chain complex III
FFA	free fatty acid
Fluc	firefly luciferase
FOXA3	forkhead box A3
GRP78	78 kDa glucose-regulated protein
GPX4	glutathione peroxidase 4
GSH	reduced glutathione
GSSG	oxidized glutathione
H&E	hematoxylin and eosin
Hex2Cer	dihexosyl ceramide
Hex3Cer	trihexosyl ceramide
HexCer-AP	hexosyl ceramide-AP
HexCer-NS	hexosyl ceramide-NS
HFD	high-fat diet
HNF1α	hepatocyte nuclear factor 1 alpha
HRD1	3-hydroxy-3-methyl glutaryl coenzyme A reductase degradation 1 homolog
PI	propidium iodide
IRE1	inositol requiring enzyme 1
KD	knockdown
KO	knockout
LC	liquid chromatography
LNAPE	N-acyl lysophosphatidylethanolamine
LPA	lysophosphatidic acid
LPC	lysophosphatidylcholine
LPC-O	lysophosphatidylcholine (ether bond)
LPE	lysophosphatidylethanolamine
LPE-P	lysophosphatidylethanolamine (vinyl ether bond)
LPG	lysophosphatidylglycerol
LPI	lysophosphatidylinositol
LPS	lysophosphatidylserine
LMP	lysosomal membrane permeabilization
MAFLD	metabolic-associated fatty liver disease
MASLD	metabolic dysfunction-associated steatotic liver disease
MCAD	medium-chain acyl-CoA dehydrogenase
MG	monoacylglycerol
MGDG	monogalactosyl diglyceride
MOS	mitochondrial oxidative stress
MTP	microsomal triglyceride transfer protein
MRM	multiple reaction monitoring
MLKL	mixed lineage kinase domain-like pseudokinase
MT	mutated type
NAFLD	non-alcoholic fatty liver disease
NF-κB	nuclear factor kappa B
OD	optical density
OE	overexpression
OPLS-DA	Orthogonal Partial Least Squares-Discriminant Analysis
PA	phosphatidic acid
PC	phosphatidylcholine
PC-O	phosphatidylcholine (ether bond)
PE	phosphatidylethanolamine
PE-O	phosphatidylethanolamine (ether bond)
PE-P	phosphatidylethanolamine (vinyl ether bond)
PERK	protein kinase R-like endoplasmic reticulum kinase
PG	phosphatidylglycerol
PMeOH	phosphatidymethanol
p-MLKL	phosphorylation of mixed lineage kinase domain-like pseudokinase
PS	phosphatidyl serine
Rluc	Renilla luciferase
ROS	reactive oxygen species
SHexCer	sulfatide-hexosyl ceramide
shRNA	small hairpin RNA
SM	sphingomyelins
SO	sodium oleate
SP	sodium palmitate
SPH	sphingosine
SREBP1	sterol regulatory element binding protein 1
SREBP1c	cleaved sterol regulatory element binding protein 1
TC	total cholesterol
TEM	transmission electron microscope
TG	triglyceride
TG-O	triglyceride (ether bond)
TM	tunicamycin
THA	thapsigargin
TUNEL	terminal deoxynucleotidyl transferase-mediated deoxyuridine triphosphate nick end labeling
UPR	unfolded protein response
μM	μmol/L
VIP	variable importance in projection
wk	week
WT	wildtype
XBP1	X box binding protein 1
Z-VAD	Z-Val-Ala-Asp(Ome)-fluoromethylketone

### Western blot analysis

Western blot analysis was conducted according to standard procedures using tissue lysates from the right hepatic lobe as described previously [90]. Briefly, tissues or hepatocytes were solubilized in radioimmunoprecipitation assay buffer containing phosphatase inhibitor (R0010, Solarbio, Beijing, China). After determining the protein concentration, 40 µg of each sample was separated by sodium dodecyl sulfate-polyacrylamide gel electrophoresis and transferred to polyvinylidene fluoride membranes (Millipore, USA). Subsequently, the membranes were blocked with 5% skim milk and incubated with primary antibodies (Table 3) overnight at 4 °C. Then, the membranes were incubated with horseradish peroxidase-conjugated anti-mouse (sc-516102, Santa Cruz, 1:10,000) or anti-rabbit (sc-2357, Santa Cruz; 1:10,000) antibodies overnight (at 4 °C) or for 5 h (at room temperature). The images were captured using a ChemiDoc^TM^ XRS^+^ imager (Bio-Rad) and quantified using the Quantum One software (Bio-Rad) and Image J software (NIH). Relative protein expression was reflected as the gray value of the proteins, and β-actin was used as the internal control.

**Table 3:** Primary antibody information.

Antibody	Source	Lot number	Manufacturer	Reactivity
ATF4	Rabbit mAb	11815	Cell Signaling Technology, Danvers, MA, USA	Mouse, human
β-actin	Mouse mAb	sc-81178	Santa Cruz, Dallas, TX, USA	Mouse, human
CHOP	Mouse mAb	ab11419	Abcam, Cambridge, MA, USA	Mouse, human
Cleaved caspase-3	Rabbit mAb	9664	Cell Signaling Technology, Danvers, MA, USA	Human, mouse
F4/80	Mouse mAb	sc-365340	Santa Cruz	Human, mouse
GRP78	Rabbit mAb	ab108615	Abcam, Cambridge, MA, USA	Human, mouse
GPX4	Mouse mAb	sc-166437	Santa Cruz	Mouse, human
HRD1	Rabbit pAb	PA5-76137	Thermo Fisher Scientific, Waltham, MA, USA	Mouse, human
HNF1α	Mouse mAb	sc-393668	Santa Cruz	Mouse, human
MCAD	Mouse mAb	sc-365109	Santa Cruz	Mouse, human
MLKL	Rabbit mAb	PA5-34733	Thermo Fisher Scientific, Waltham, MA, USA	Mouse, human
MTP	Mouse mAb	sc-515742	Santa Cruz	Mouse, human
p-MLKL	Rabbit mAb	37333S	Cell Signaling Technology, Danvers, MA, USA	Mouse, human
RelA	Mouse mAb	sc-514451	Santa Cruz	Mouse, human
SREBP1	Mouse mAb	MA5-16124	Thermo Fisher Scientific, Waltham, MA, USA	Mouse, human

*mAb* monoclonal antibody, *pAb* polyclonal antibody.

### Transmission electron microscopy (TEM)

Many 1 mm^3^ tissue samples were prepared from freshly obtained mouse liver. The tissues were fixed in 3% glutaraldehyde (4 °C, 4 h), dehydrated in ethanol and acetone, and embedded in epoxy resin. Subsequently, ultrathin sections (70 nm) were generated and stained in uranyl acetate (room temperature, 30 min) and lead citrate (room temperature, 10 min), respectively. Finally, the ultrastructure of the liver is observed using a transmission electron microscope (JEM-1400, JOEL, Tokyo, Japan).

### Cells and treatment

Human hepatocyte cell lines (HepG2 and LO2) were obtained from the American Type Culture Collection and cultured in RPMI-1640 medium enriched with 10% fetal bovine serum and 1% penicillin/streptomycin. To induce steatosis in vitro, hepatocytes underwent treatment with sodium oleate (SO) and sodium palmitate (SP) (KC006, Kunchuang, China) for 48 h. The cells were divided into three groups based on their treatment regimen: an SO/SP group (treated with varying concentrations of SO/SP: 125/62.5, 250/125, and 500/250 μM), a solvent control group (treated with bovine serum albumin (BSA)), and a normal group (received no treatment).

Plasmids for RelA overexpression (OE) and shRNAs targeting RelA and/or HNF1α (Genechem, China) were transfected into hepatocytes using ExFect Transfection Reagent (T101-01, Vazyme, Nanjing, China), following the manufacturer’s instructions. As controls, cells were transfected with either an empty plasmid or non-targeting control shRNA. The sequences of the shRNAs used are listed in Table 4.

**Table 4:** shRNA sequences used in vitro.

Parameter		5ʹ to 3ʹ
*RELA*	*RELA* shRNA	CACCATCAACTATGATGAGTTCGAAAACTCATCATAGTTGATGGTG
	Control shRNA	AAACGTGACACGTTCGGAGAACGAATTCTCCGAACGTGTCACGTTT
*HNF1A*	*HNF1A* shRNA	GCTAGTGGAGGAGTGCAATAGCGAACTATTGCACTCCTCCACTAGC
	Control shRNA	AAACGTGACACGTTCGGAGAACGAATTCTCCGAACGTGTCACGTTT

HepG2 cells with knockouts (KO) for *RELA* or *HNF1A* were custom-engineered by OBiO Technology (Shanghai, China). Wild-type (WT) cells served as controls. The cell groups included *RELA* or *HNF1A*-WT, *RELA* or *HNF1A*-KO, and both WT and KO cells treated with SO/SP. The sequences for the single-guide RNAs were as follows: *RELA*: 5’-GCTTCCGCTACAAGTGCGAGGGG-3’ and *HNF1A*: 5’-GCGGACGTACCAGGTGTACA-3ʹ.

To investigate the roles of apoptosis and necroptosis in steatotic hepatocytes, these model cells were treated with specific inhibitors. To inhibit necroptosis, hepatocytes were treated with necrostatin-1 (Sigma, USA), a necroptosis inhibitor preventing the MLKL phosphorylation by inhibiting the activation of receptor-interacting protein kinase-1 and -3 [91, 92], for the last 24 h of a 48-h treatment period. For apoptosis inhibition, Z-Val-Ala-Asp(Ome)-fluoromethylketone (Z-VAD; Sigma, USA), a pan-caspase inhibitor [93], was used to impede apoptosis for the last 24 h of a 48-h treatment period. DMSO was used as the solvent control. The treatment groups were: control group (treated with BSA for 48 h, with DMSO added for the last 24 h), necrostatin-1 or Z-VAD group (treated with BSA for 48 h, with 20 μM necrostatin-1 or 30 μM Z-VAD added for the last 24 h), SO/SP group (treated with SO/SP for 48 h, with DMSO added for the last 24 h), necrostatin-1 or Z-VAD + SO/SP group (treated with SO/SP for 48 h, with necrostatin-1 or Z-VAD added for the last 24 h).

To evaluate the effect of ER stress on lipid metabolism, hepatocytes were treated with 0.5 μM thapsigargin (THA, Sigma, USA) for 0, 12, 24, or 48 h, with DMSO as the solvent control. THA disrupts calcium homeostasis by inhibiting the ER/sarcoplasmic Ca^2+^-ATPase, leading to ER stress [94].

To assess the role of SREBP1-mediated lipogenesis in hepatocyte steatosis, HepG2 cells were treated with fatostatin (Sigma, USA), a SREBP1 inhibitor [95], during the treatment duration. DMSO served as the solvent control. The experiment groups included the SO/SP group (treated with 250/125 μM SO/SP, and DMSO for 48 h), fatostatin + SO/SP group (treated with 250/125 μM SO/SP, and 20 μM fatostatin for 48 h).

### Oil Red O staining

Lipid staining was performed using the Oil Red O Staining Kit (G1262, Solarbio), adhering to the manufacturer’s instructions. The stained cells were imaged using a light microscope (DXS-3; Shanghai Telon Optical Instruments, Shanghai, China).

### Measurement of TG content

TG content in hepatocytes, cell culture supernatant, and liver tissue was determined using the TG Content Assay kit (AKFA003M, Boxbio, China), adhering to the instructions provided with the kit. Measurements were conducted using a BioTek Epoch microplate reader (Winooski, Vermont, USA) at a wavelength of 420 nm. The TG content was then calculated based on the data obtained.

### Measurement of malondialdehyde (MDA) levels

The MDA levels in mouse liver and hepatocytes in vitro were assessed using a thiobarbituric acid test kit (A003; Nanjing Jiancheng, China), following the manufacturer’s guidelines. Measurements of optical density (OD), also known as absorbance, were performed at 532 nm using a microplate reader (BioTek Epoch). The MDA content in the liver tissue and hepatocytes was calculated using the instructions provided with the assay kit.

### Detection of ROS production

The ROS production was detected using the fluorescent probe 2ʹ, 7′-dichlorodihydrofluorescein diacetate (DCFH-DA) (AKCE002-1; Boxbio). LO2/HepG2 cells were incubated with DCFH-DA under indicated conditions according to the manufacturer’s instructions. ROS production was assayed using a fluorescence microscope with excitation and emission wavelengths of 480 nm and 525 nm, respectively. The intensity of green fluorescence reflects the content of ROS.

### Reduced glutathione (GSH) and oxidized glutathione (GSSG) determination

The levels of GSH and GSSG contents in liver samples or hepatocytes in vitro were measured using the commercially available kits (GSH, AKPR008M; GSSG, AKPR009M; Boxbio) according to the manufacturer’s instructions. The OD of GSH and GSSG at 412 nm was measured using a microplate reader (BioTek Epoch). The GSH and GSSG contents in mouse liver samples or hepatocytes in vitro were calculated using the instructions provided with the assay kit.

### Mitochondrial ETC complex I (CI) and complex III (CIII) assay

The activities of mitochondrial CI and CIII in liver samples were determined using the Mitochondrial Complex I assay kit (ADS-W-FM006, Adsbio, China) and the Mitochondrial Complex III assay kit (ADS-W-X012, Adsbio, China) by measuring absorbance at 340 nm and 550 nm, respectively (BioTek Epoch). Sample preparation, assays, absorbance readings using an enzyme analyzer, and calculations of enzymatic activities were performed according to the respective kit manuals.

### Acridine Orange staining

Lysosomal membrane permeabilization (LMP) in hepatocytes was assessed using the Acridine Orange staining Kit (CA1143, Solarbio, Beijing, China), following the manufacturer’s protocol. Acridine Orange emits red fluorescence at high concentrations within intact lysosomes and green fluorescence at lower concentrations in the cytoplasm and nucleus. The stability of the lysosomal membrane was evaluated by observing changes in the red and green fluorescence using a fluorescence microscope [96].

### Cell viability assay

To evaluate hepatocyte viability, a Cell Counting Kit-8 (CCK-8, A311, Vazyme, Nanjing, China) was used following a standard protocol. In brief, 5000 hepatocytes (HepG2 or LO2 cells) under indicated experimental conditions were seeded into 96-well plates (5 replicates per sample). The same amount of culture medium was added into each well for blank controls. At each testing point, the CCK-8 reagent was added into each well and incubated for 1 h at 37 °C. Subsequently, OD at 450 nm was measured with a microplate reader (BioTek Epoch) [97].

### Double luciferase reporter gene assay

The promoter sequence of the target gene was obtained from the UCSC genome browser (https://genome.ucsc.edu/), and JASPAR (https://jaspar.genereg.net/) was utilized to predict the binding site of RelA or HNF1α to the target gene promoter region. The target gene promoter region containing the WT or mutated type (MT) predicted RelA or HNF1α binding site was engineered into reporter plasmids expressing firefly luciferase (Fluc). The open reading frame of RelA or HNF1α was cloned into a pcDNA3.1 (pcDNA3.1-RelA) plasmid, and an empty vector was used as the control. HEK293FT cells were co-transfected with the internal reference reporter plasmid (Renilla luciferase, Rluc), reporter plasmid containing WT or MT target gene promoter region, and RelA or HNF1α expressing plasmid (or empty vector). Subsequently, the cells were lysed and subjected to a luciferase activity assay using a commercially available dual-luciferase reporter assay system (Beyotime, China), as previously reported [98]. The degree of target reporter activation was determined by comparing the ratios of Fluc activity to Rluc activity (Fluc/Rluc), and the resulting ratios were later compared to determine the degree of reporter activation.

### Flow cytometry-based cell apoptosis analysis

The Annexin V-allophycocyanin (APC)/propidium iodide (PI) cell apoptosis detection kit (Procell, China) was used to determine cell apoptosis according to the manufacturer’s instructions [97]. Briefly, single-cell suspensions of hepatocytes were obtained by trypsinization, washing, and pelleting. Next, 100 µl of suspended cells were added to a flow tube with 500 μl of binding buffer containing 5 µl of Annexin V-APC and 5 µl of PI and incubated for 15 min in the dark. The samples were then analyzed using flow cytometry (BriCyte E6, Mindray, China). This experiment was repeated three times, and the apoptotic index was calculated as the ratio of Annexin V-positive cells to the total cell count.

### Statistical analysis

Statistical analyses were performed using SPSS version 29.0 (IBM, USA). Continuous variables that followed a normal distribution were evaluated using the one-sample Kolmogorov-Smirnov test, with their means and standard deviations reported. To compare the two groups, an independent *t*-test was used. For assessing statistical significance among multiple groups, one-way or two-way variance analysis (ANOVA) was employed. In cases where significant differences were identified, post hoc pairwise comparisons were conducted using the least significant difference t-test method. A *P*-value of less than 0.05 was considered statistically significant.

## Supplementary information

Supplementary Materials

## Data Availability

The datasets generated and analyzed during the current study are available from the corresponding author upon reasonable request.
