# Supplementary material for: Impaired RelA signaling and lipid metabolism dysregulation in hepatocytes: driving forces in the progression of metabolic dysfunction-associated steatotic liver disease
Source: Cell Death Discov. 2025 Feb 5;11:49. doi: 10.1038/s41420-025-02312-3 (PMC11799324; doi:10.1038/s41420-025-02312-3)

Impaired RelA Signaling in Hepatocytes promotes the Progression of Metabolic Dysfunction-associated Steatotic Liver Disease by Remodeling Lipid Metabolism

Running title: Impaired RelA signaling promotes MASLD

Yihuai He[1,2], Jinlian Jiang[2], Lili Ou[2], Yunfen Chen[2], Aikedaimu Abudukeremu[1], Guimei Chen[2], Weiwei Zhong[3], Zhigang Jiang[4], Nuerbiye Nuermaimaiti[1], Yaqun Guan[1,5*]

[1]State Key Laboratory of Pathogenesis, Prevention and Treatment of High Incidence Diseases in Central Asia, Xinjiang Key Laboratory of Molecular Biology for Endemic Diseases, Department of Pathology, School of Basic Medical Sciences, Xinjiang Medical University, Urumqi 830000, Xinjiang, China

[2]Department of Infectious Diseases, Affiliated Hospital of Zunyi Medical University, Zunyi 563003, Guizhou, China

[3]Department of Infectious Diseases, Jingmen Central Hospital, Jingmen 448000, Hubei, China

[4]School of Public Health, Zunyi Medical University, Zunyi 563099, Guizhou, China

[5]Xinjiang Second Medical College, Karamay 834000, China

***Corresponding author**: Yaqun Guan

State Key Laboratory of Pathogenesis, Prevention and Treatment of High Incidence Diseases in Central Asia, Xinjiang Key Laboratory of Molecular Biology for Endemic Diseases, Department of Pathology, School of Basic Medical Sciences, Xinjiang

Medical University, Urumqi 830000, Xinjiang, China

E-mail: yaqunguan557@xjmu.edu.cn

Supplementary Figures

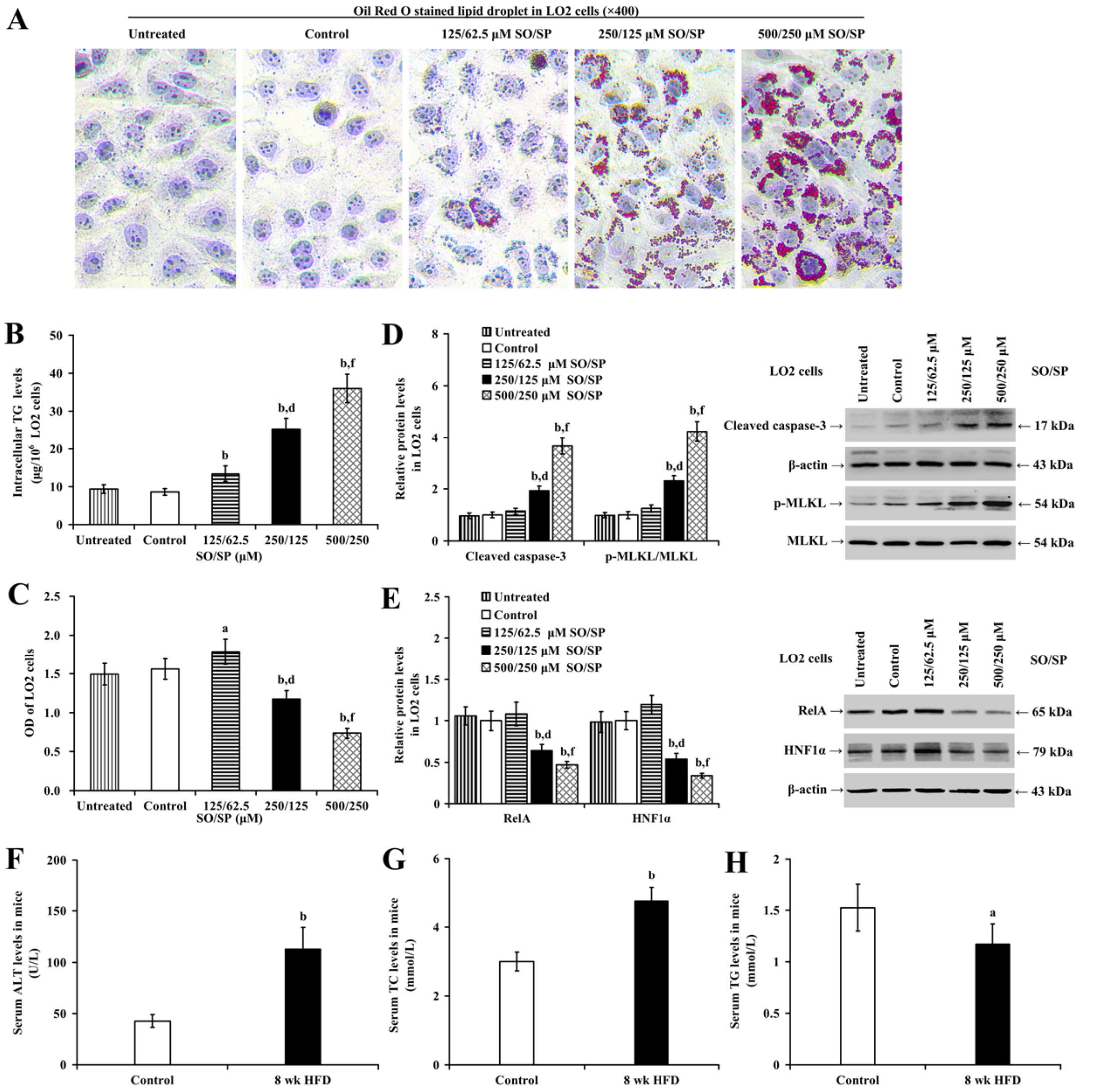

Supplementary Fig. 1. Steatotic hepatocytes exhibit reduced levels of RelA and HNF1α in MASLD. (A–E) LO2 cells were treated with SO/SP, vehicle or left untreated at indicated concentrations to induce steatosis. Under this condition, (A) lipid droplet accumulation, (B) intracellular TG levels, (C) cell viability and the

expression of (D) cleaved caspase-3, p-MLKL, and (E) RelA and HNF1α were evaluated. (F–H) Comparisons of serum (F) ALT, (G) TC, and (H) TG levels were evaluated and analyzed in mice (n=12) under indicated conditions. a *P*<0.05, b *P*<0.01, vs. control group; d *P*<0.01, vs. SO/SP group (125/62.5 μM); f *P*<0.01, vs. SO/SP group (250/125 μM). HFD: high-fat diet; SO/SP: sodium oleate and sodium palmitate; μM: μmol/L; wk: week.

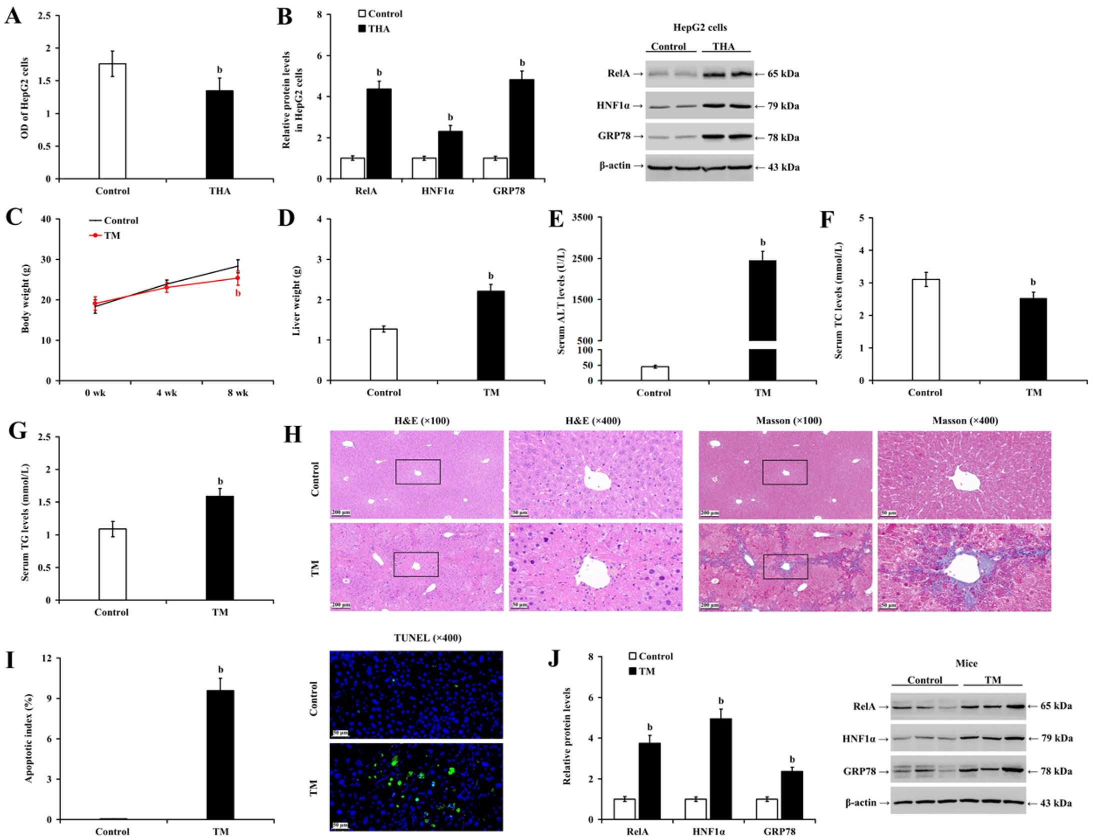

Supplementary Fig. 2. Hepatocytes without steatosis exhibit elevated RelA and HNF1α expression under ER stress. (A and B) ER stress was induced in HepG2 cells using THA (or vehicle), and (A) cell viability and (B) the expression of RelA, HNF1α, and GRP78 were evaluated. (C–K) Mice (n=12) were treated with TM or vehicle (DMSO) for 8 weeks. Under this condition, their (C) body weights, (D) liver

weights, serum levels of (E) ALT, (F) TC, and (G) TG were determined and compared. (H) Representative images of H&E (left panels), and Masson staining (right panels) of mouse livers. (I) Hepatic apoptotic index (calculated from the results of TUNEL staining). (J) Analyzed data (left panel) and representative images (right panel) of Western blot analysis showing the expression of RelA, HNF1α, and GRP78 in mouse livers under different conditions. b $P<0.01$, vs. control group. THA: thapsigargin; TM: tunicamycin; wk: week.

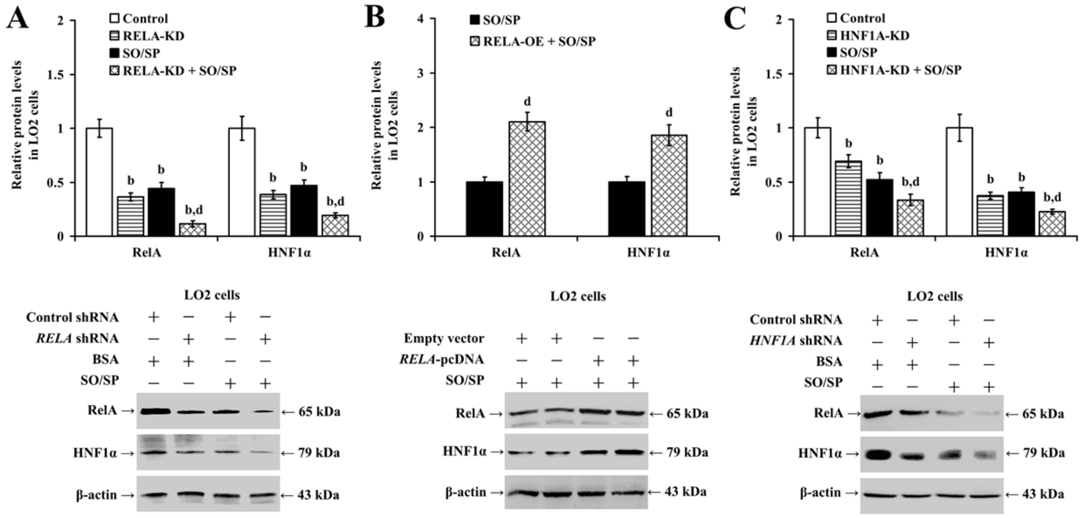

Supplementary Fig. 3. Lack of mutual transcriptional activation between RelA and HNF1α during hepatocyte steatosis. (A–C) Quantified results (upper panels) and representative images (lower panels) of western blot analysis showing the expression of RelA and HNF1α in LO2 cells under indicated conditions. b $P<0.01$, vs. control group; d $P<0.01$, vs. SO/SP group. BSA: bovine serum albumin; KD: knockdown; OE: overexpression; SO/SP: sodium oleate and sodium palmitate.

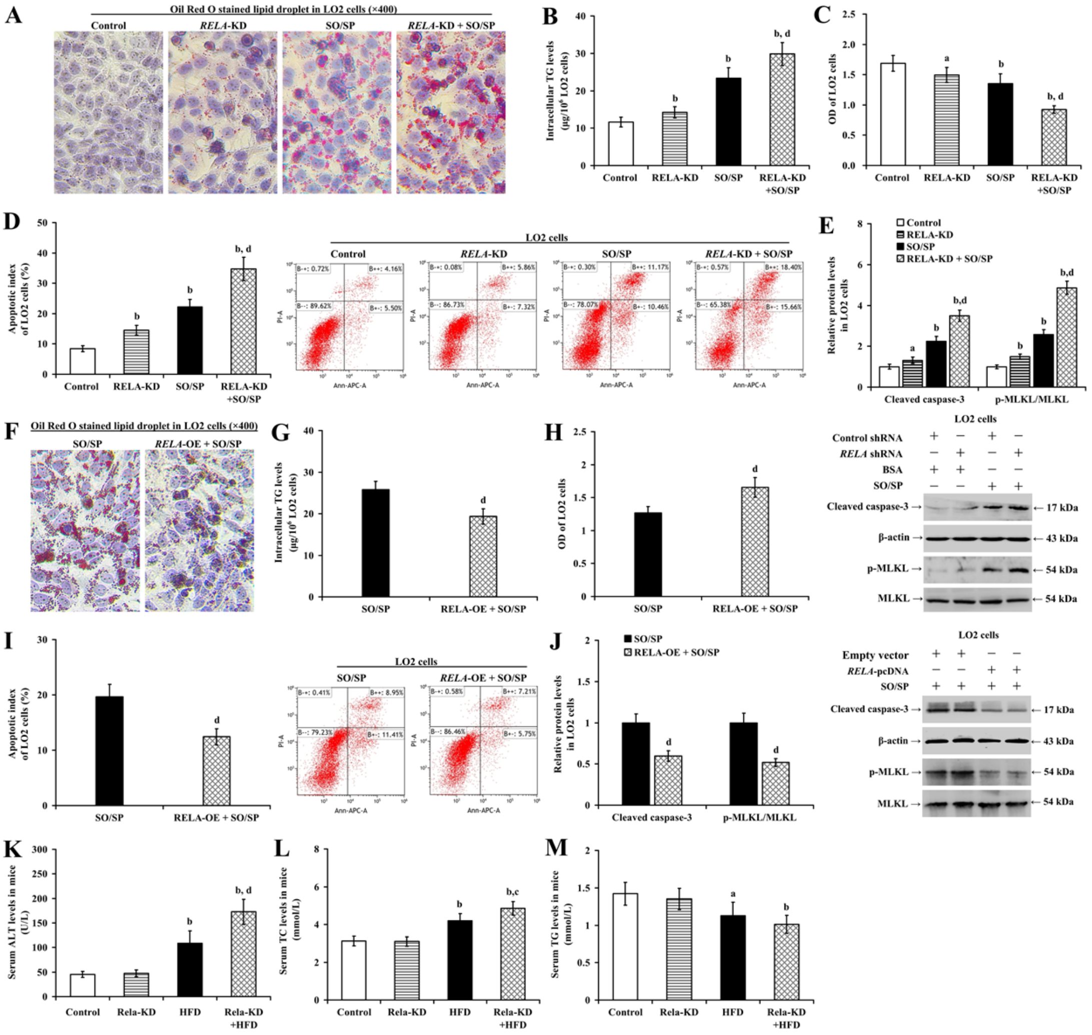

Supplementary Fig. 4. RelA mitigates hepatocyte steatosis. (A–E) LO2 cells with or without *RELA*-KD were treated with or without SO/SP. Under this condition, (A) lipid droplet accumulation, (B) intracellular TG levels, (C) cell viability, (D) cell apoptosis, and (E) the expression of cleaved caspase-3 and p-MLKL were determined and compared. (F–J) LO2 cells with or without *RELA*-OE were treated with SO/SP, and (F) lipid droplet accumulation, (G) intracellular TG levels, (H) cell viability, (I) cell apoptosis, and (J) the expression of cleaved caspase-3 and p-MLKL were determined and analyzed. (K–M) Analyzed data presenting the serum levels of (K) ALT, (L) TC, and (M) TG in mice with or without *RELA*-KD fed a normal diet or

HFD. a $P<0.05$, b $P<0.01$, vs. control group; c $P<0.05$, d $P<0.01$, vs. SO/SP group or HFD group. HFD: high-fat diet; KD: knockdown; OE: overexpression; SO/SP: sodium oleate and sodium palmitate.

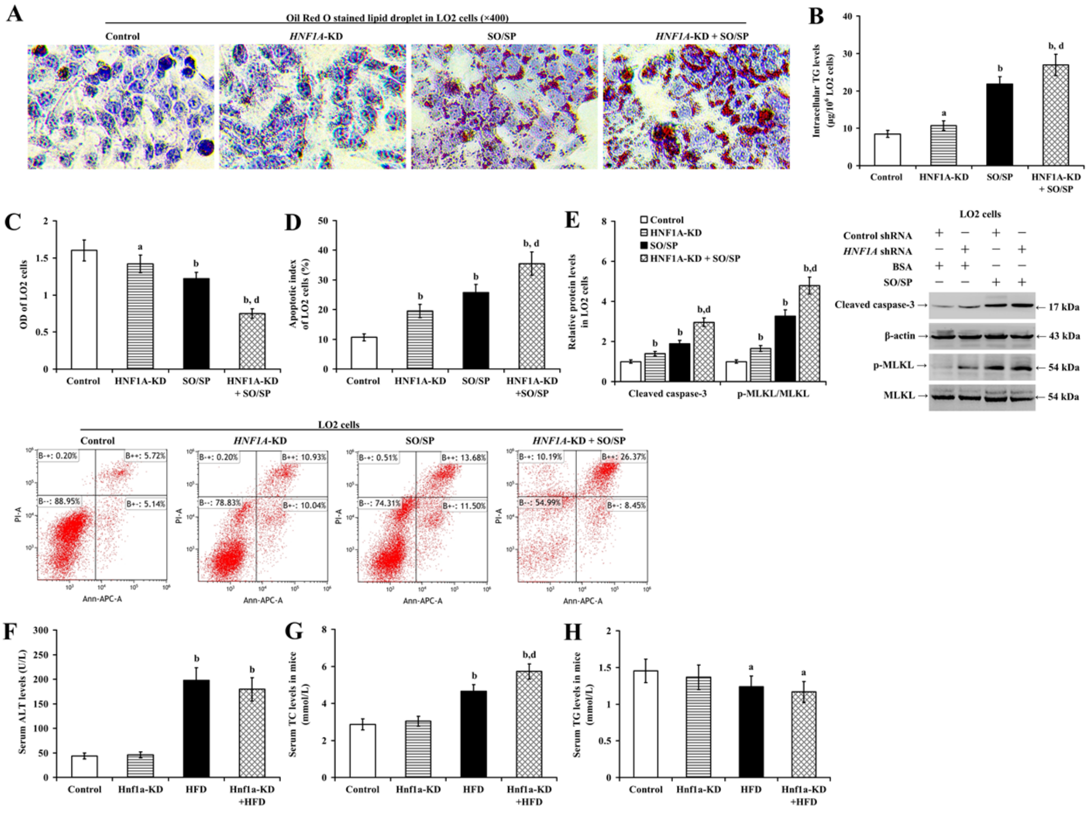

Supplementary Fig. 5. *HNF1A* knockdown exacerbates hepatocyte steatosis. (A–E) LO2 cells with or without *HNF1A*-KD were treated with SO/SP. Under this condition, (A) intracellular lipid droplet accumulation, (B) TG content, (C) cell viability, (D) cell apoptosis, and (E) the expression of cleaved caspase-3 and p-MLKL were determined and compared. (F–H) Mice (n=12) with or without *Hnf1a* KD were fed with normal diet or HFD. Their serum levels of (F) ALT, (G) TC, and (H) TG were evaluated and analyzed. a $P<0.05$, b $P<0.01$, vs. control group; d $P<0.01$, vs. SO/SP group or HFD group. BSA: albumin; KD: knockdown; HFD: high-fat diet; KD:

knockdown; SO/SP: sodium oleate and sodium palmitate.

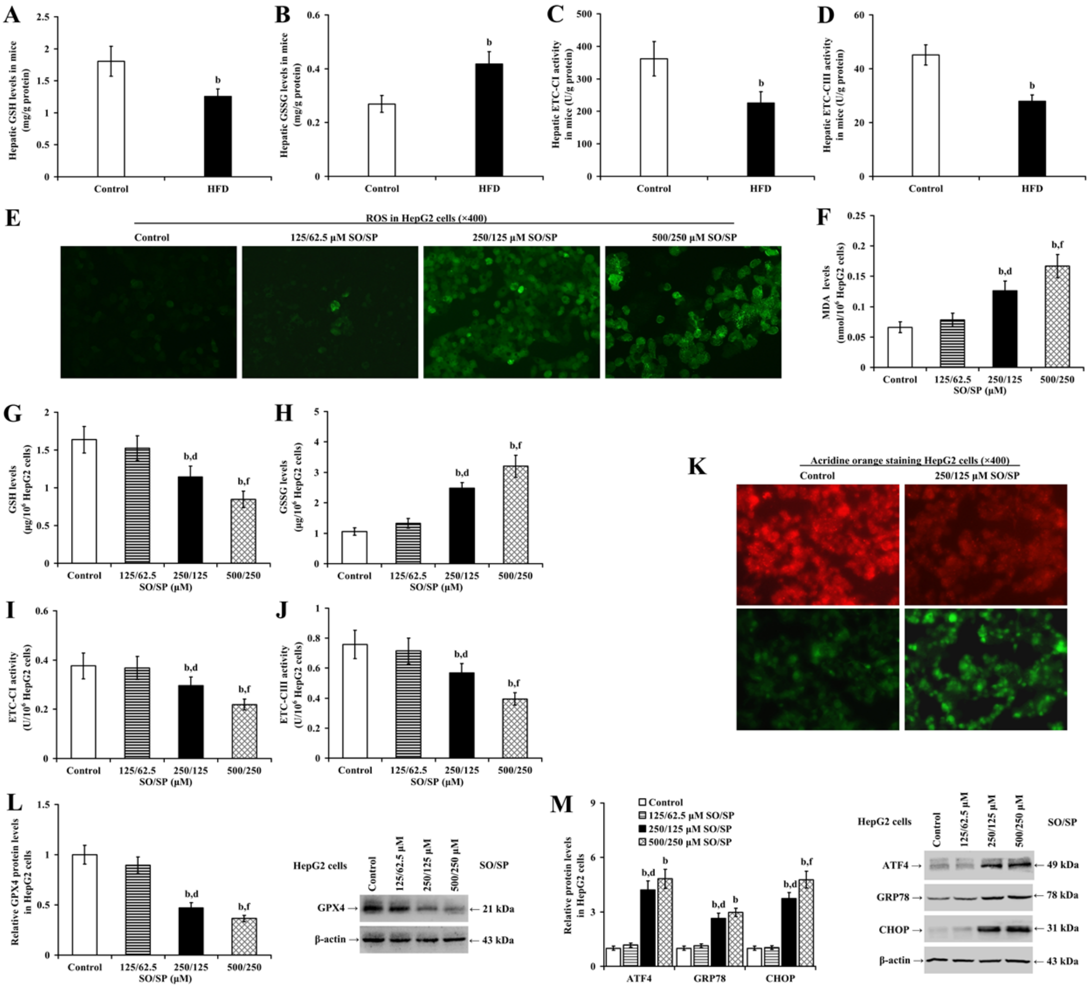

Supplementary Fig. 6. Lipid accumulation in hepatocytes exhibits lipotoxic features, potentially triggering apoptosis and necroptosis. (A–D) Analyzed data detailing the changes in (A) GSH levels, (B) GSSG levels, (C) ETC-CI activity, and (D) ETC-CIII activity in the livers of mice fed a normal diet or HFD. (E–M) HepG2 cells were treated with SO/SP at the indicated concentrations. Under this condition, (E) ROS levels, (F) MDA levels, (G) GSH levels, (H) GSSG levels, (I) ETC-CI activity, (J) ETC-CIII activity, and (K) changes in LMP (the reduction in red fluorescence and the increase in green fluorescence signify the strengthening of LMP) were determined

in the cells. (L and M) Quantified results and representative images of western blotting demonstrating the expression of (L) GPX4, (M) ATF4, GRP78, and CHOP in HepG2 cells under indicated conditions. b P<0.01, vs. control group; d P<0.01, vs. SO/SP group (125/62.5 μM); f P<0.01, vs. SO/SP group (250/125 μM). HFD: high-fat diet; LMP: lysosomal membrane permeabilization; SO/SP: sodium oleate and sodium palmitate; μM: μmol/L.

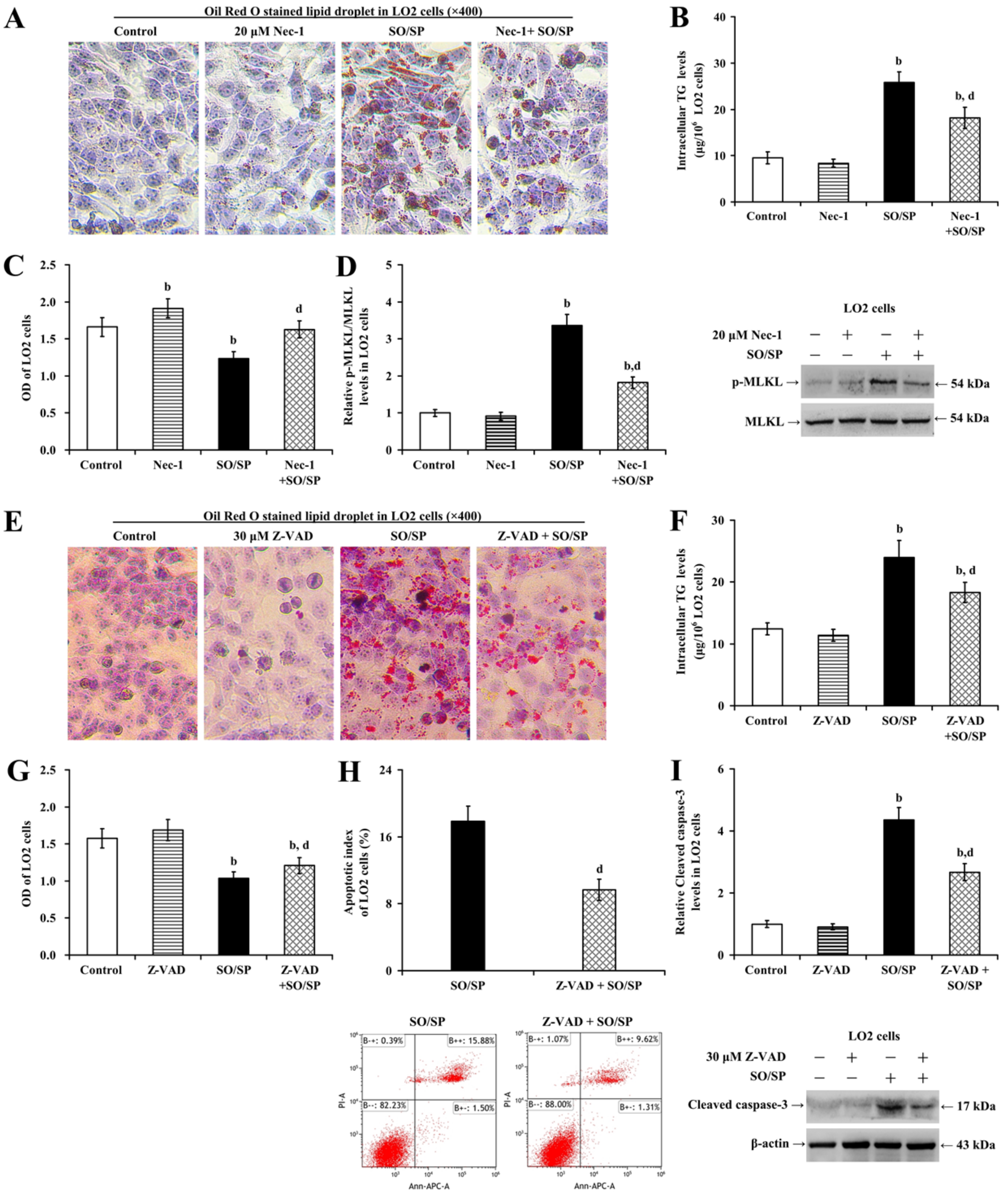

Supplementary Fig. 7. Necrostatin-1 and Z-VAD alleviate SO/SP-induced hepatocyte injury. (A–D) LO2 cells were treated with or without SO/SP in the presence or absence of Nec-1 (20 μM). Under this condition, (A) lipid droplet accumulation, (B) intracellular TG levels, (C) cell viability, and (D) p-MLKL expression were determined. (E–I) LO2 cells were treated with or without SO/SP in the presence or absence of Z-VAD (30 μM). Under this condition, (E) lipid droplet

accumulation, (F) intracellular TG levels, (G) cell viability, (H) cell apoptosis, and (I) cleaved caspase-3 expression were determined. b P<0.01, vs. control group; d P<0.01, vs. SO/SP group. Nec-1: necrostatin-1; SO/SP: sodium oleate and sodium palmitate; μM: μmol/L.

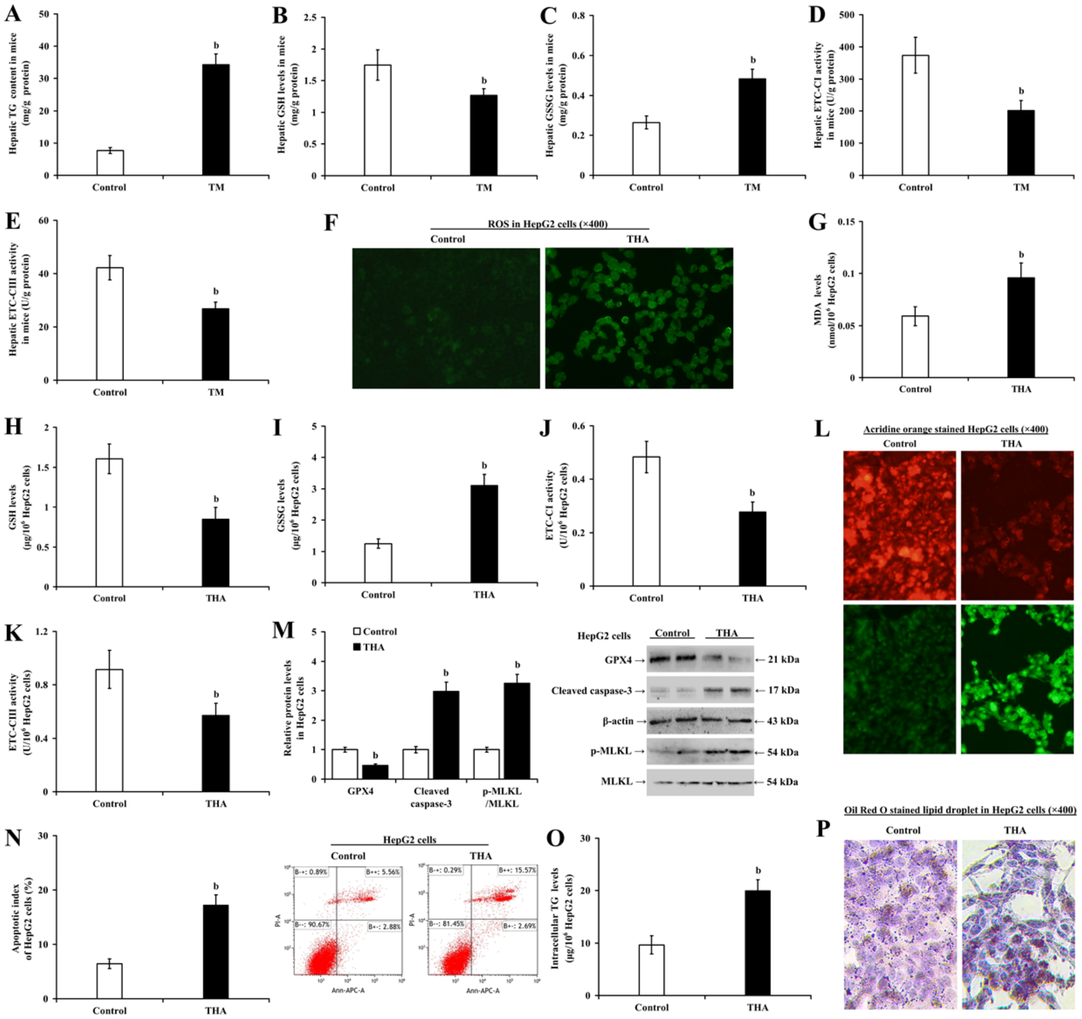

Supplementary Fig. 8. ER stress-initiated hepatotoxicity mirrors the processes involved in hepatocyte steatosis. (A–E) The mice were treated with or without TM, and the changes in (A) hepatic TG content, (B) GSH levels, (C) GSSG levels, (D) ETC-CI activity, and (E) ETC-CIII activity were measured. (F–P) In THA-treated

HepG2 cells, the changes in (F) ROS levels (green fluorescence intensity), (G) MDA levels, (H) GSH levels, (I) GSSG levels, (J) ETC-CI activity, (K) ETC-CIII activity, (L) LMP were evaluated. The expression of (M) GPX4, cleaved caspase-3, and p-MLKL, (N) cell apoptosis, (O) intracellular TG levels, and (P) lipid droplet accumulation were also determined in HepG2 cells treated with or without THA. b $P<0.01$, vs. control group. LMP: lysosomal membrane permeabilization; THA: thapsigargin; TM: tunicamycin.

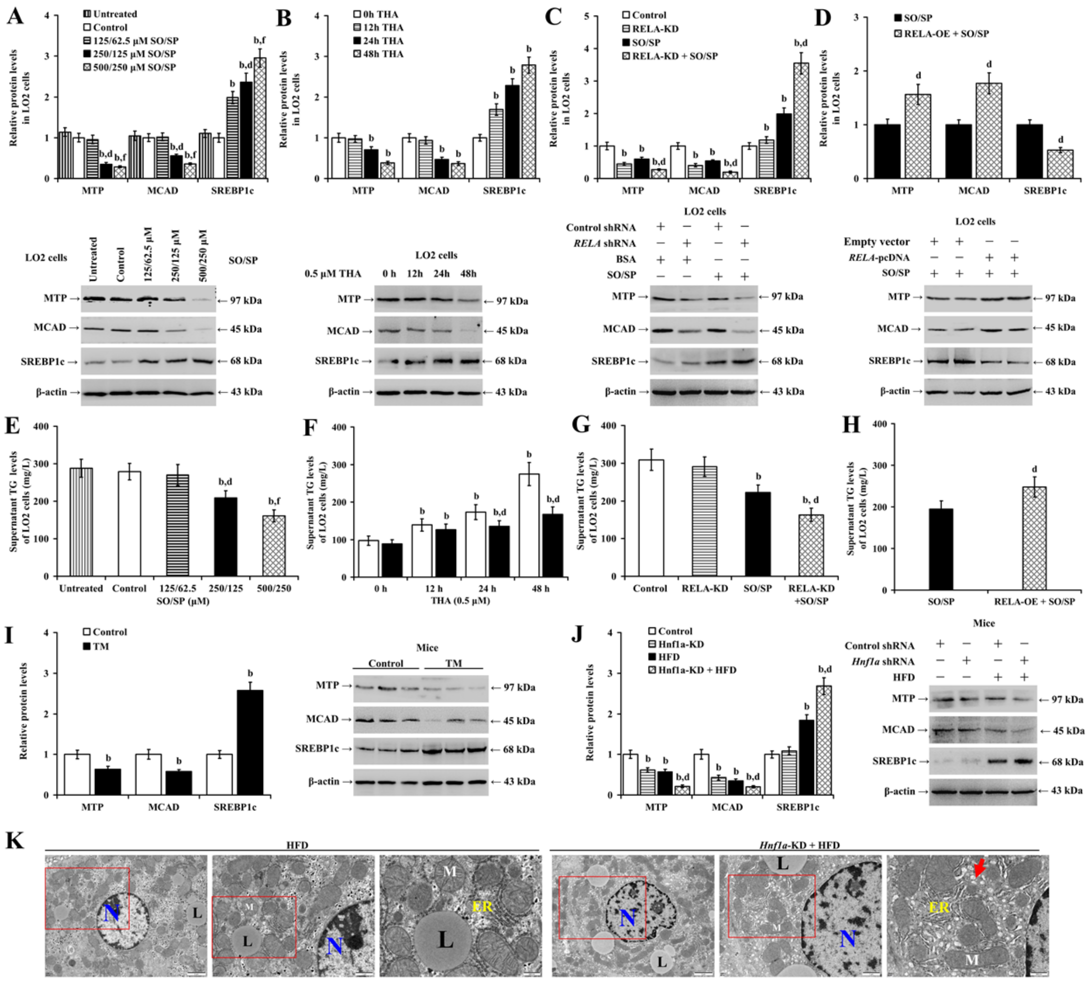

Supplementary Fig. 9. Impaired RelA signaling reprograms lipid metabolism promotes lipid accumulation in hepatocytes with steatosis. (A–D) The expression

of lipid metabolism-related proteins (MTP, MCAD, SREBP1c) in LO2 cells under the conditions of (A) SO/SP treatment, (B) THA treatment (0.5 μM), and SO/SP treatment with or without (C) *RELA*-KO or (D) *RELA*-OE. (E-H) Extracellular TG levels were measured under the conditions of (E) SO/SP treatment, (F) THA treatment, and SO/SP treatment with or without (G) *RELA*-KO or (H) *RELA*-OE. (I) Analyzed data and representative images of Western blot analysis showing the expression of lipid metabolism-related proteins in the livers from mice induced with or without TM. (J) Analyzed data and representative images of Western blot analysis showing the expression of lipid metabolism-related proteins in the livers from mice with or without *Hnf1a*-KD under normal diet or HFD. (K) Representative TEM images showing the ultrastructure of mouse liver under *Hnf1a*-KD. Red rectangles indicate magnified area. b $P<0.01$, vs. conrol group or 0 h group; d $P<0.01$, vs. SO/SP group (125/62.5 μM), the corresponding SO/SP group, or HFD group; f $P<0.01$, vs. SO/SP group (250/125 μM). BSA: bovine serum albumin; HFD: high-fat diet; KD: knockdown; OE: overexpression; SO/SP: sodium oleate and sodium palmitate; THA: thapsigargin; TM: tunicamycin; μM: μmol/L.

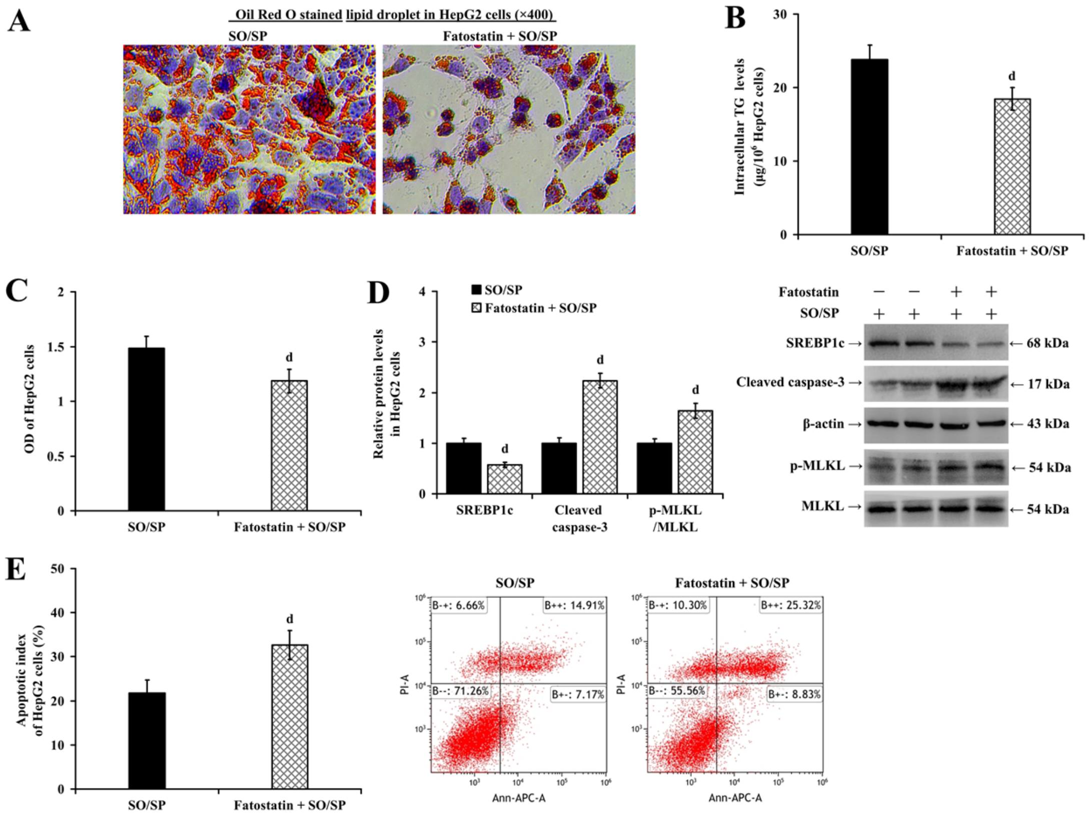

Supplementary Fig. 10. Fatostatin reduces SO/SP-induced lipid accumulation and exacerbates cell injury in HepG2 cells. (A–E) HepG2 cells were treated with SO/SP in the presence or absence of fatostatin (20 μM). Under this condition, (A) lipid droplet accumulation, (B) intracellular TG levels, (C) cell viability, (D) the expression of SREBP1c, cleaved caspase-3, and p-MLKL, and (E) cell apoptosis were evaluated. d *P*<0.01, vs. SO/SP group. SO/SP: sodium oleate and sodium palmitate; μM: μmol/L.

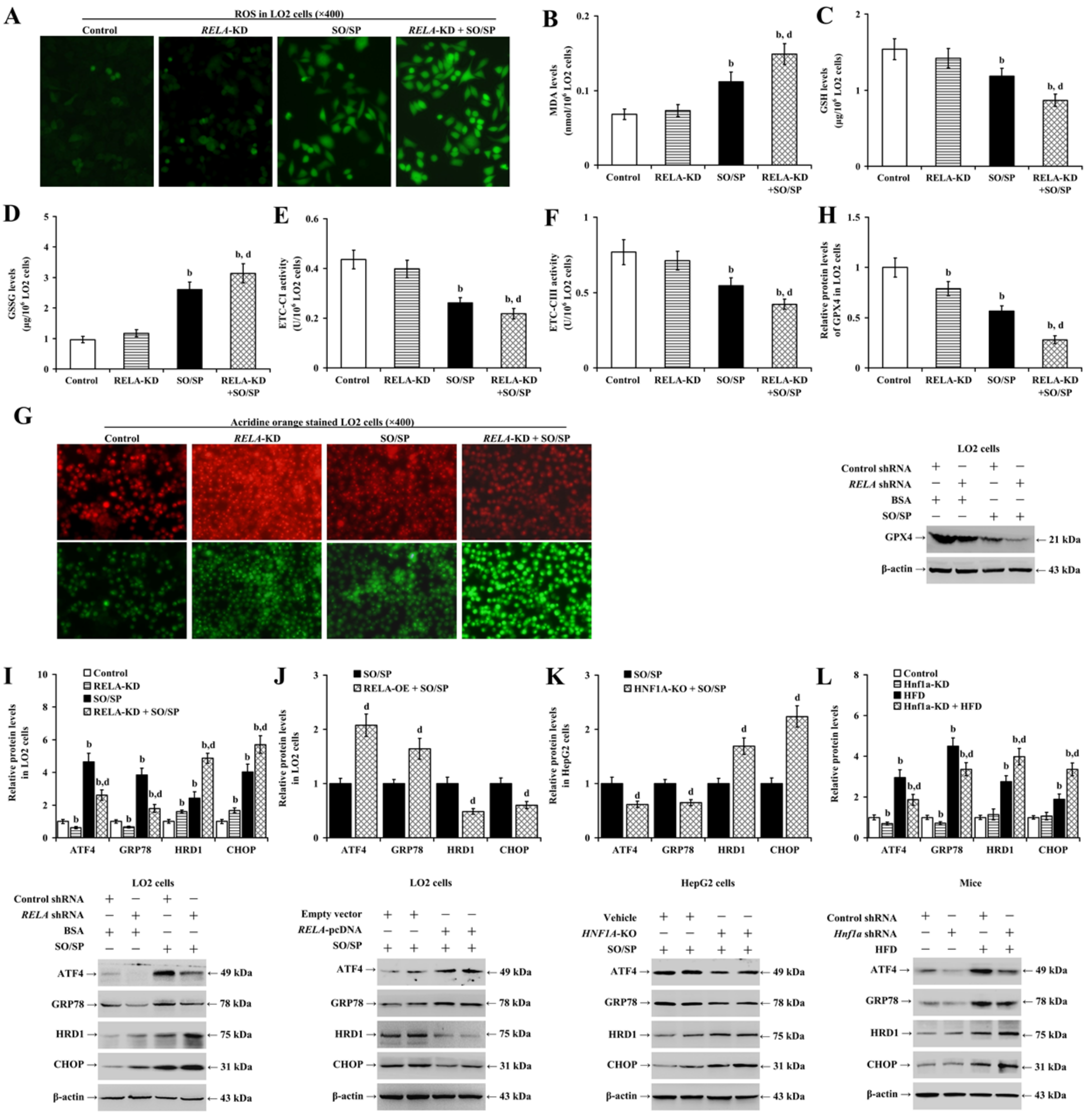

Supplementary Fig. 11. Impaired RelA signaling remodels the ER stress response and exacerbates lipotoxicity during hepatocyte steatosis. (A–H) LO2 cells with or without *RELA*-KD were treated with or without SO/SP. Under this condition, (A) ROS levels, (B) MDA levels, (C) GSH levels, (D) GSSG levels, (E) ETC-CI activity, (F) ETC-CIII activity, (G) LMP status, and (H) GPX4 expression were evaluated. (I–K) Analyzed data and representative images of western blot analysis showing the expression of ER stress-related proteins in LO2 cells treated with vehicle or SO/SP with or without (I) *RELA*-KD or (J) *RELA*-OE. (K)

Representative western blot images presenting the expression of ER stress-related proteins in HepG2 cells under the indicated conditions. (L) Analyzed data and representative images of Western blot analysis showing the expression of ER stress-related proteins in normal diet or HFD-fed mice with or without *Hnf1a*-KD. b $P<0.01$, vs. control group; d $P<0.01$, vs. SO/SP group or HFD group. BSA: bovine serum albumin; HFD: high-fat diet; SO/SP: sodium oleate and sodium palmitate.

Fig. 1D

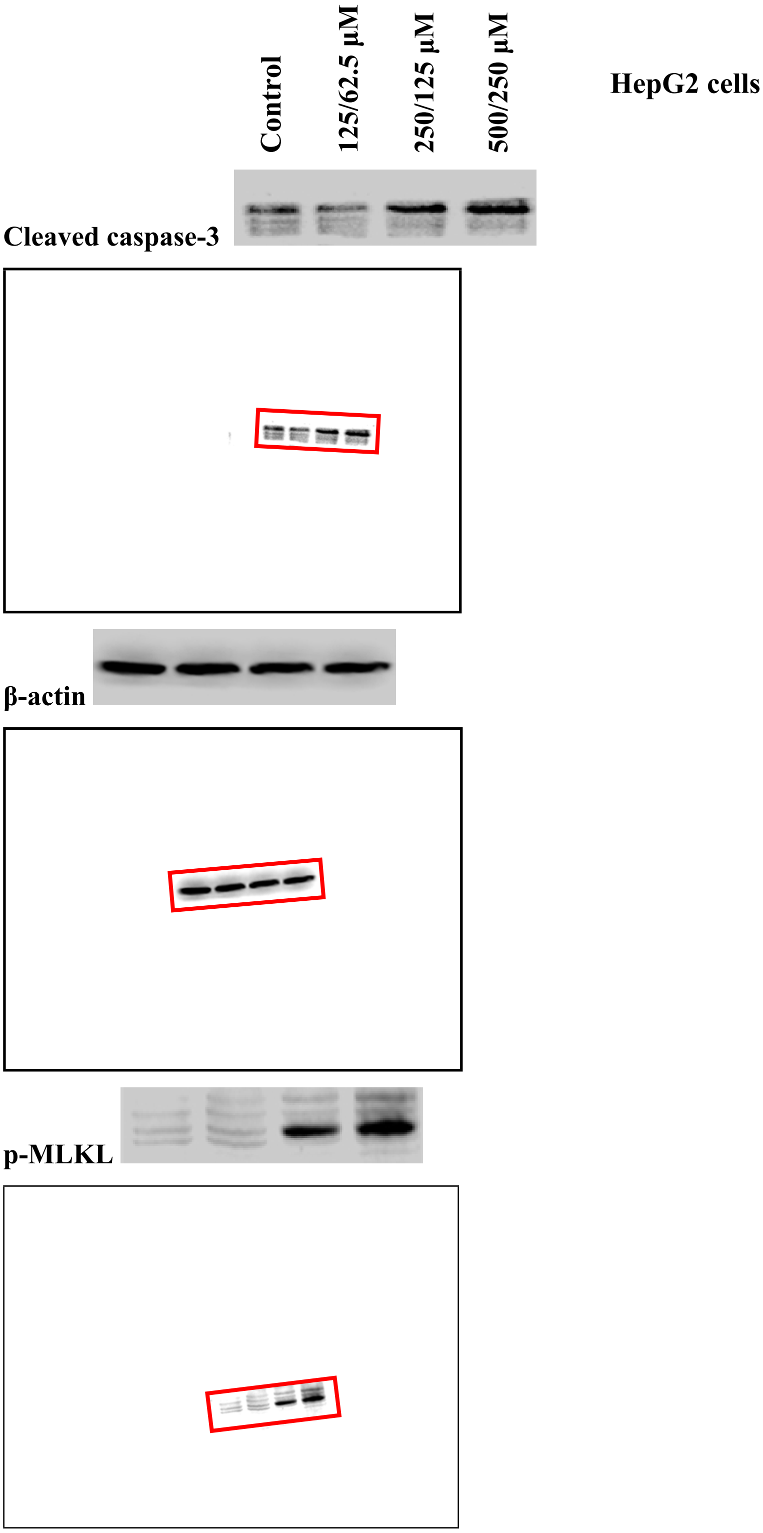
Control
125/62.5 µM
250/125 µM
500/250 µM
HepG2 cells
Cleaved caspase-3
β-actin
p-MLKL

MLKL

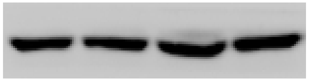

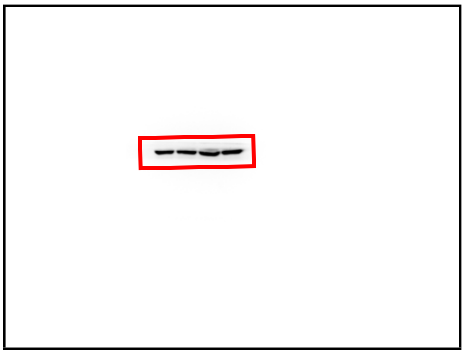

Fig. 1E

Control | 125/62.5 µM | 250/125 µM | 500/250 µM

HepG2 cells

RelA

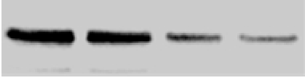

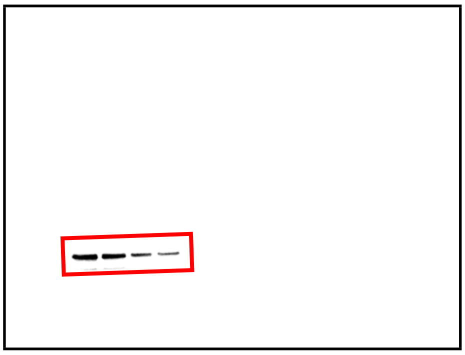

HNF1α

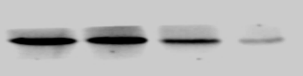

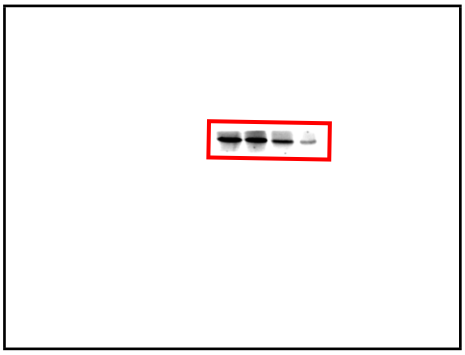

β-actin

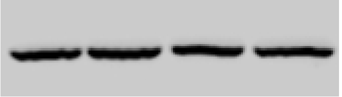

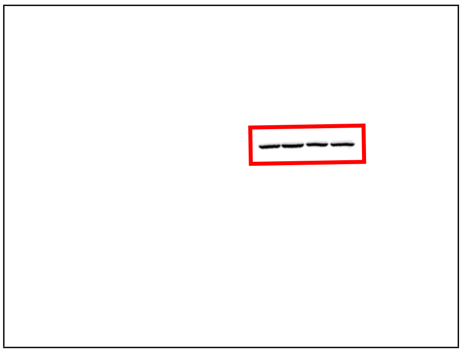

Fig. 1K

Control HFD Mice

Cleaved caspase-3

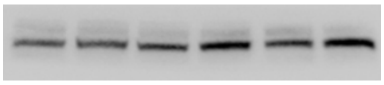

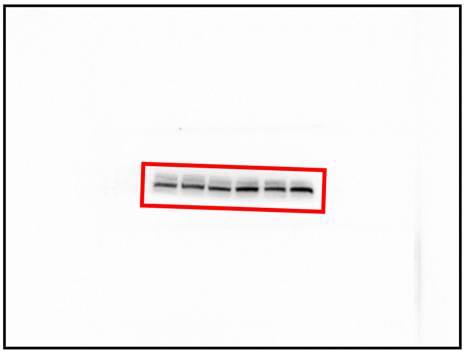

β-actin

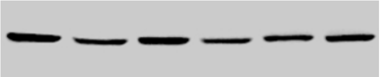

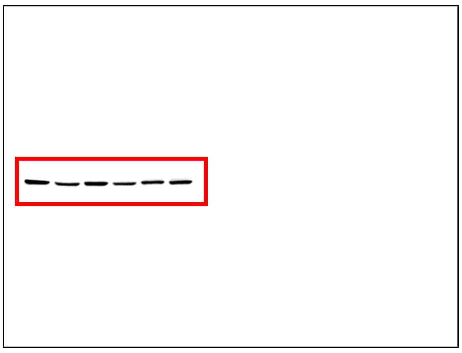

p-MLKL

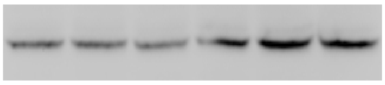

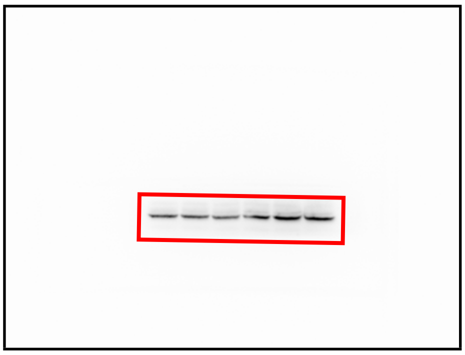

MLKL

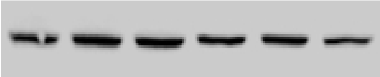

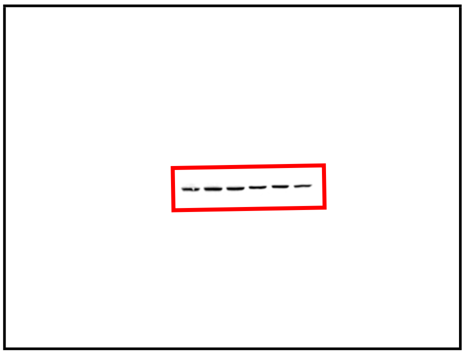

Fig 1L

Control HFD Mice

RelA

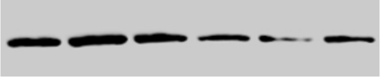

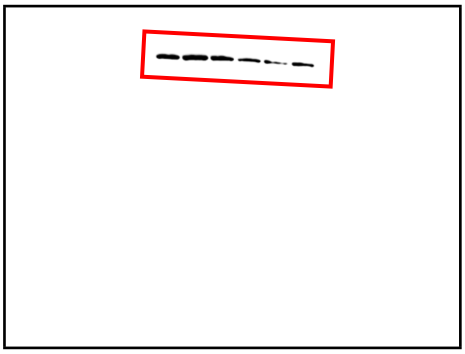

HNF1α

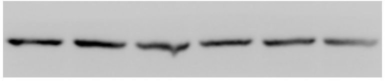

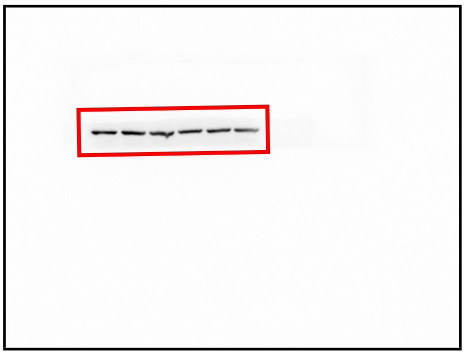

β-actin

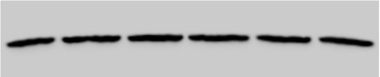

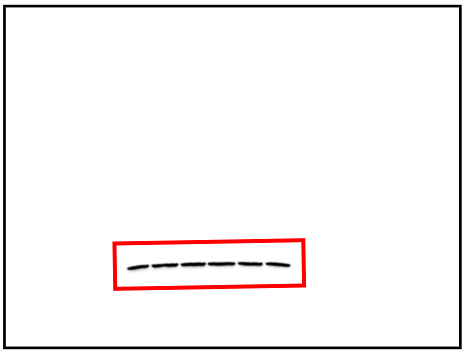

Fig. 2A

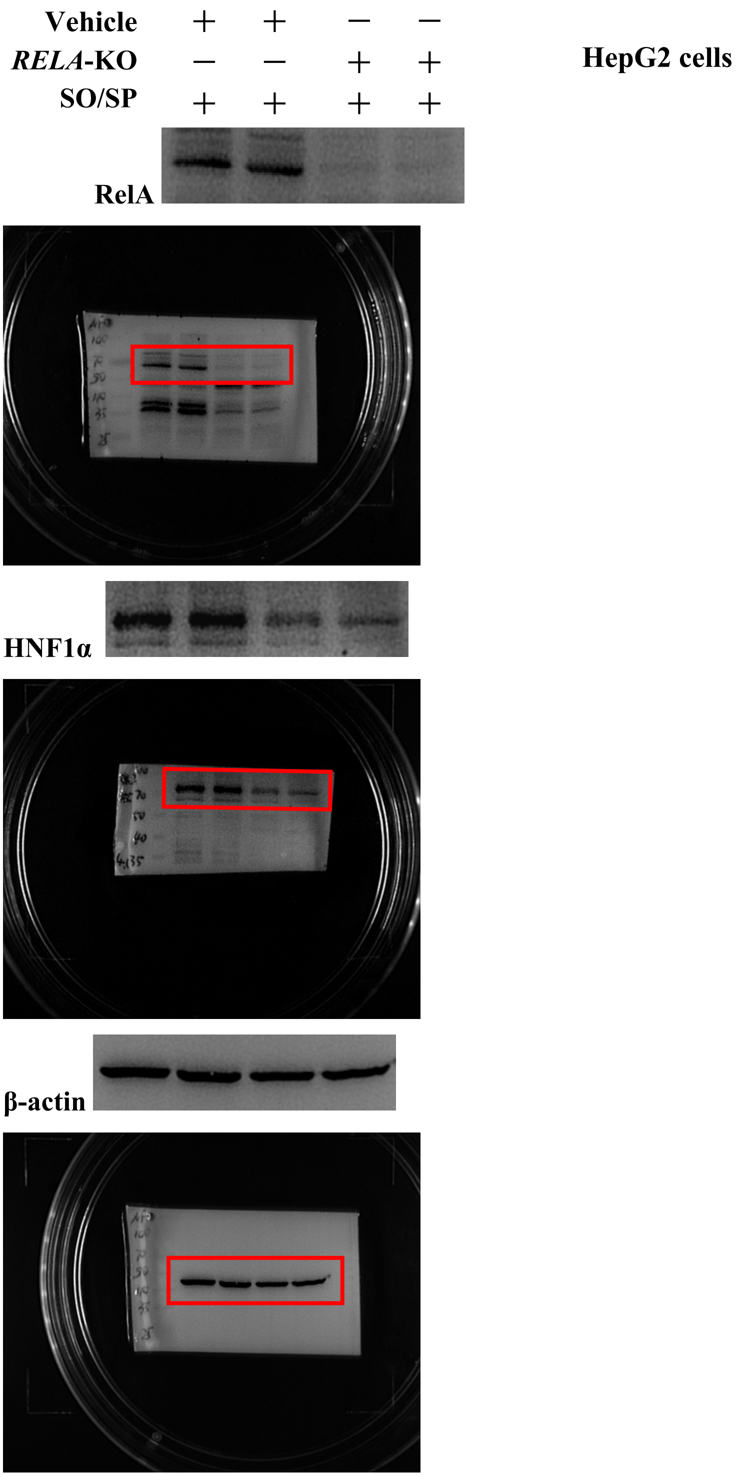

Fig. 2B

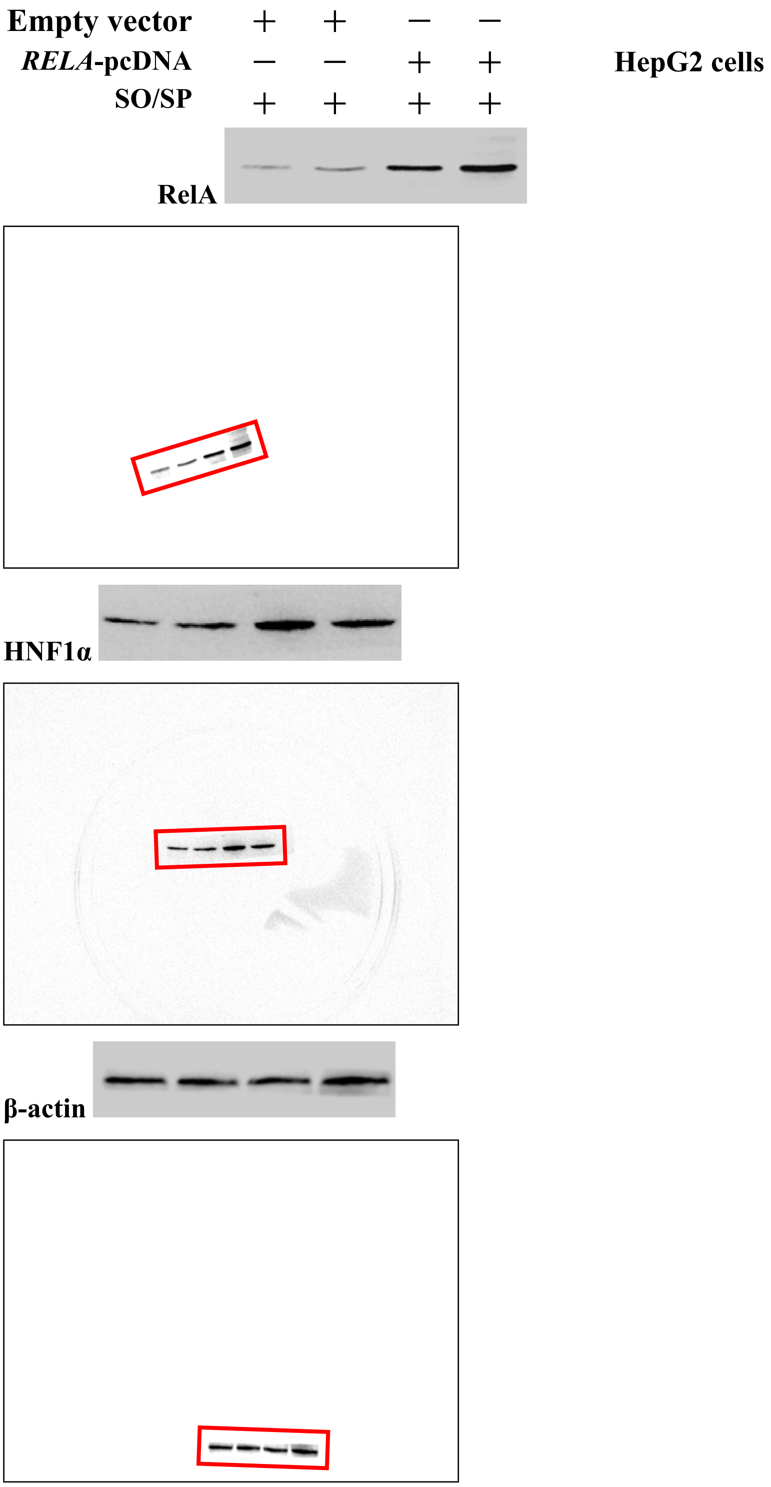

Fig. 2C

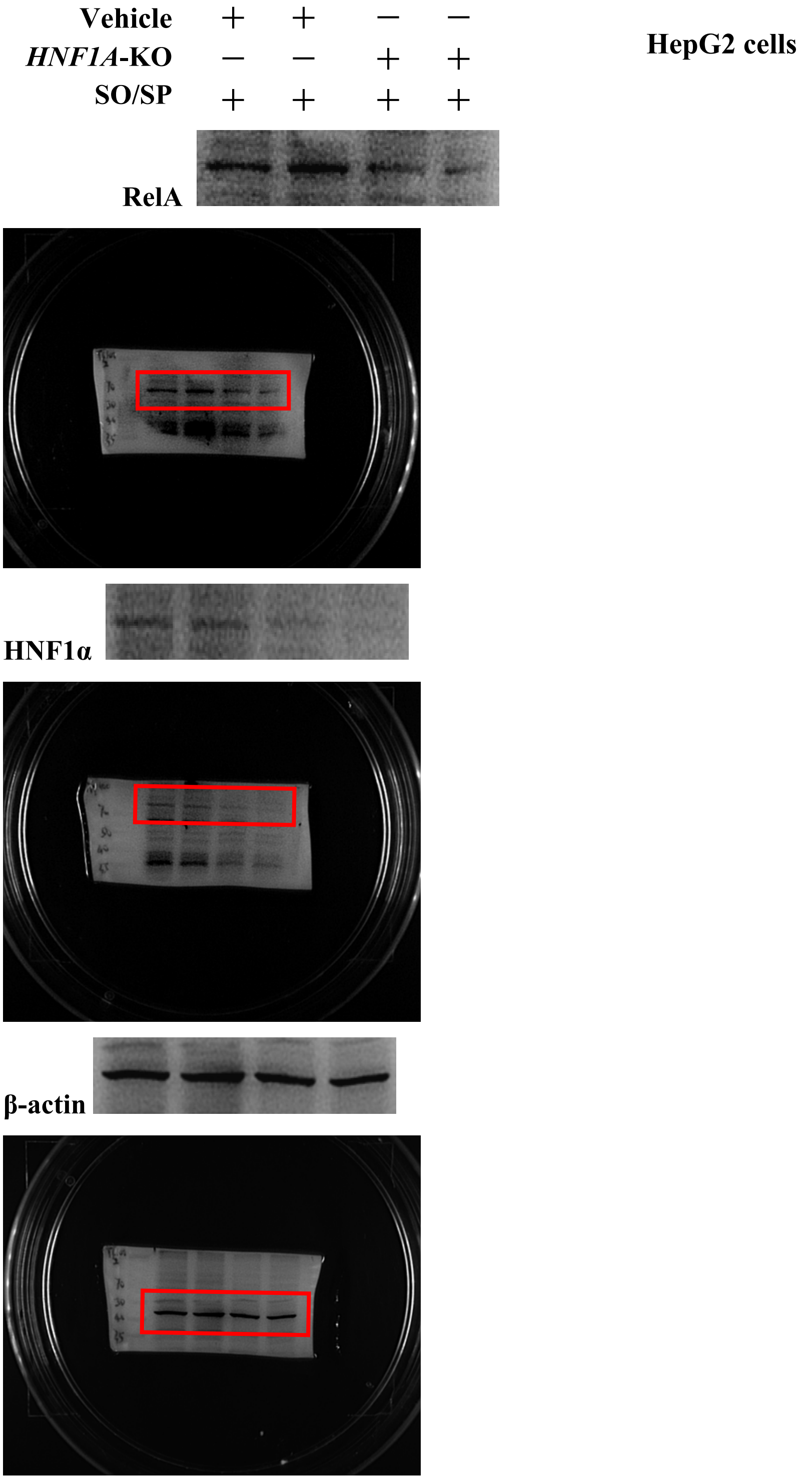

Fig. 2F

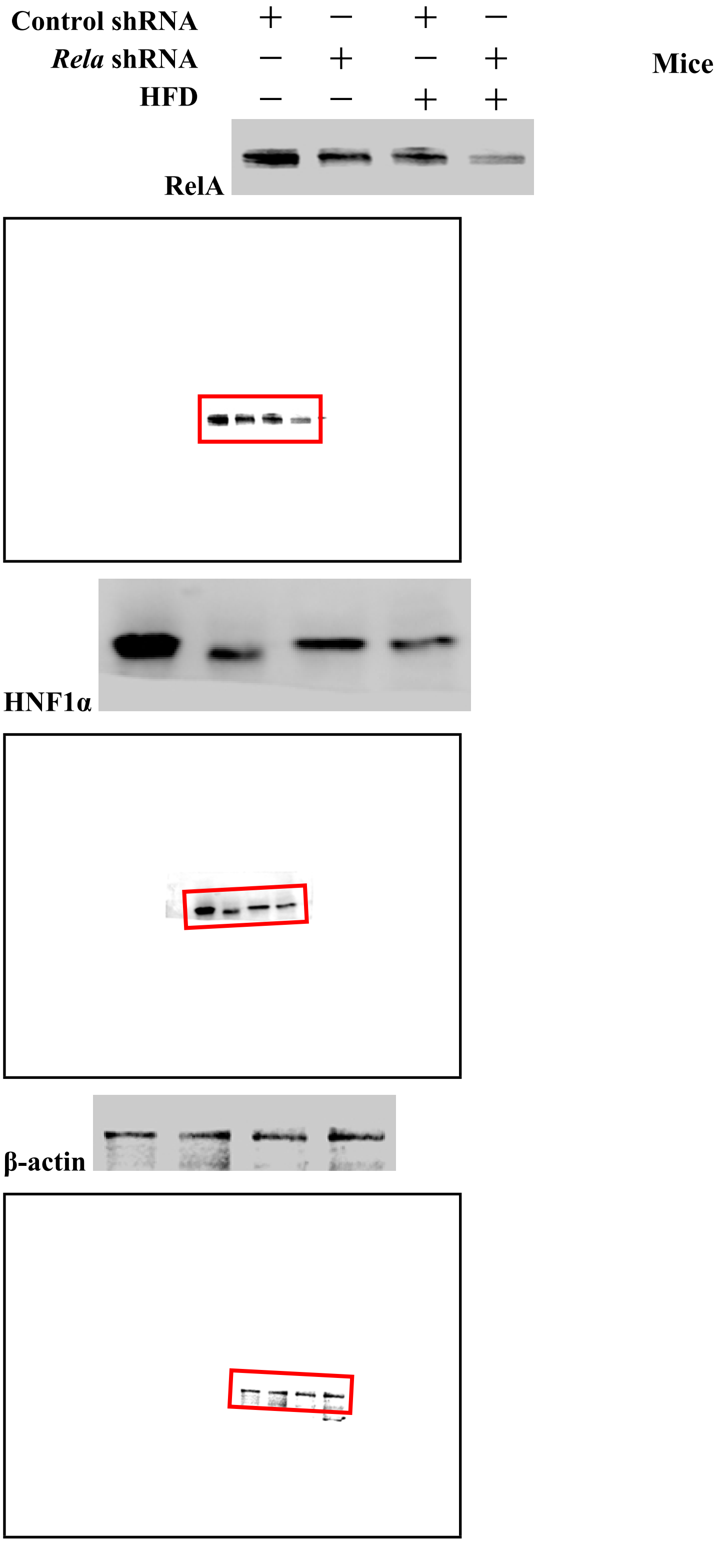

Fig. 2G

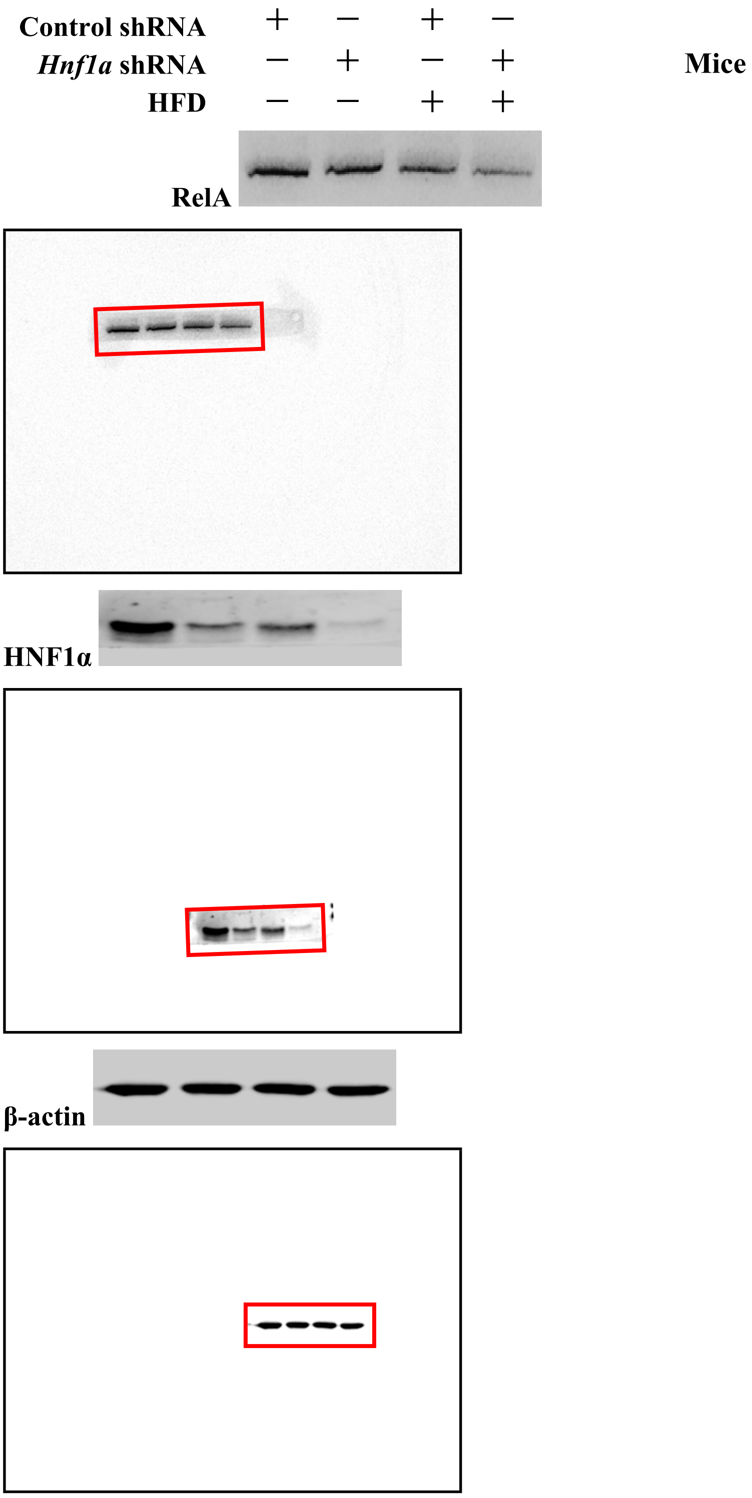

Fig. 3E

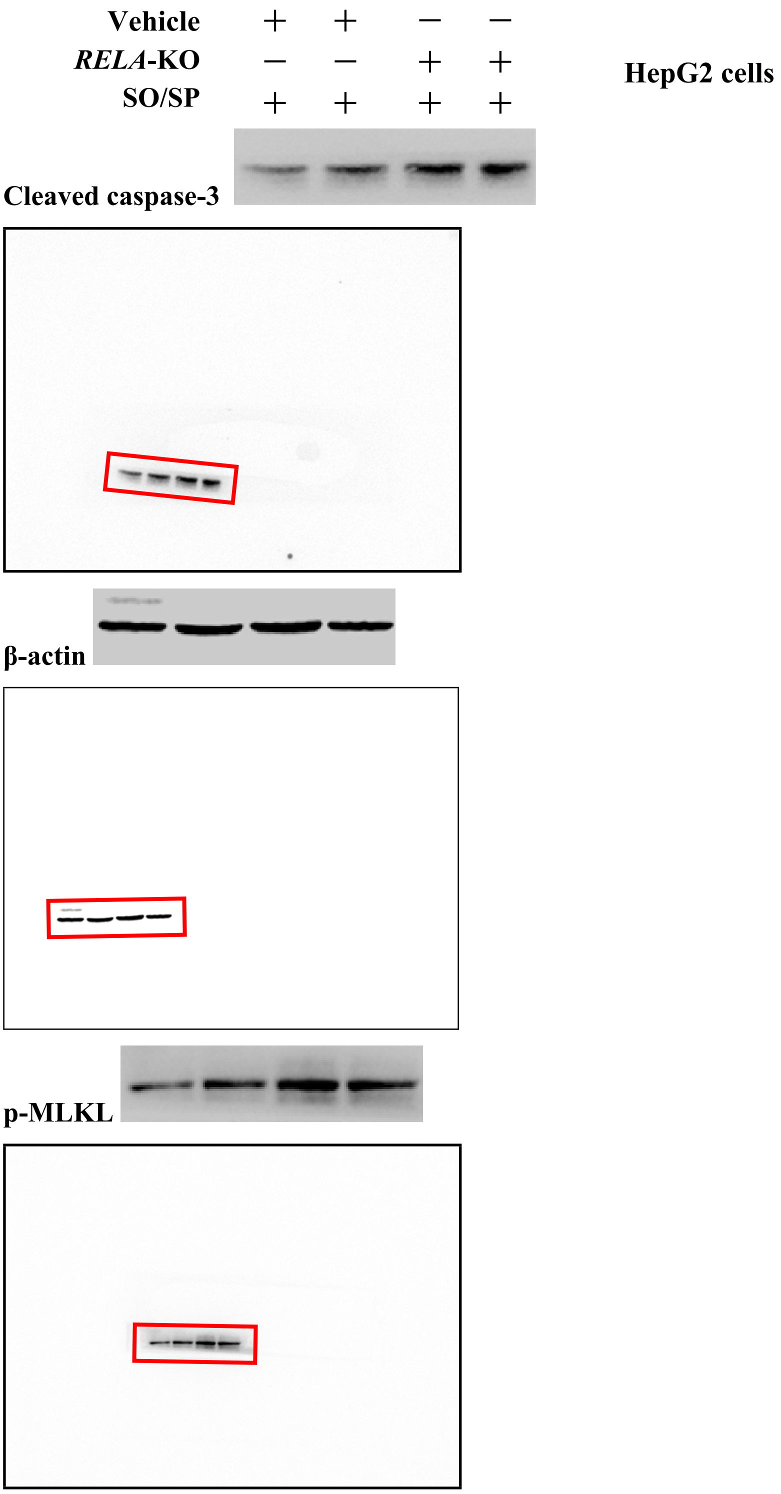
Vehicle + + − −
RELA-KO − − + +
SO/SP + + + +
HepG2 cells
Cleaved caspase-3
β-actin
p-MLKL

MLKL

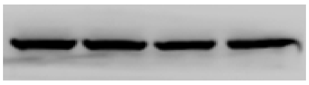

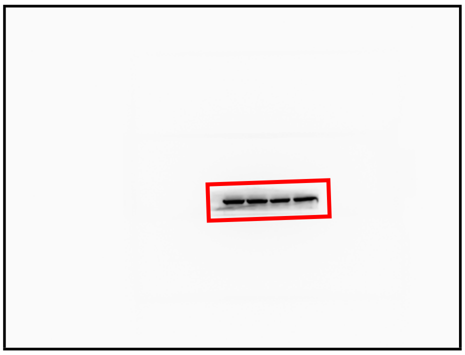

Fig. 3J

Empty vector	+	+	–	–	HepG2 cells
RELA-pcDNA	–	–	+	+	
SO/SP	+	+	+	+	

Cleaved caspase-3

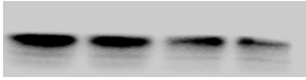

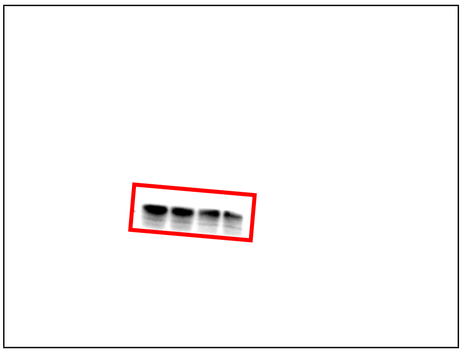

β-actin

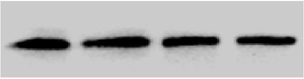

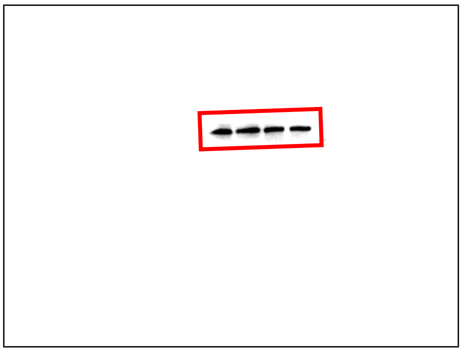

p-MLKL

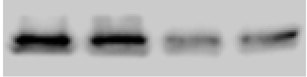

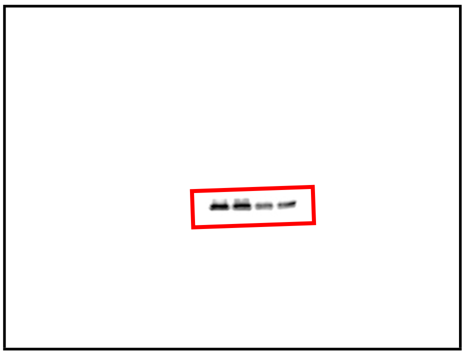

MLKL

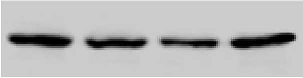

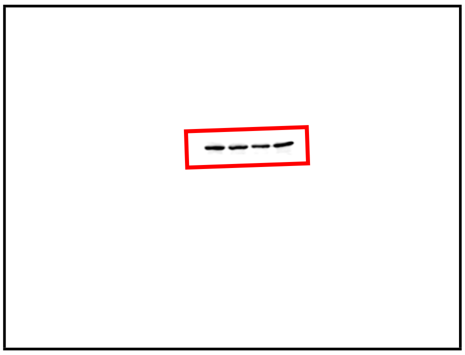

Fig. 4F

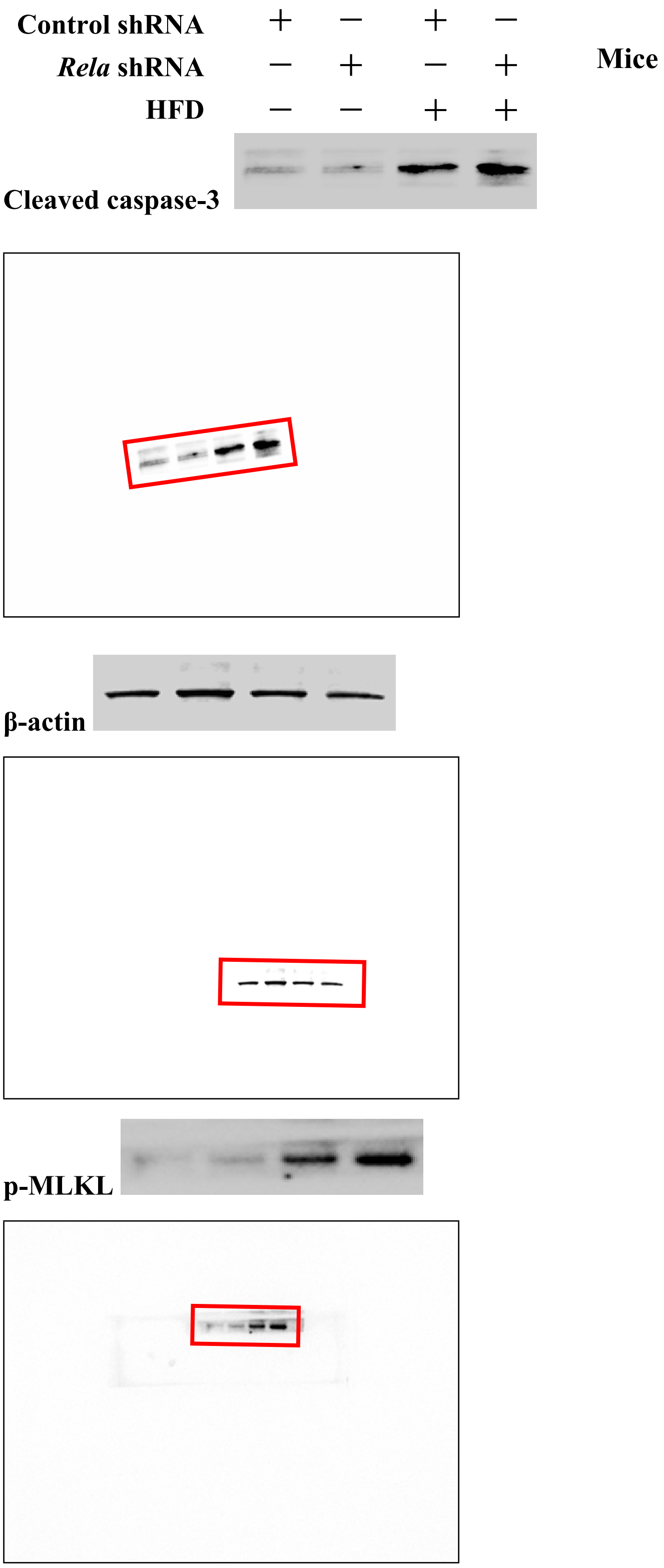

Control shRNA + − + −
Rela shRNA − + − +
HFD − − + +
Mice
Cleaved caspase-3
β-actin
p-MLKL

MLKL 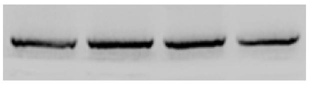

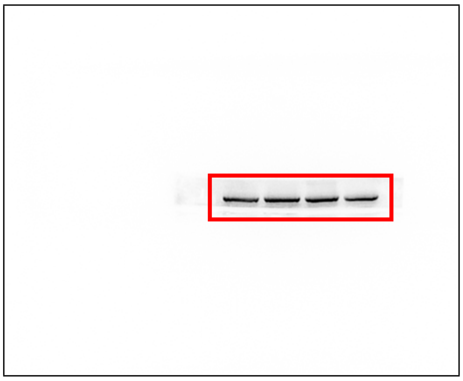

Fig. 5E

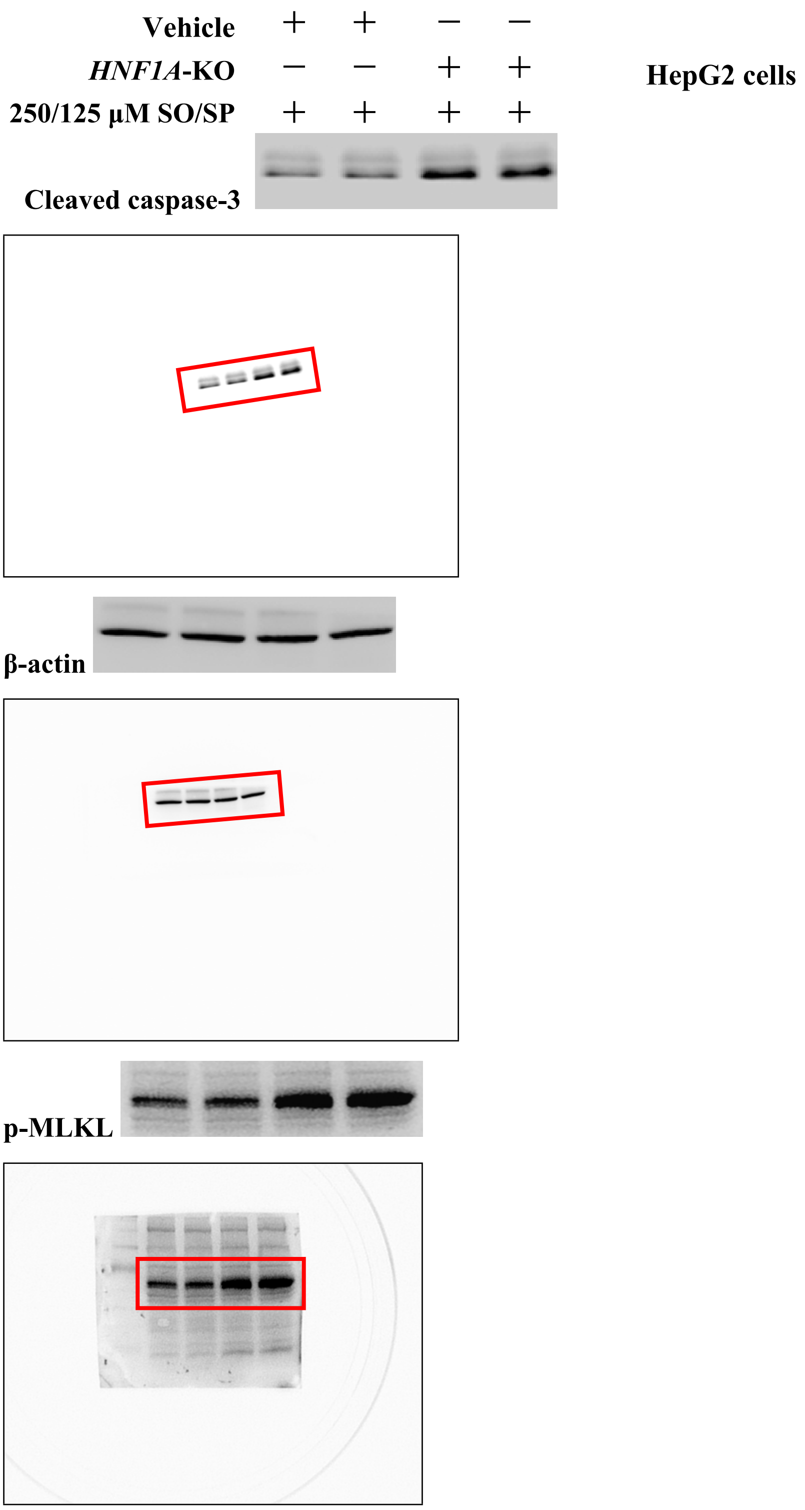
Vehicle + + − −
HNF1A-KO − − + +
250/125 µM SO/SP + + + +
HepG2 cells
Cleaved caspase-3
β-actin
p-MLKL

MLKL

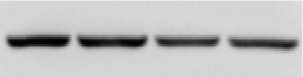

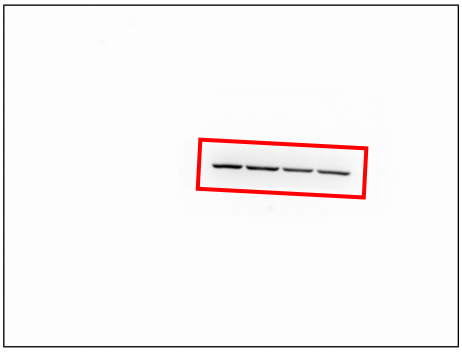

Fig. 5K

Control shRNA	+	−	+	−	
Hnf1a shRNA	−	+	−	+	Mice
HFD	−	−	+	+	

Cleaved caspase-3

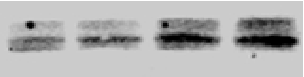

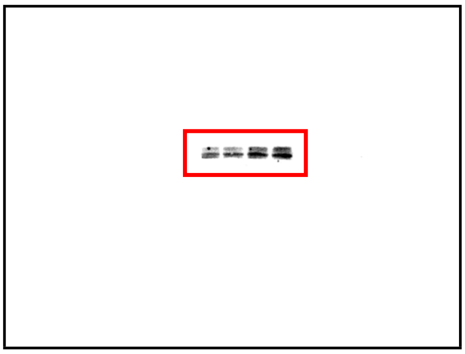

β-actin

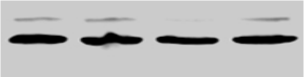

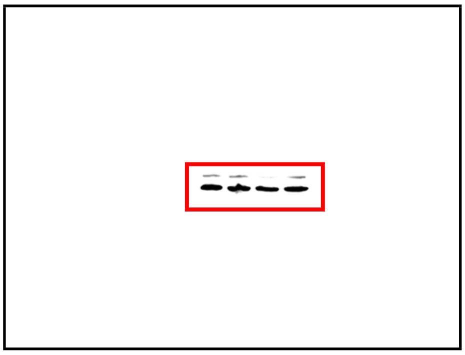

p-MLKL

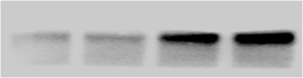

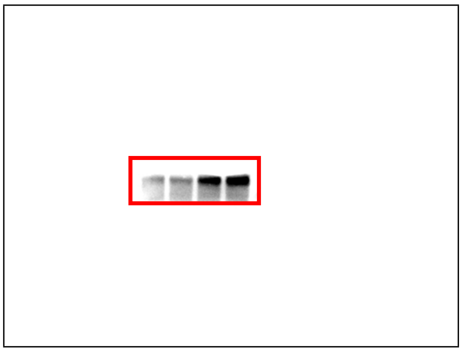

MLKL

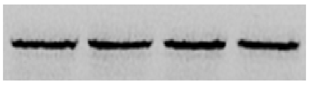

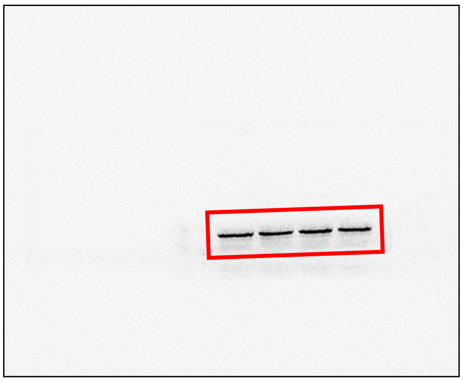

Fig. 6G

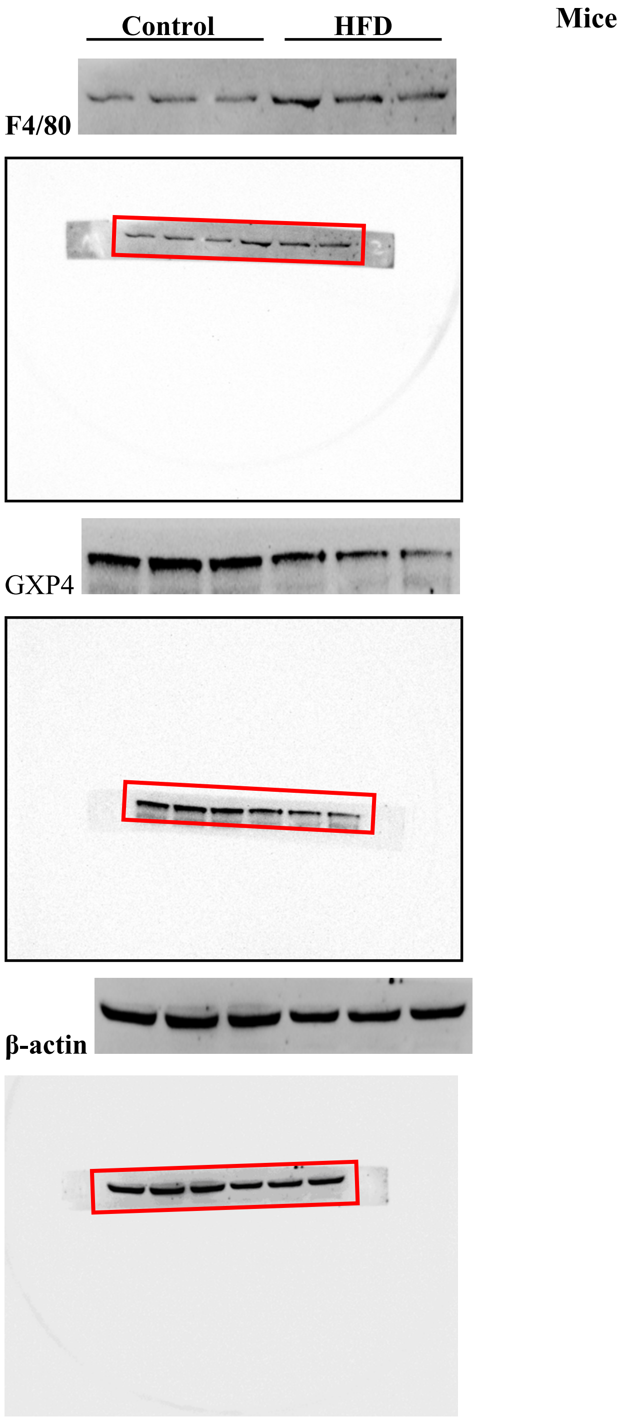

Fig. 6H

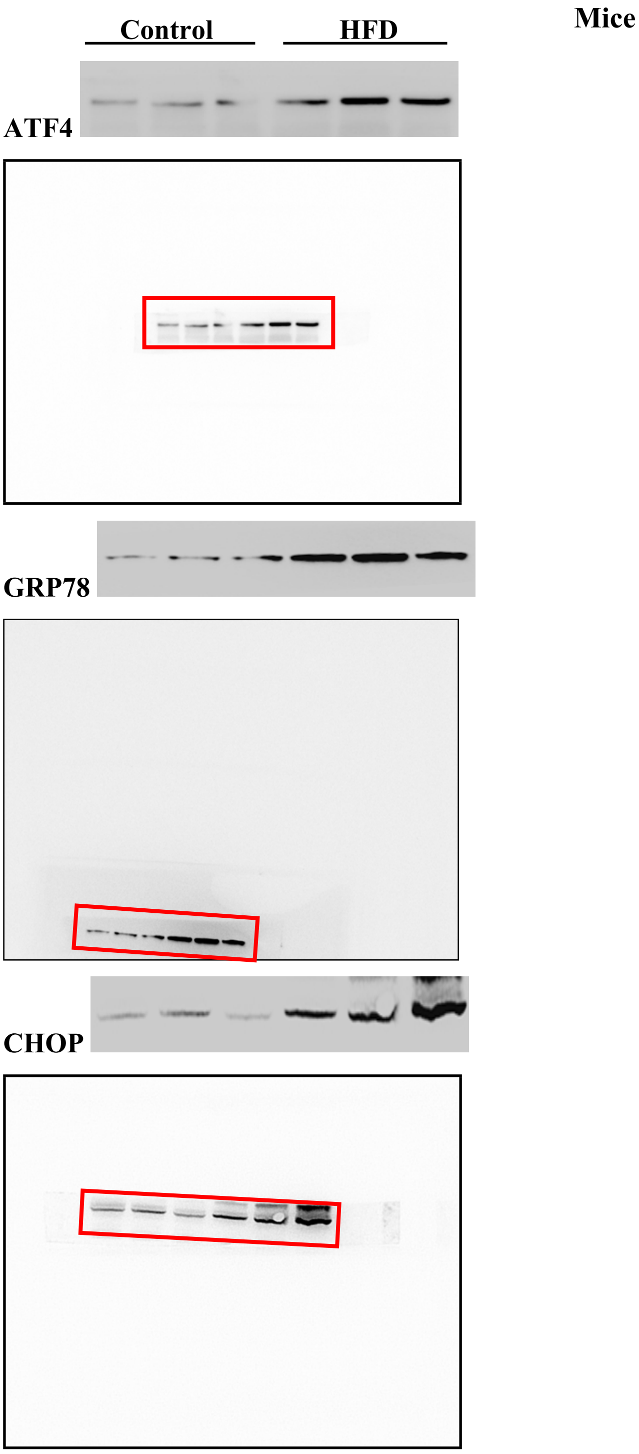

β-actin

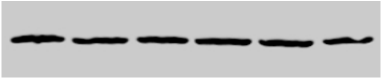

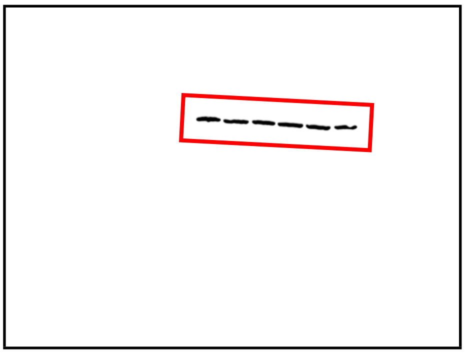

Fig. 7D

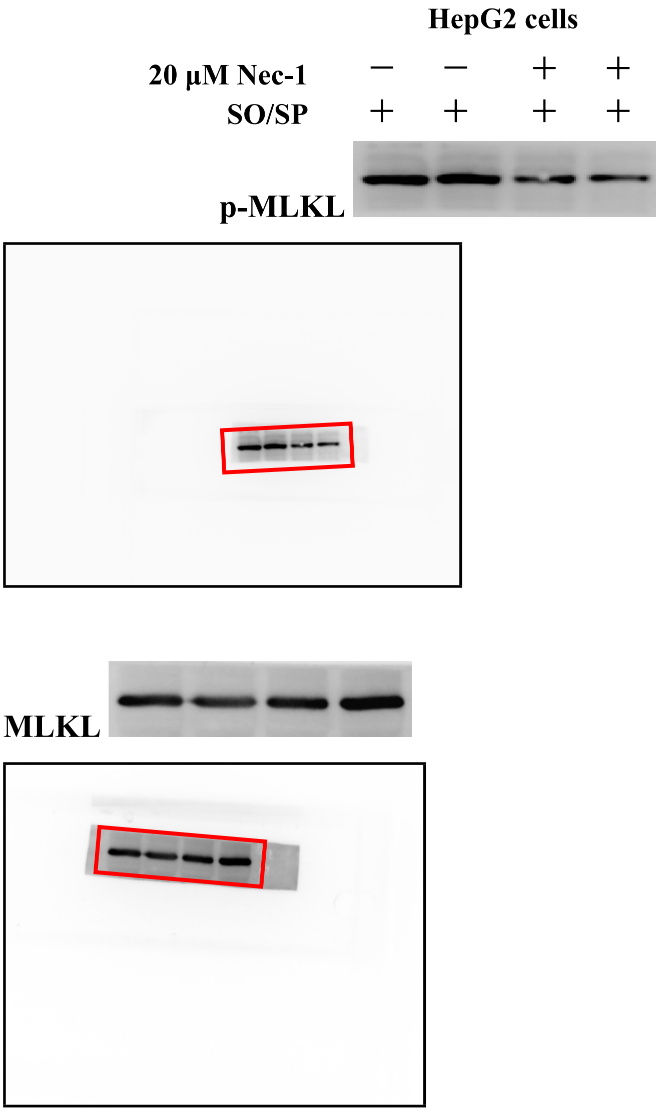

Fig. 7I

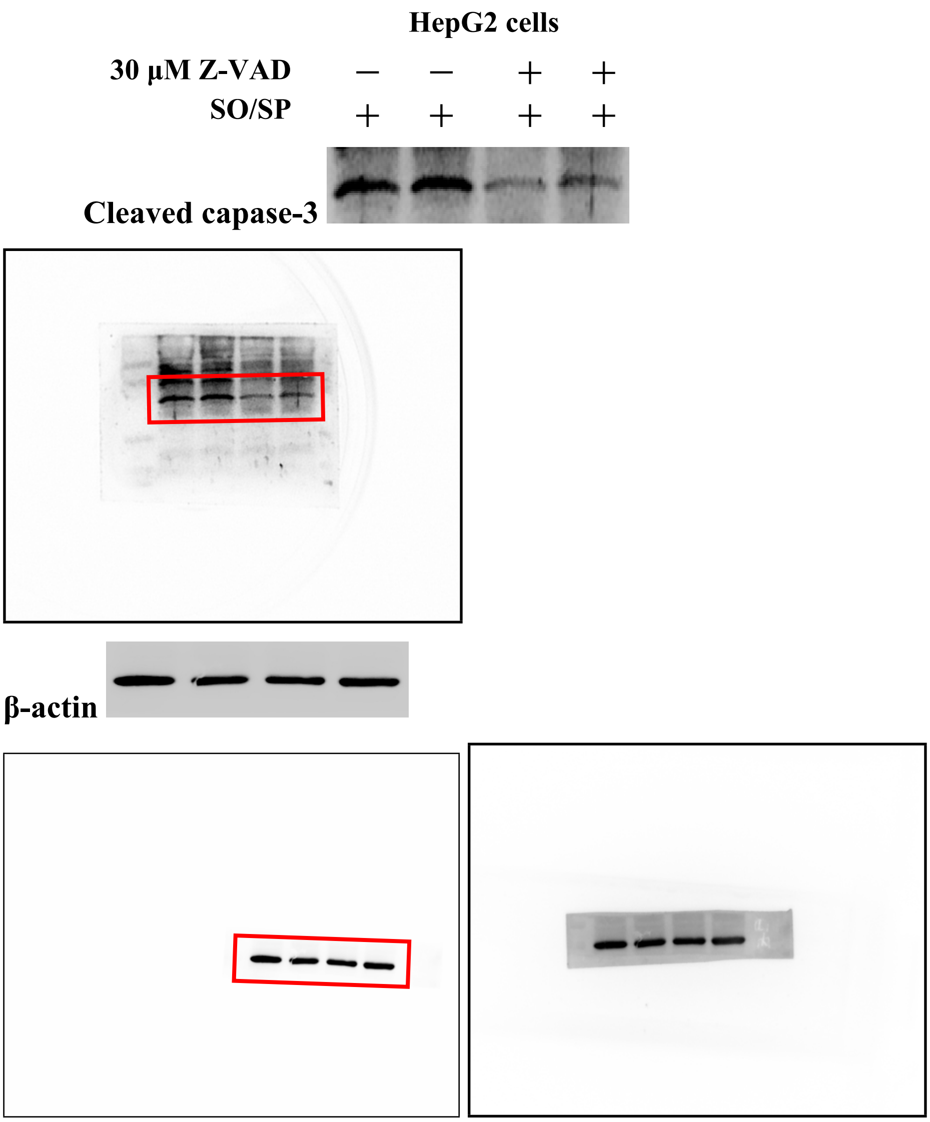

Fig. 8G

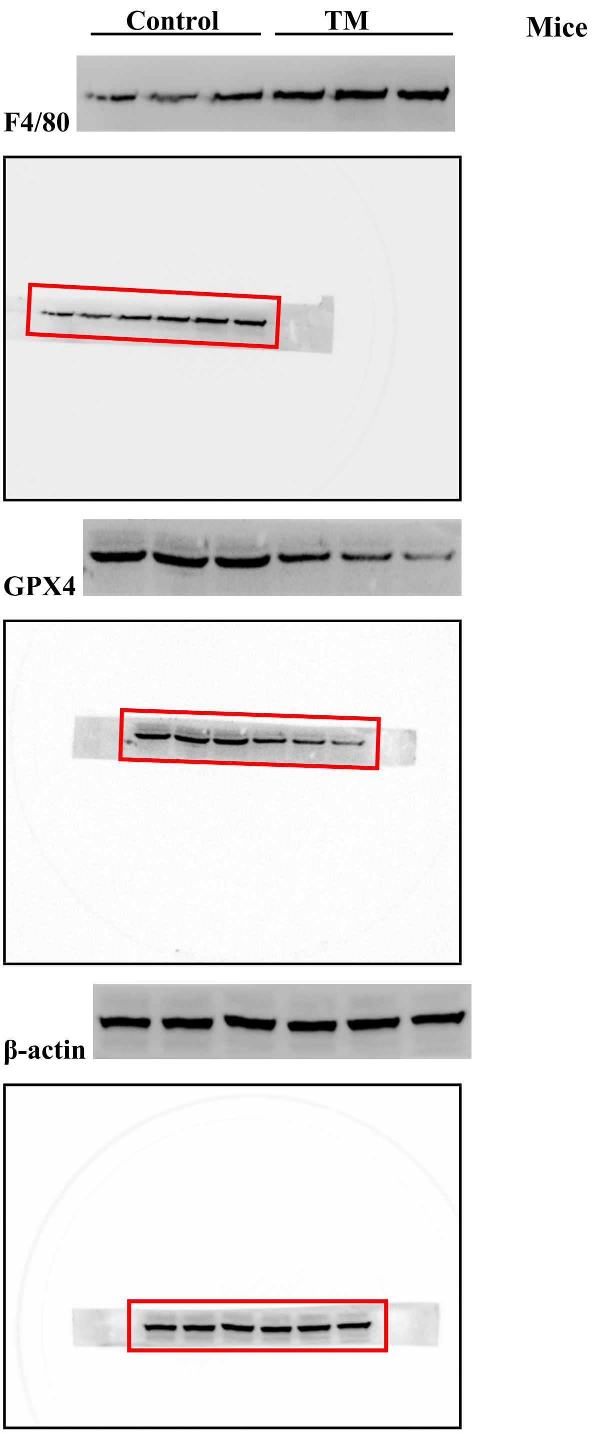

Fig. 8H

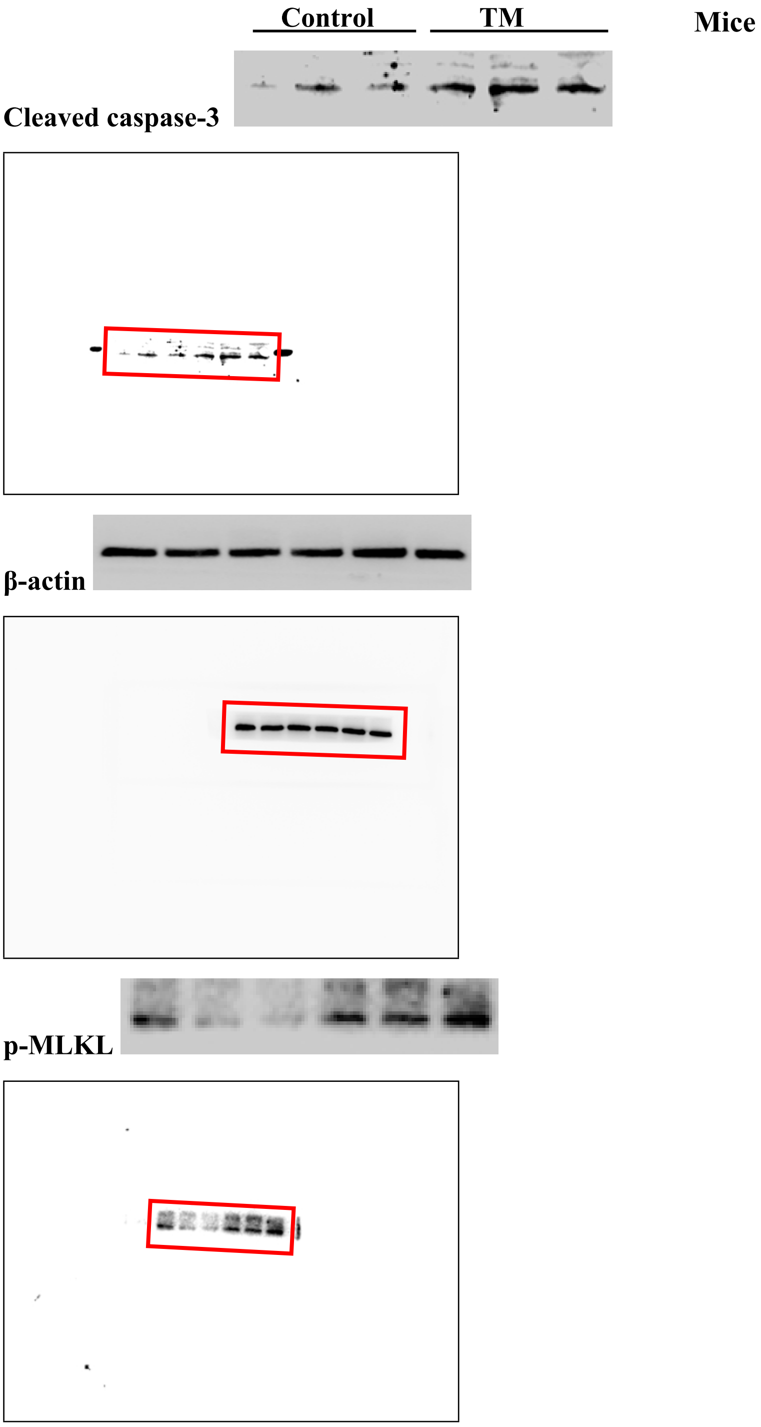

MLKL

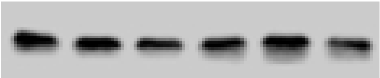

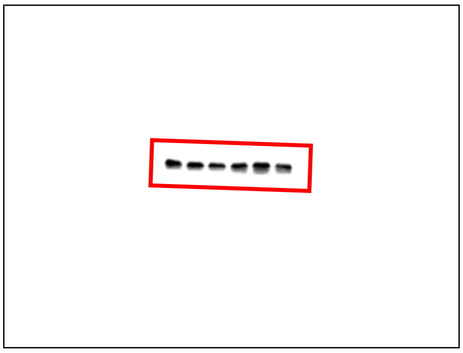

Fig. 9A

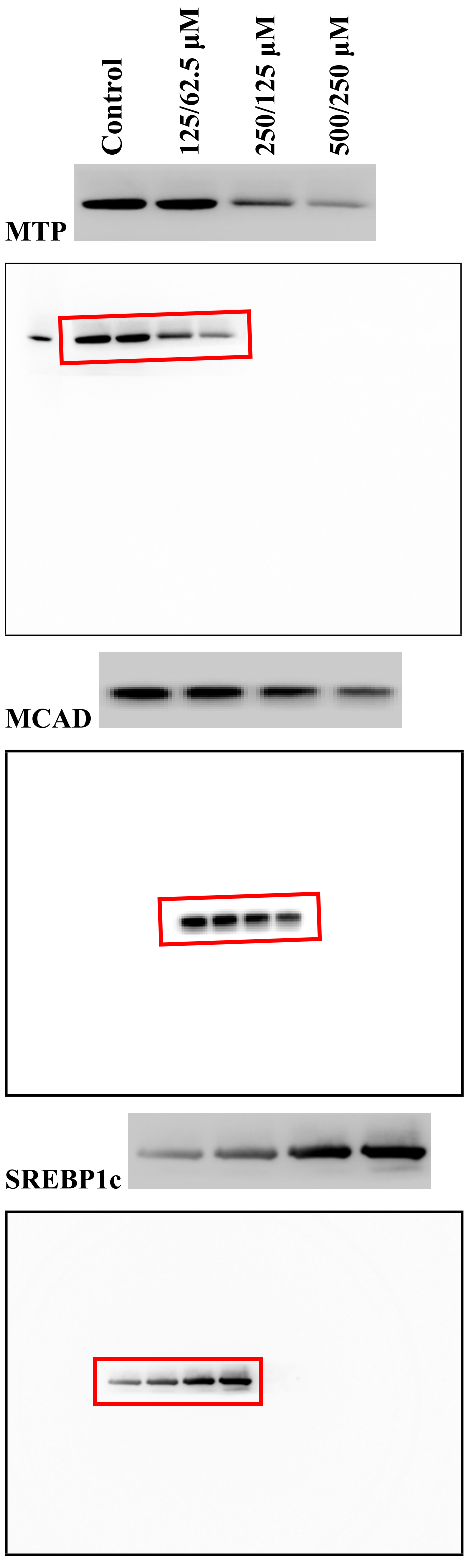

HepG2 cells

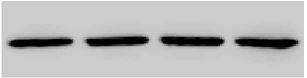

β-actin

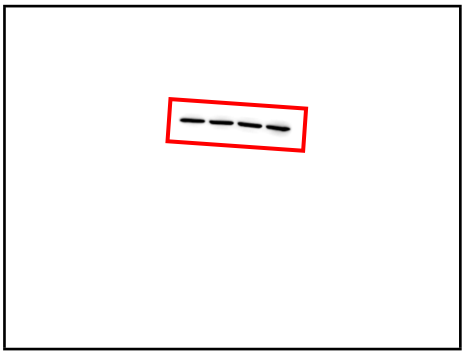

Fig. 9B

Control 48h THA

HepG2 cells

MTP

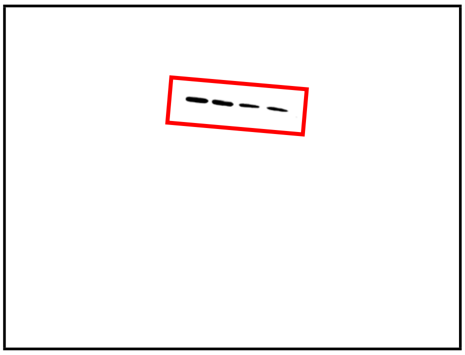

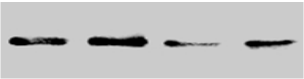

MCAD

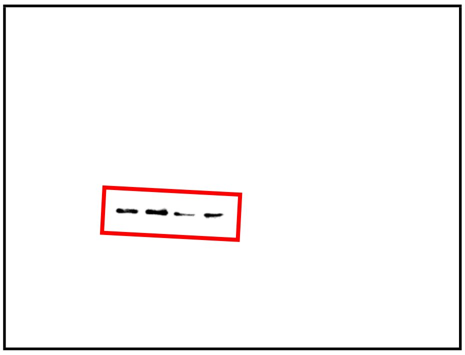

SREBP1c

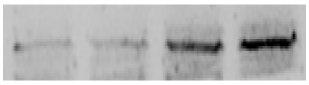

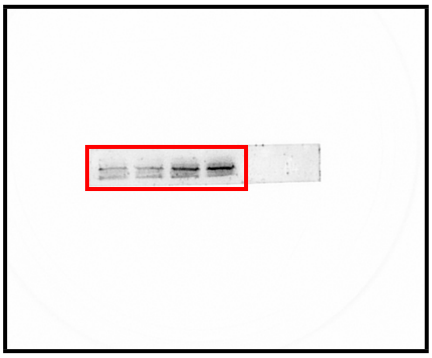

β-actin

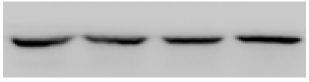

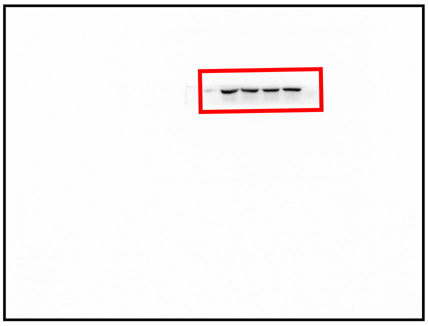

Fig. 9C

Vehicle	+	+	−	−	
RELA-KO	−	−	+	+	HepG2 cells
SO/SP	+	+	+	+	

MTP

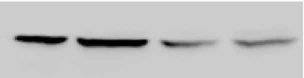

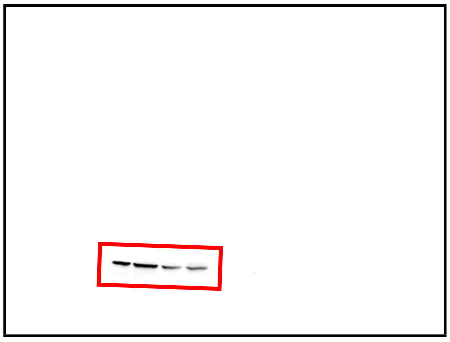

MCAD

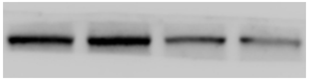

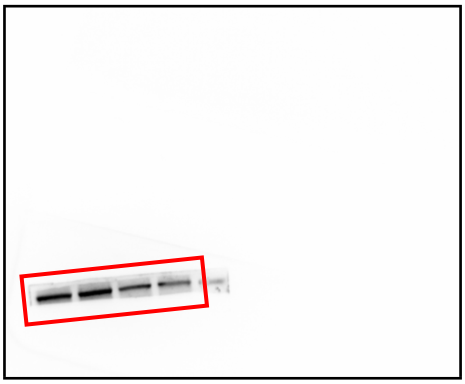

SREBP1c

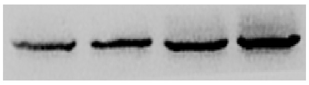

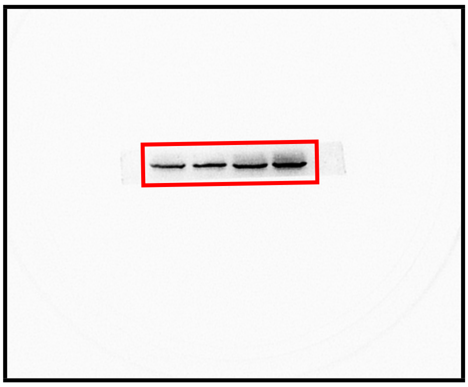

β-actin

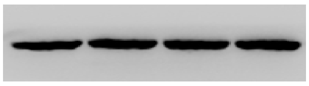

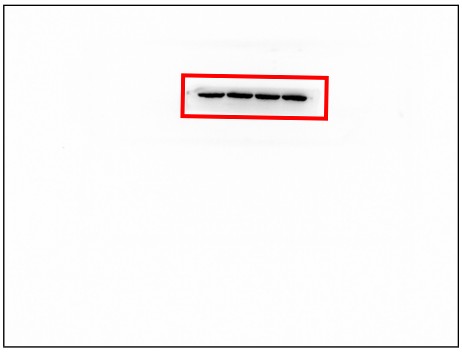

Fig. 9D

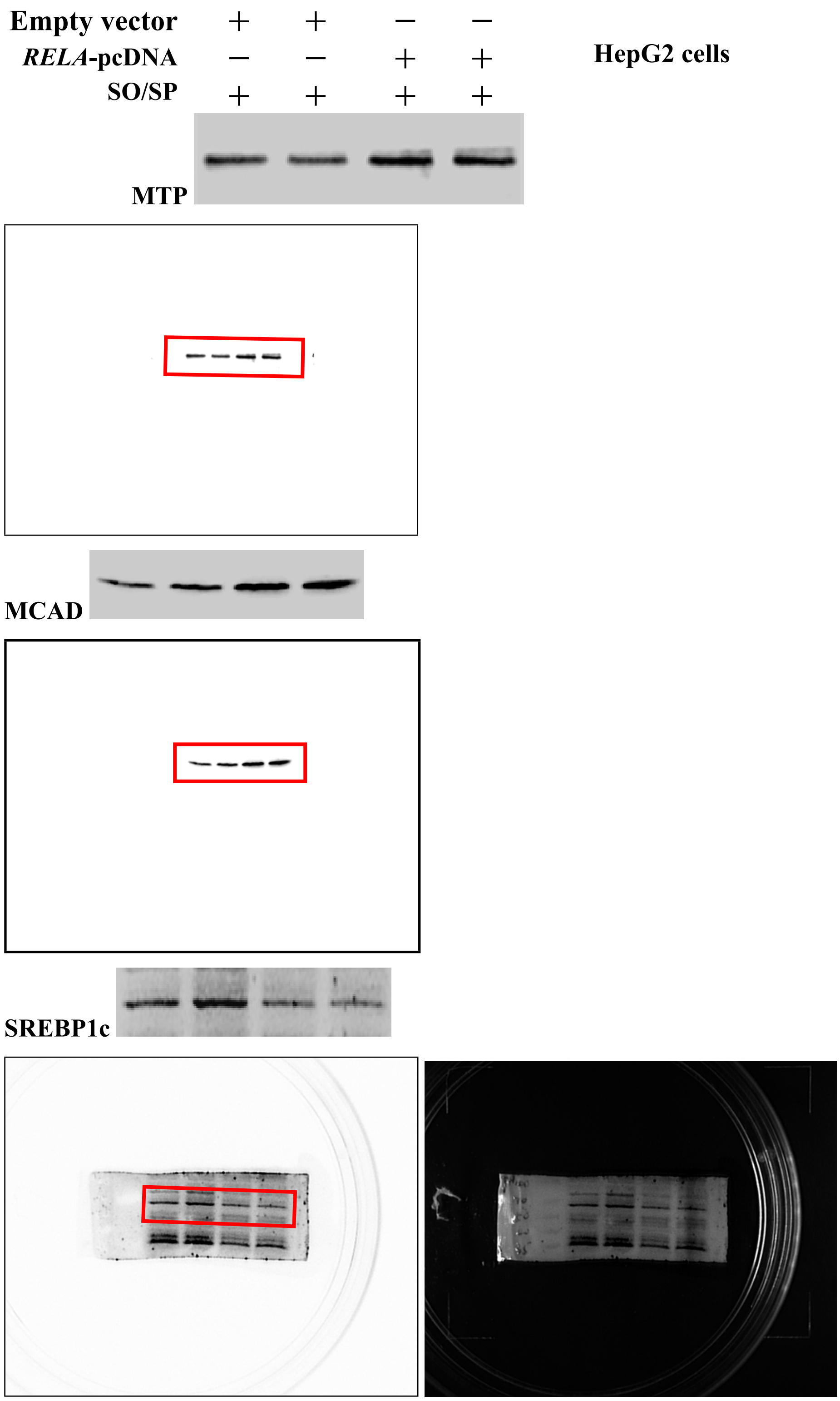

β-actin 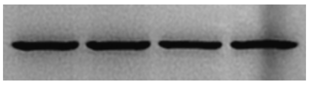

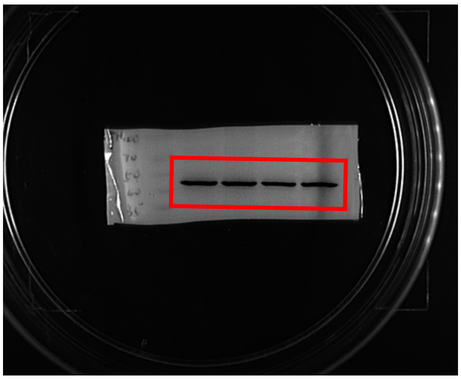

Fig. 9I

Control HFD

Mice

MTP 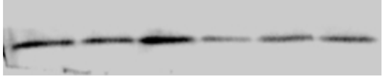

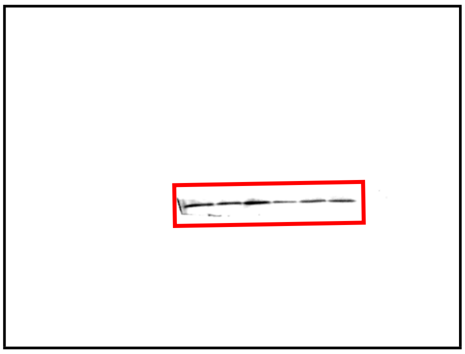

MCAD 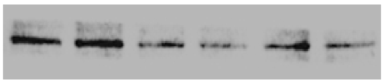

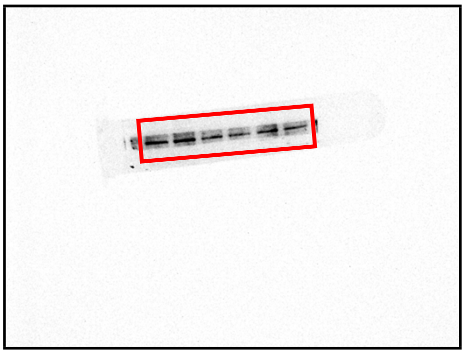

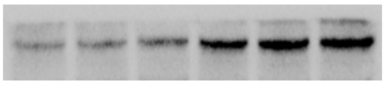

SREBP1c

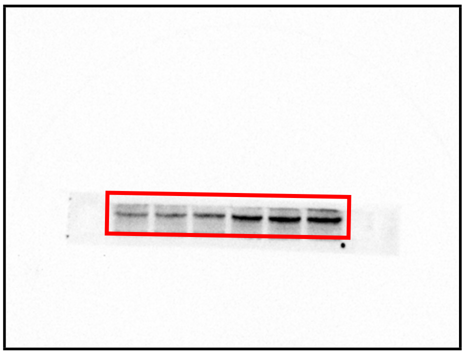

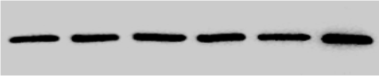

β-actin

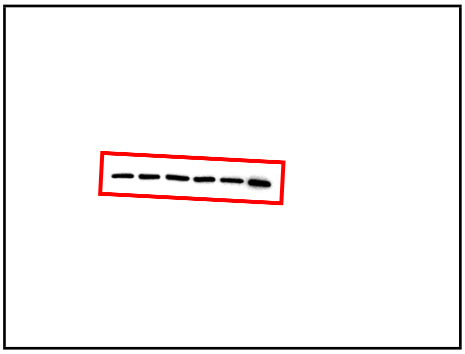

Fig. 9J

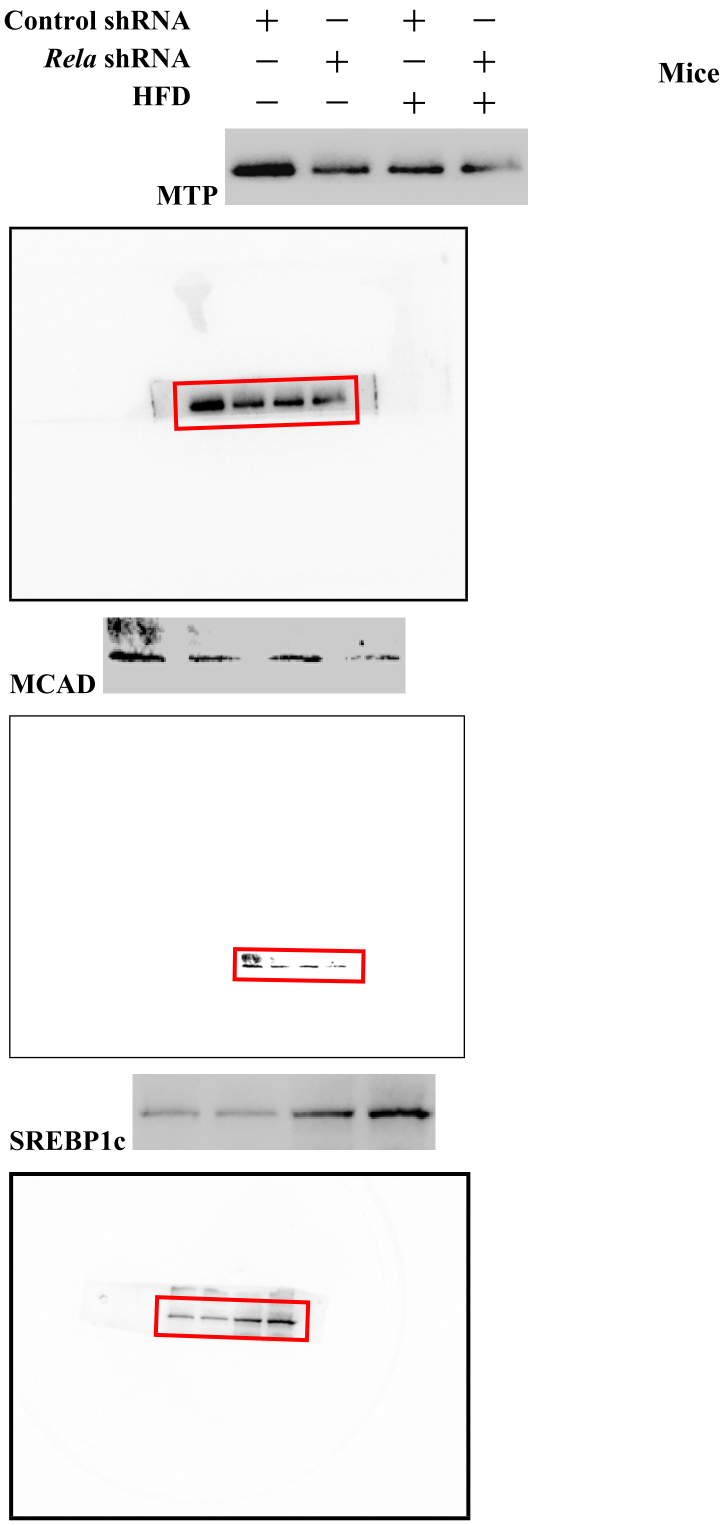

β-actin

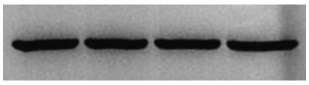

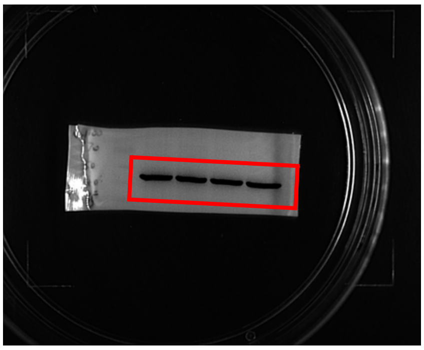

Fig. 10H

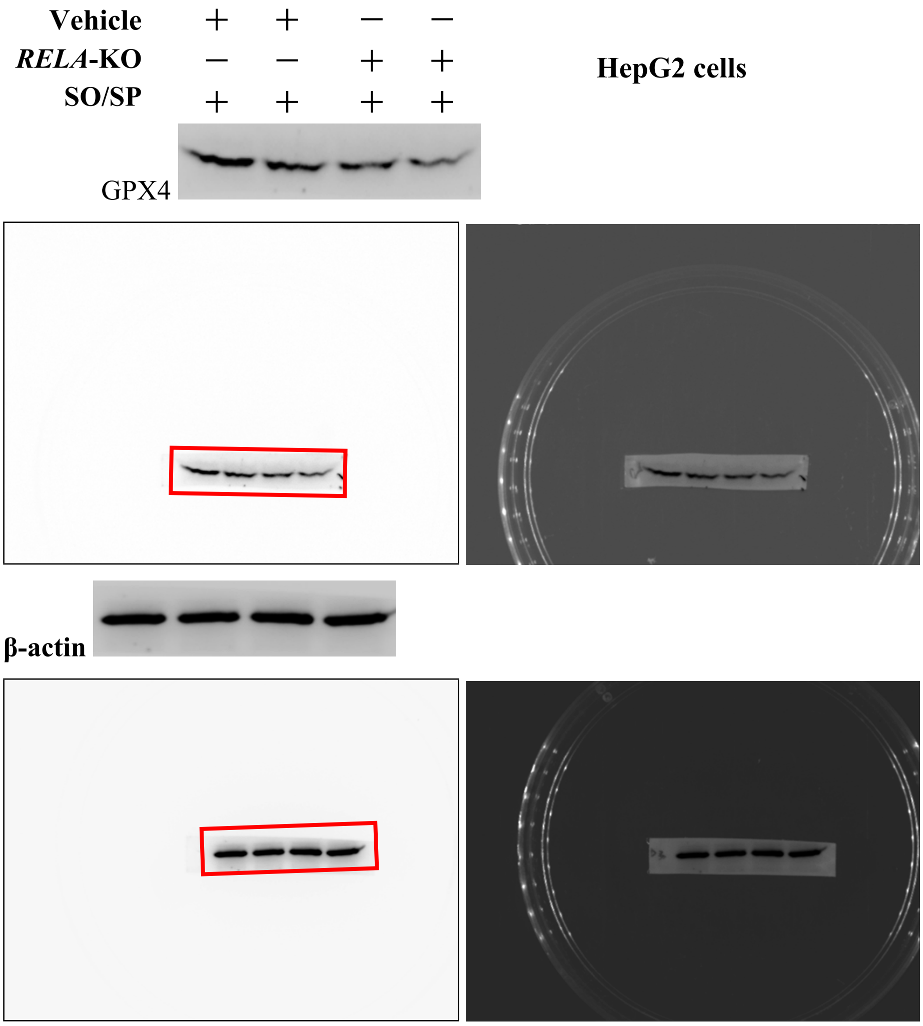

Fig. 10I

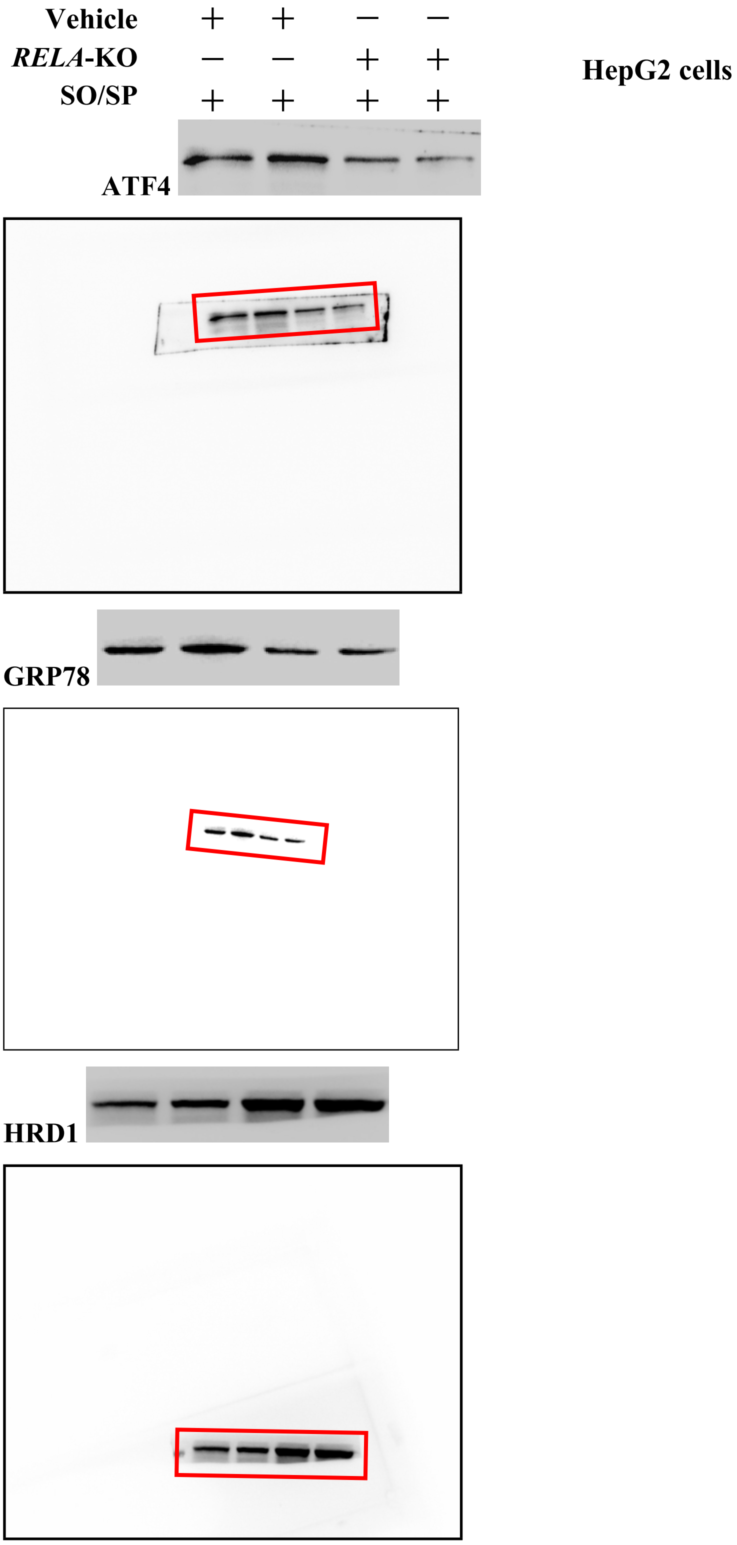

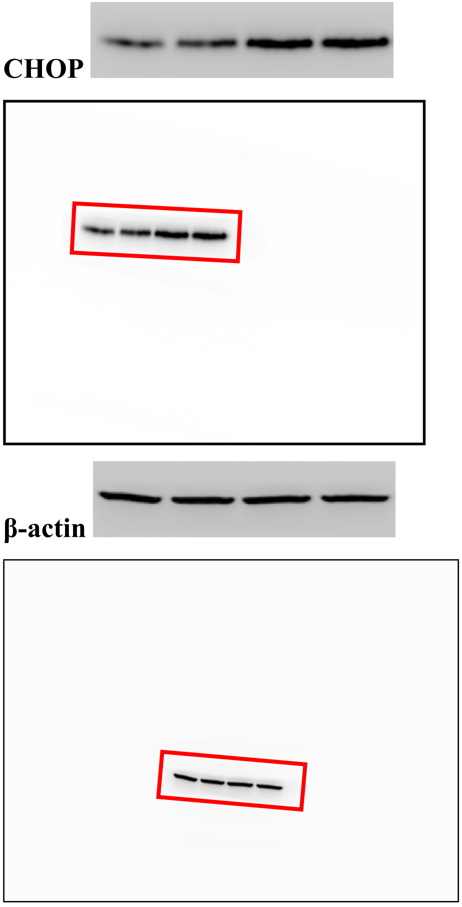

Fig. 10J

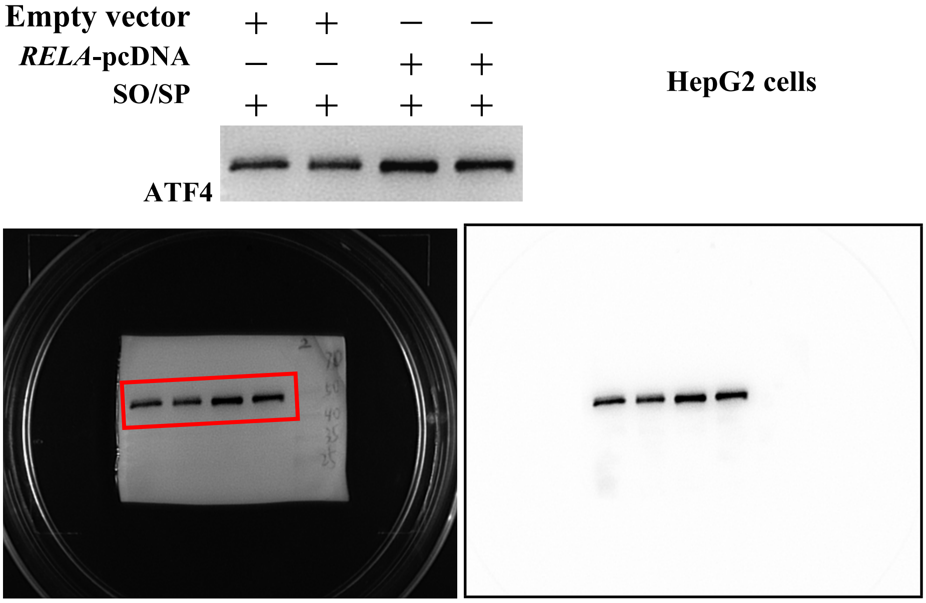

GRP78

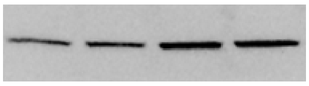

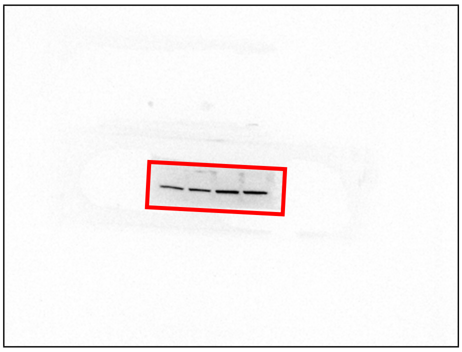

HRD1

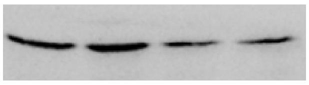

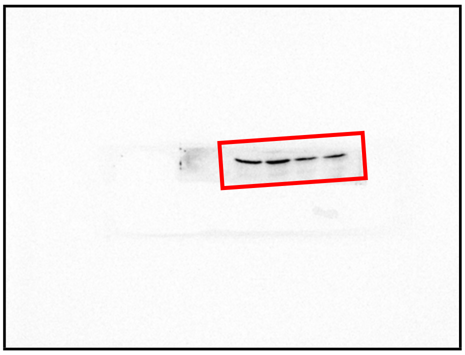

CHOP

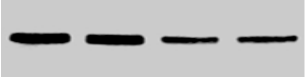

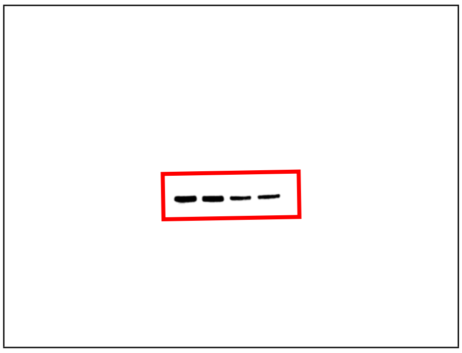

β-actin

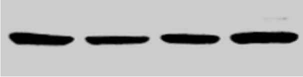

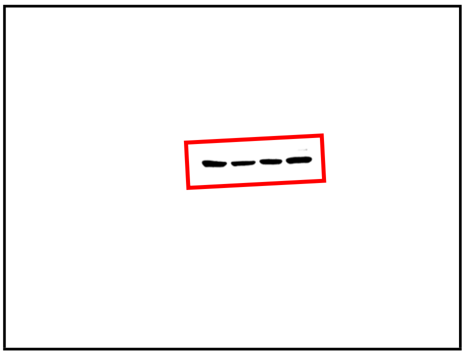

Fig. 10L

Control shRNA	+	−	+	−	
Rela shRNA	−	+	−	+	Mice
HFD	−	−	+	+	

ATF4

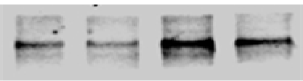

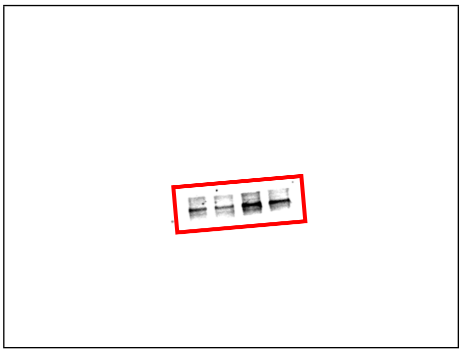

GRP78

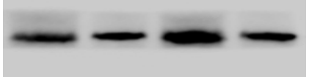

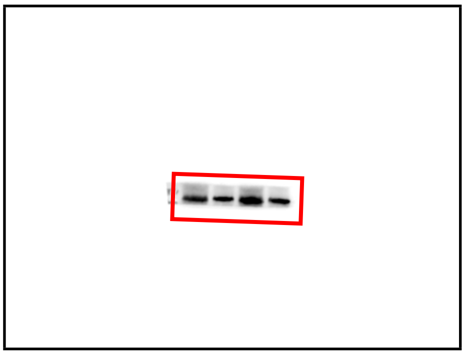

HRD1 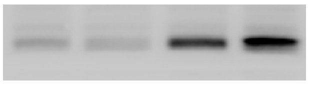

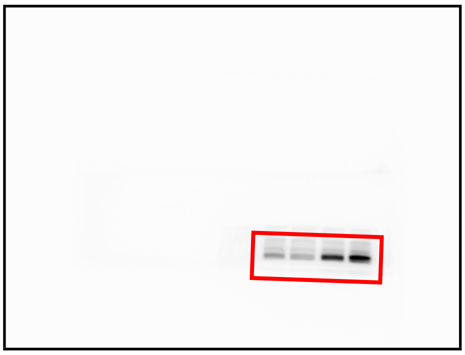

CHOP 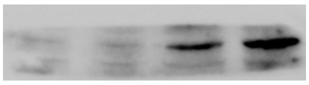

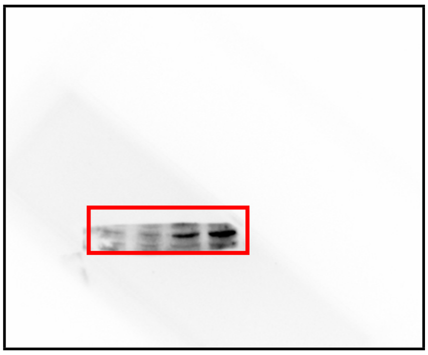

β-actin 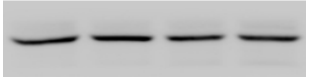

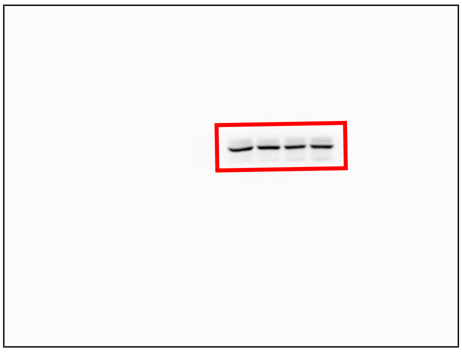

Supplementary Fig. 1D

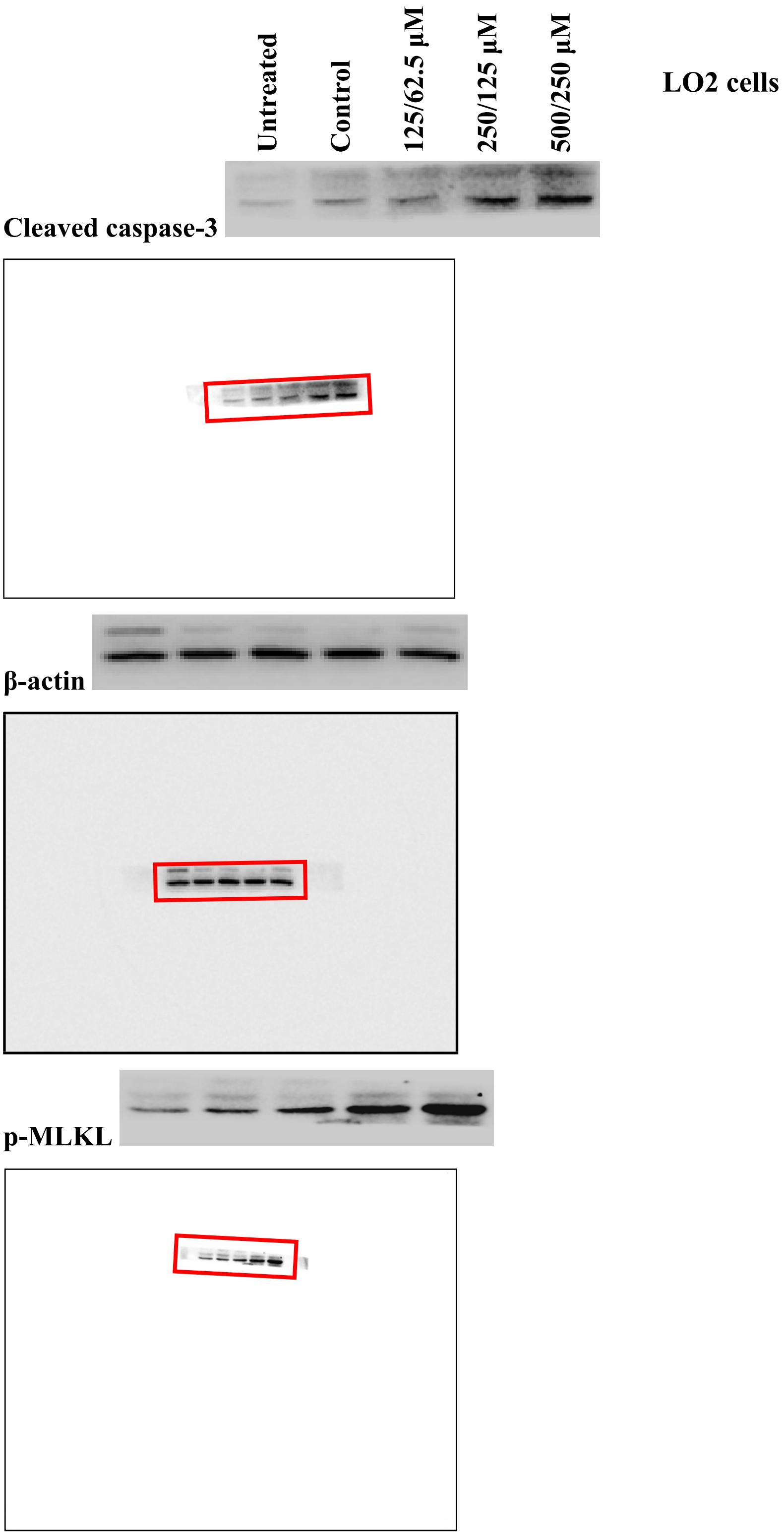

MLKL

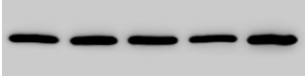

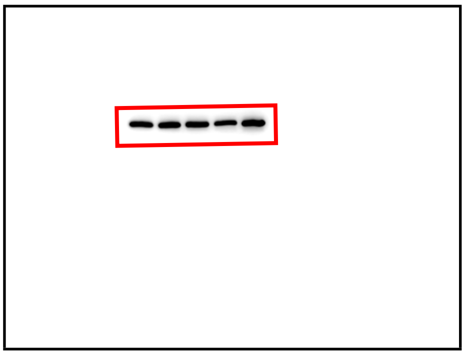

Supplementary Fig. 1E

Untreated

Control

125/62.5 µM

250/125 µM

500/250 µM

LO2 cells

RelA

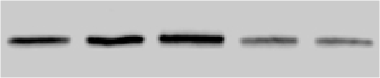

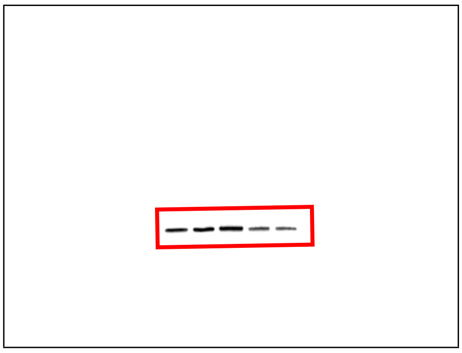

HNF1α

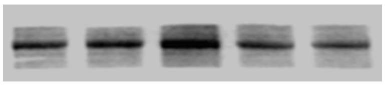

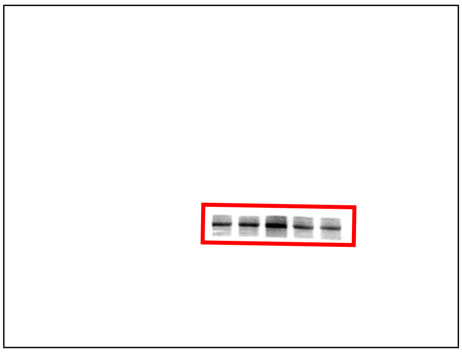

β-actin
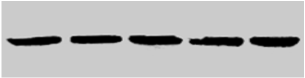

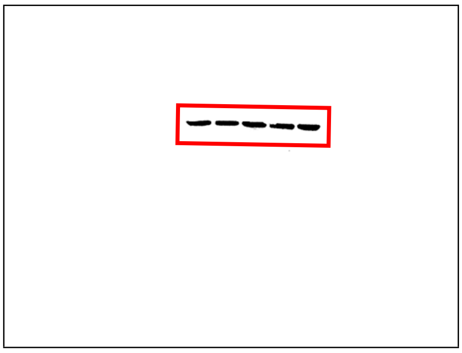

Supplementary Fig. 2A

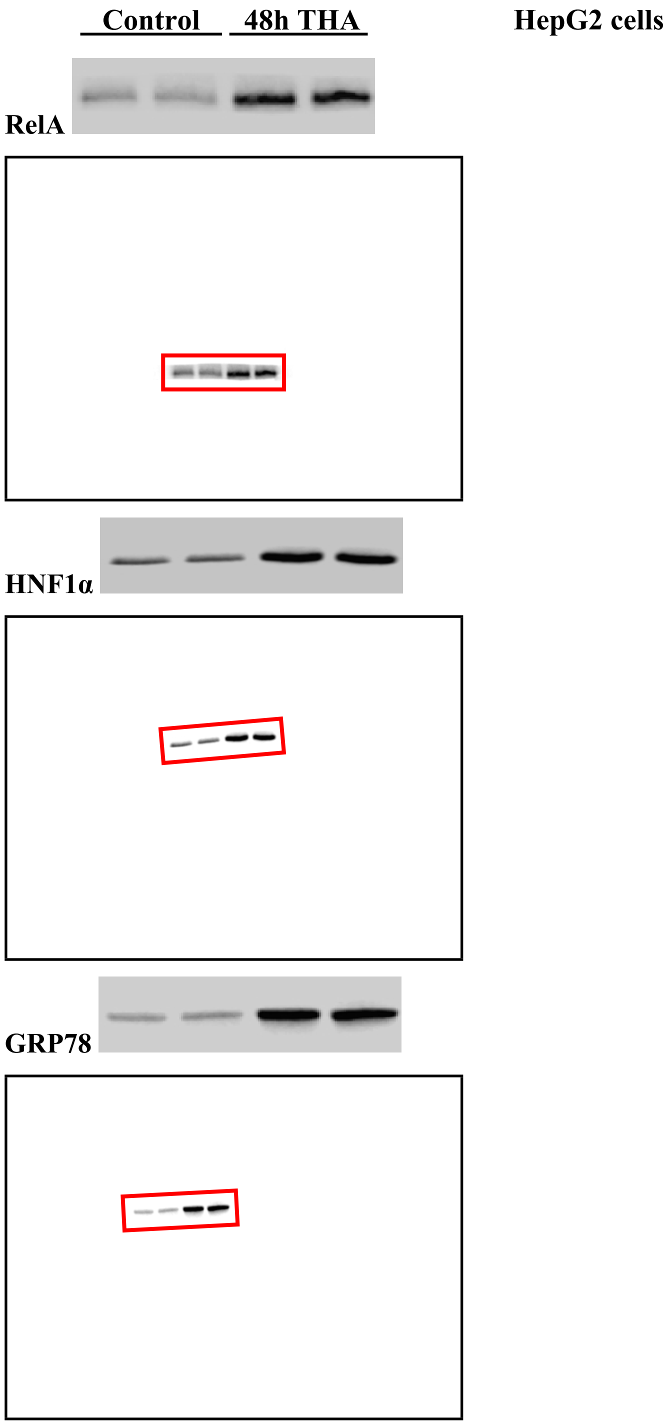

β-actin
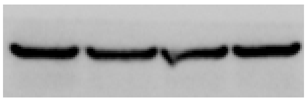
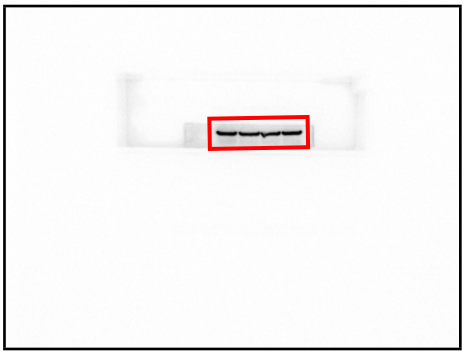

Supplementary Fig. 2J

Control TM .

Mice

RelA
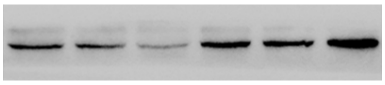
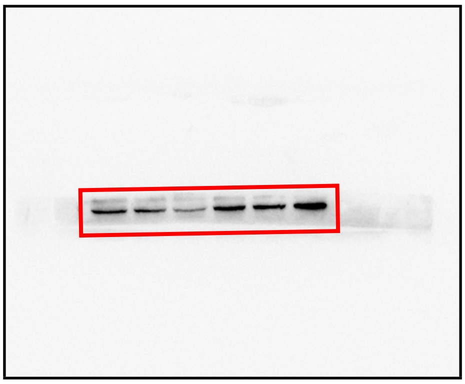

HNF1α
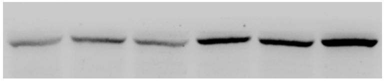
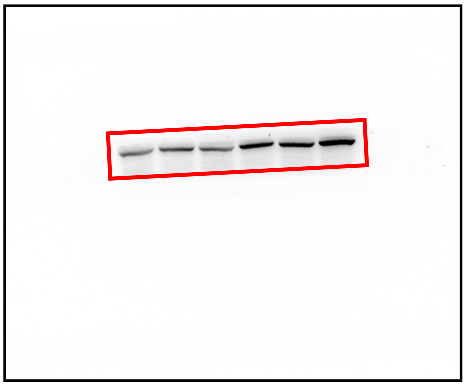

GRP78

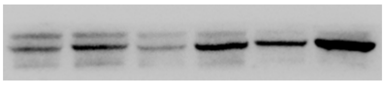

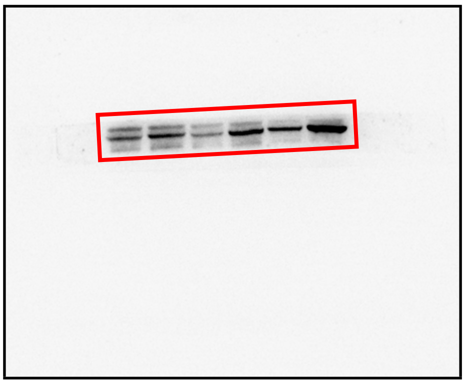

β-actin

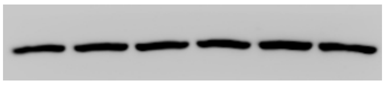

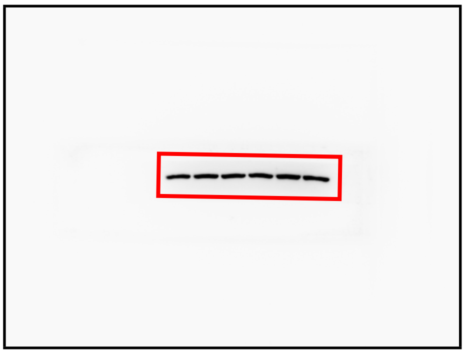

Supplementary Fig. 3A

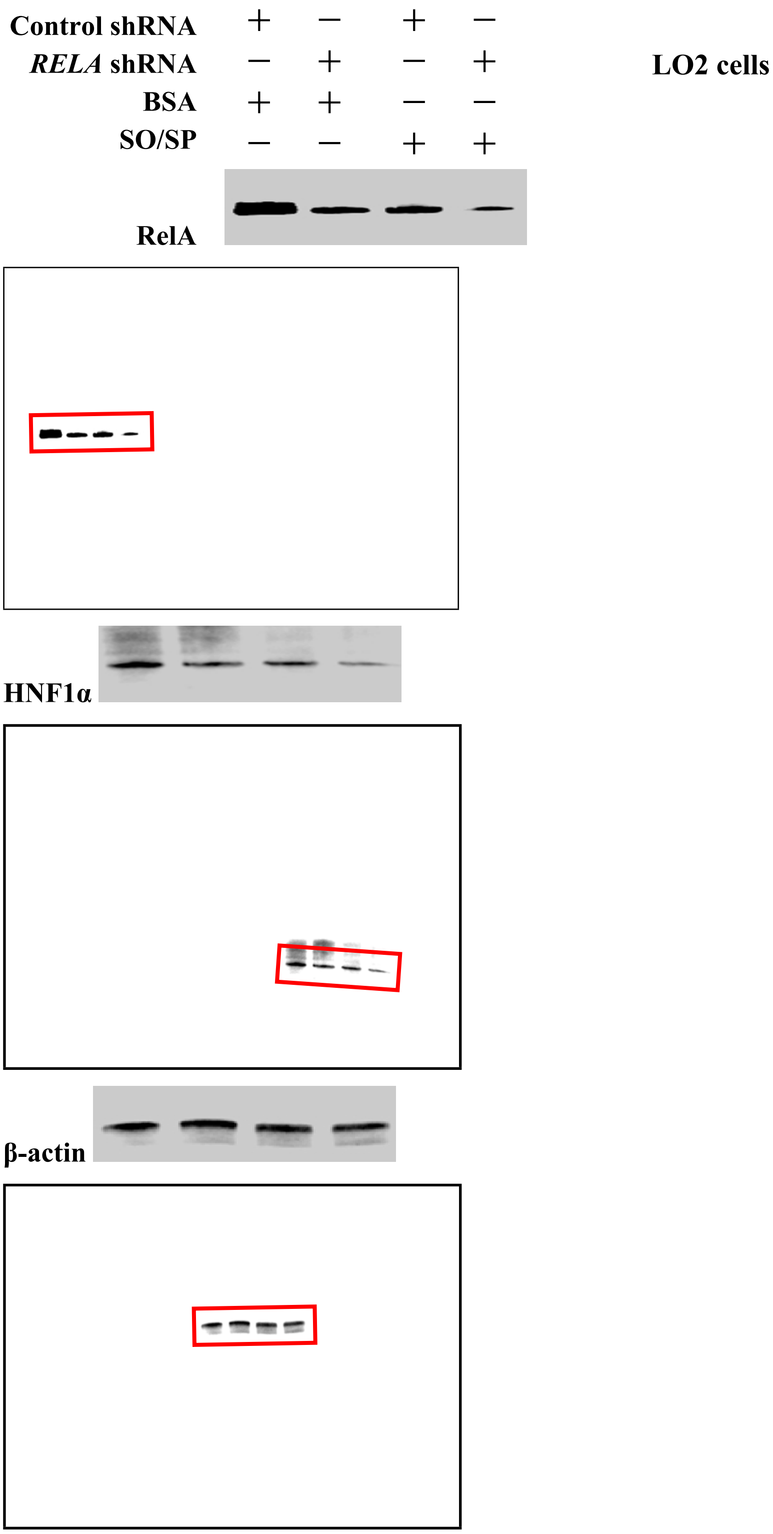

Supplementary Fig. 2B

Empty vector	+	+	−	−	LO2 cells
RELA-pcDNA	−	−	+	+	
SO/SP	+	+	+	+	

RelA

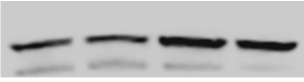

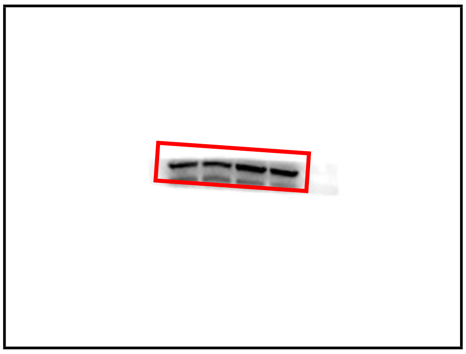

HNF1α

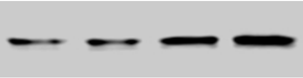

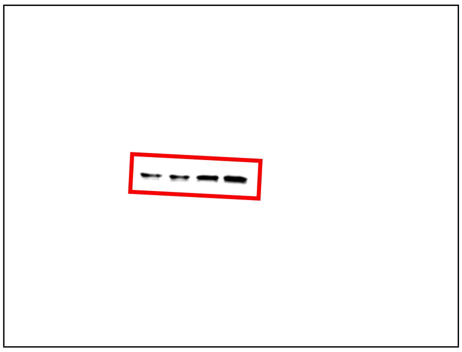

β-actin

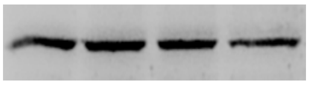

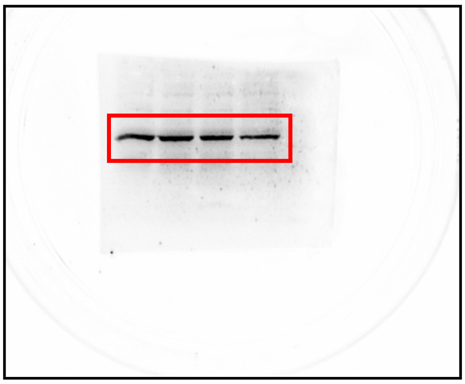

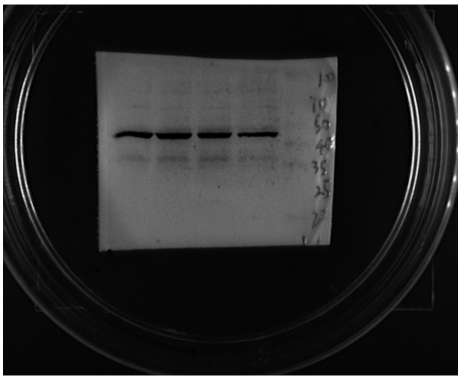

Supplementary Fig. 3C

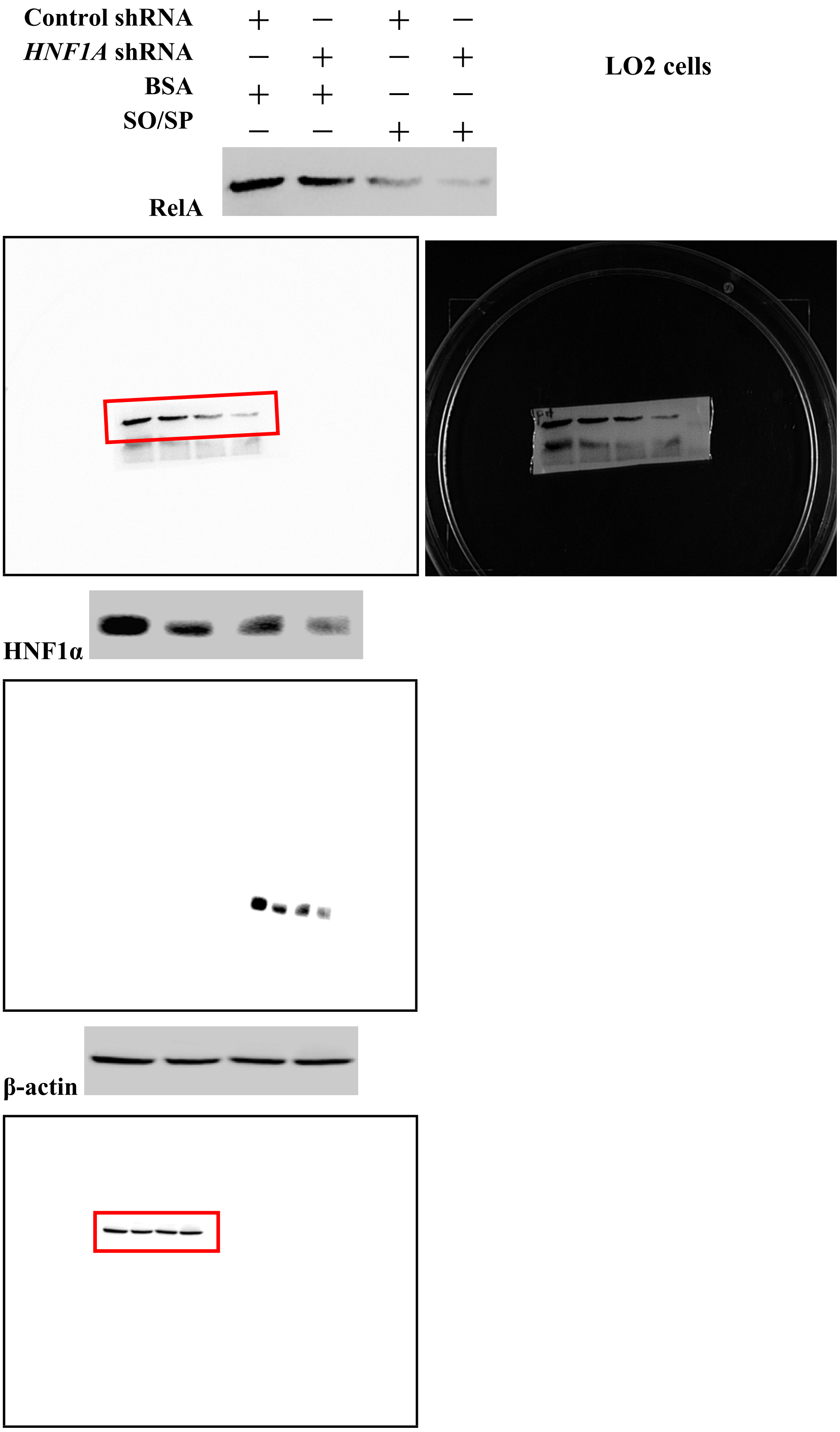

Supplementary Fig. 4E

Control shRNA	+	−	+	−	
RELA shRNA	−	+	−	+	LO2 cells
BSA	+	+	+	+	
250/125 µM SO/SP	−	−	+	+	

Cleaved caspase-3

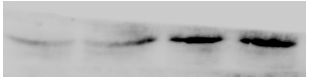

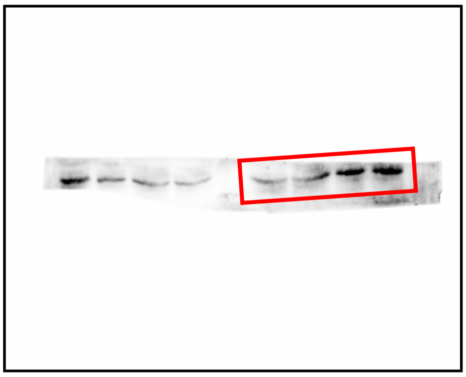

β-actin

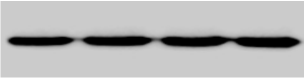

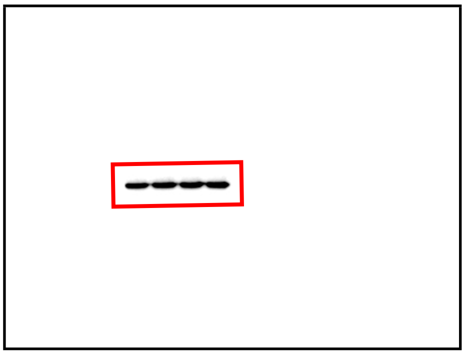

p-MLKL

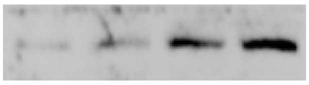

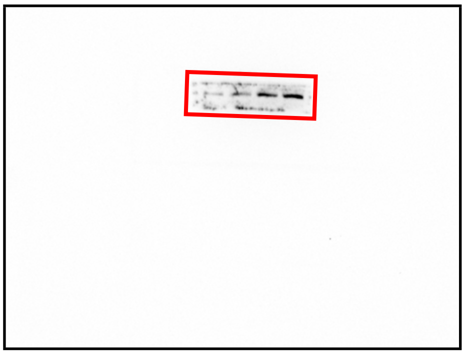

MLKL

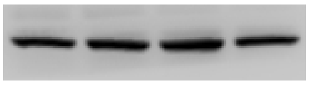

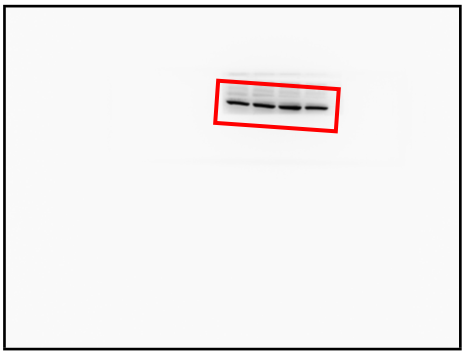

Supplementary Fig. 3J

Empty vector	+	+	−	−	LO2 cells
RELA-pcDNA	−	−	+	+	
250/125 μM SO/SP	+	+	+	+	

Cleaved caspase-3

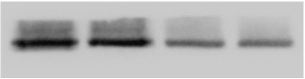

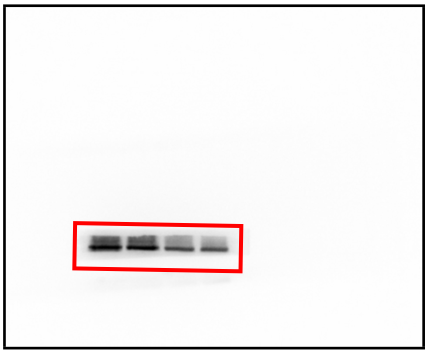

β-actin

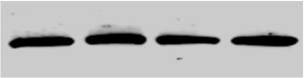

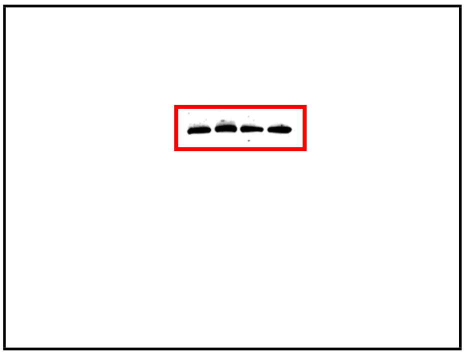

p-MLKL

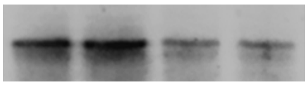

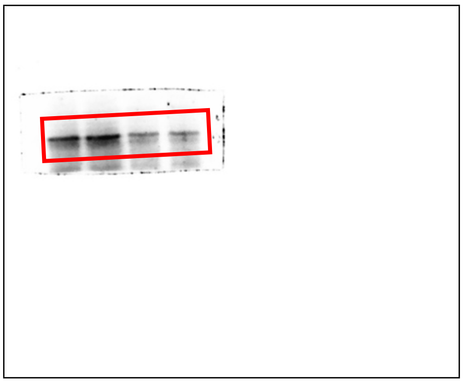

MLKL

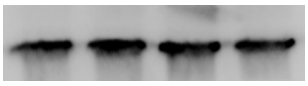

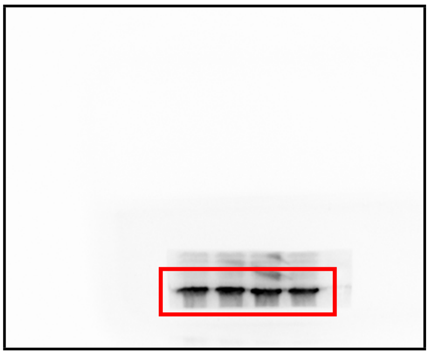

Supplementary Fig. 5E

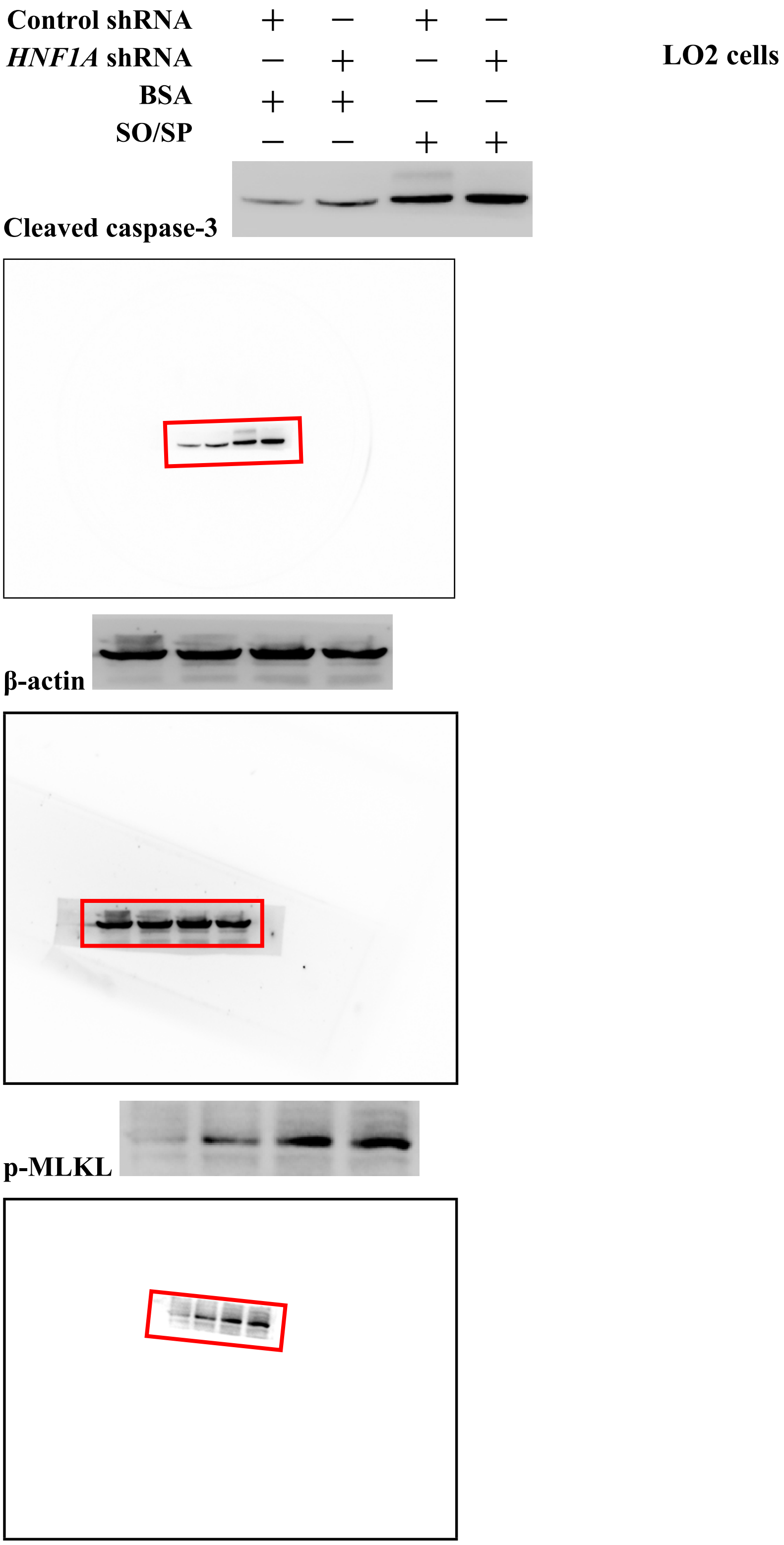

MLKL

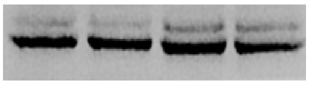

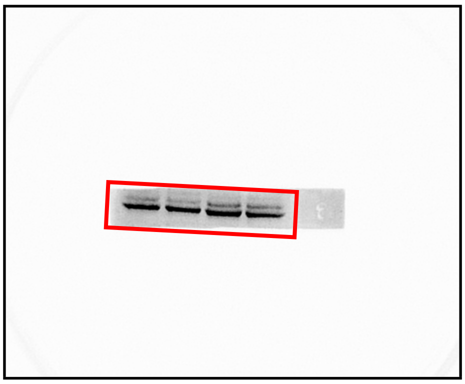

Supplementary Fig. 6L

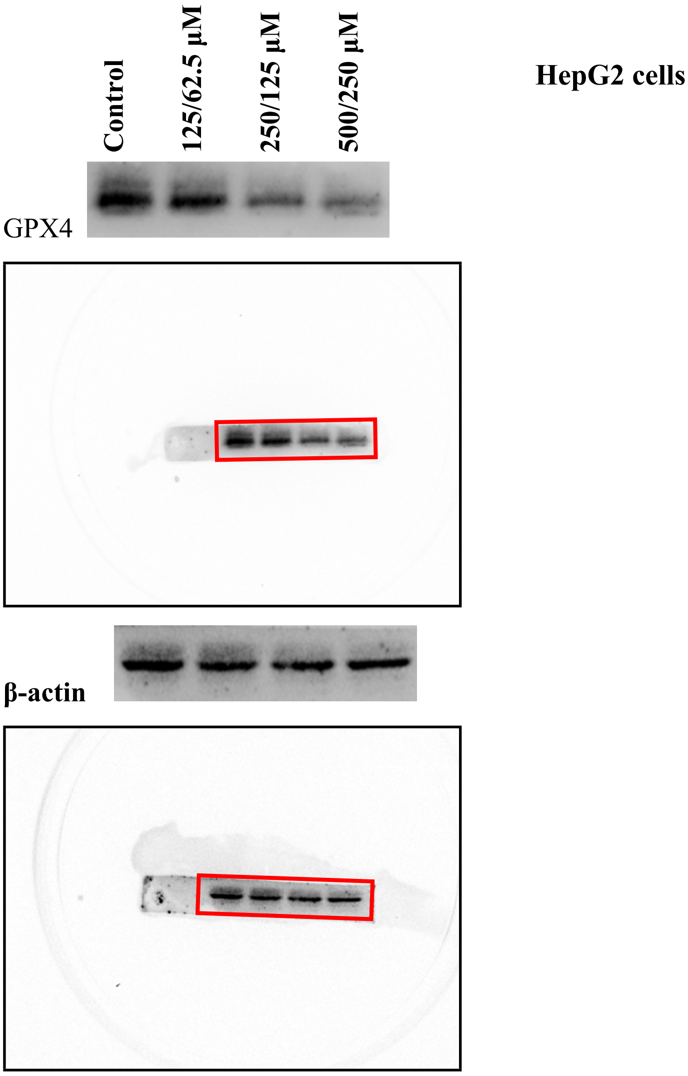

Supplementary Fig. 6M

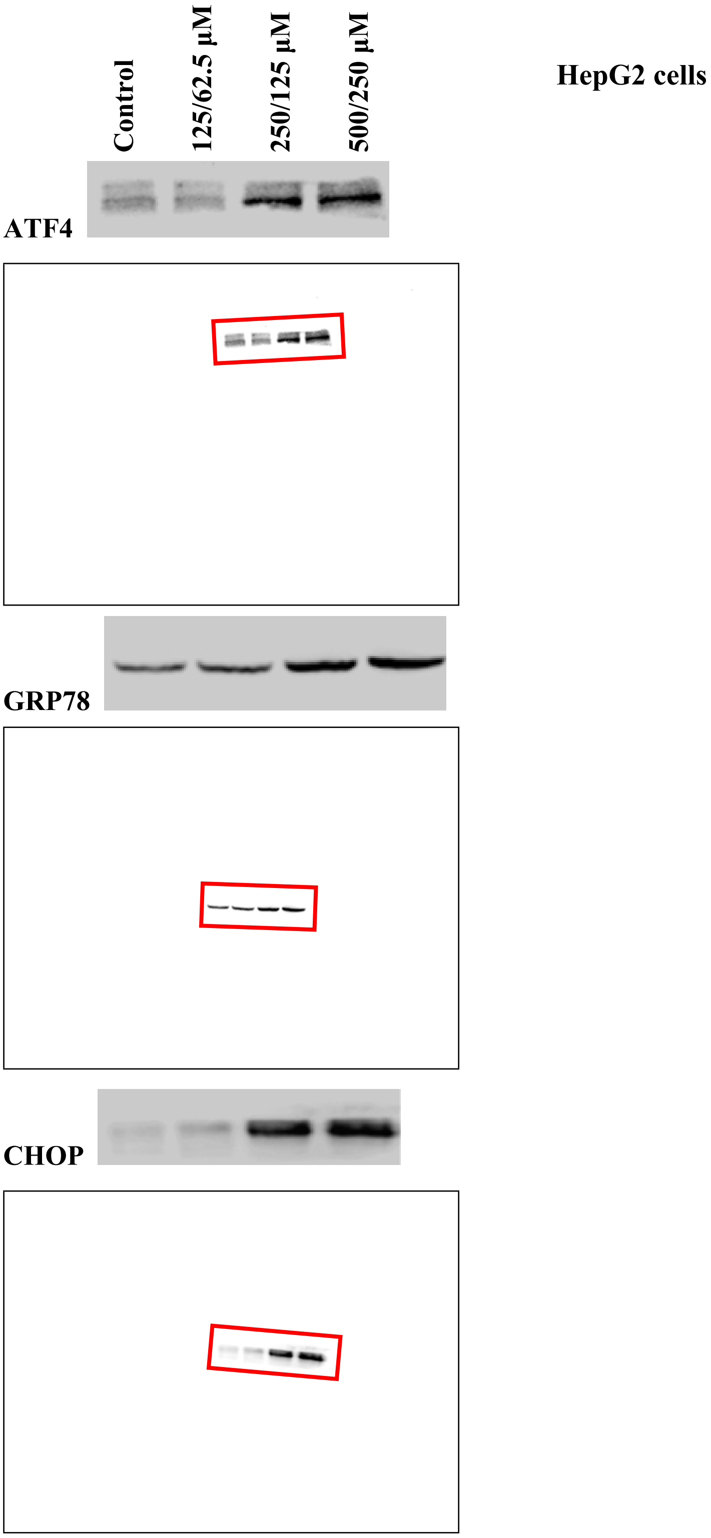

β-actin 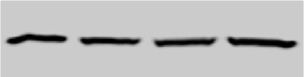

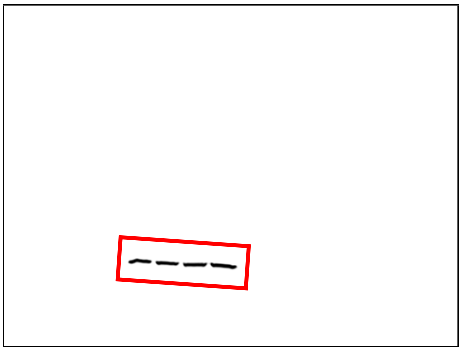

Supplementary Fig. 7D

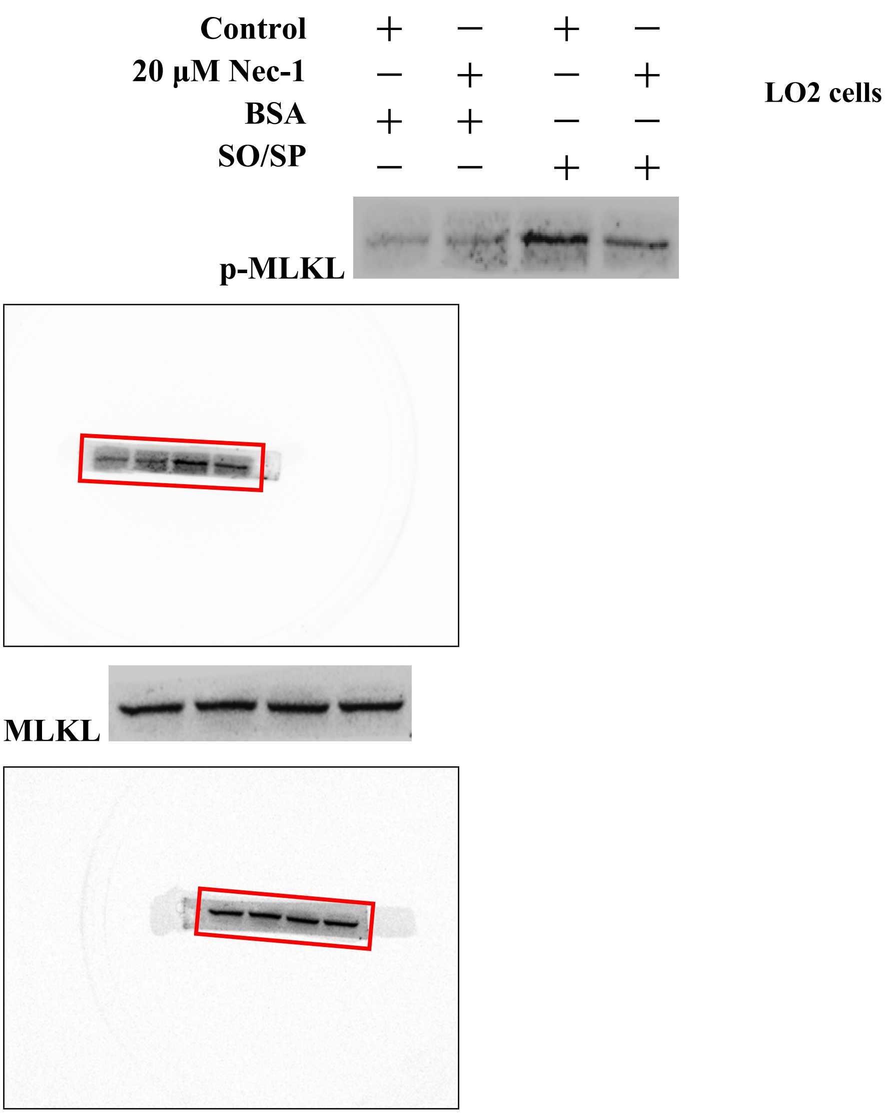

Supplementary Fig. 7I

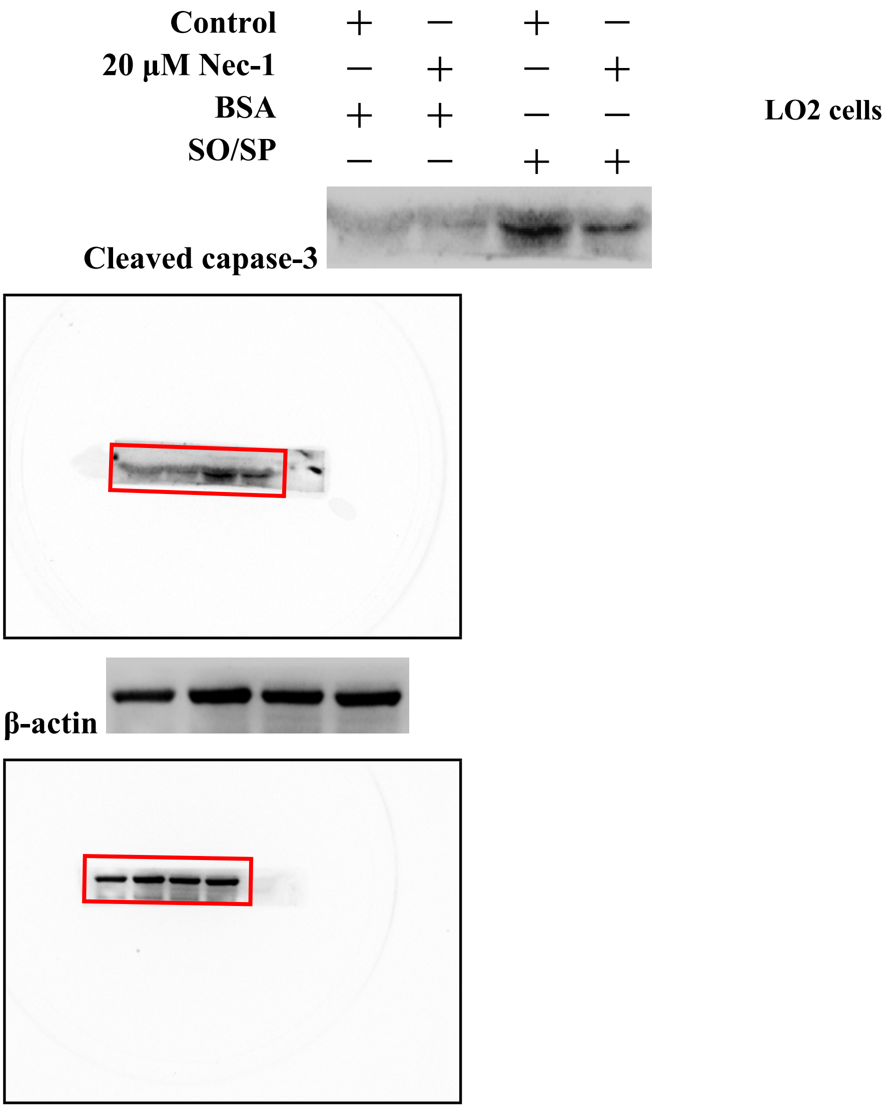

Supplementary Fig. 8M

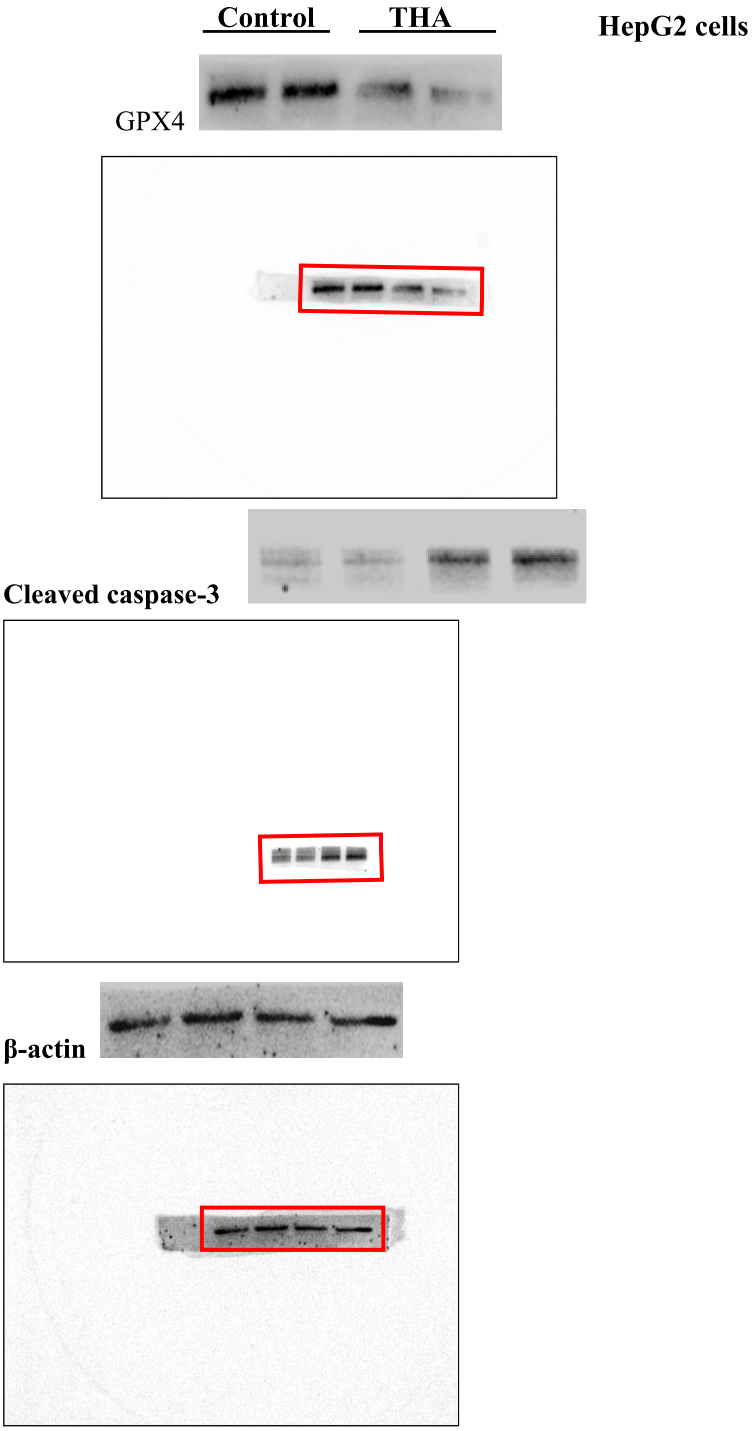

p-MLKL

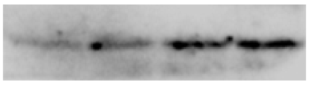

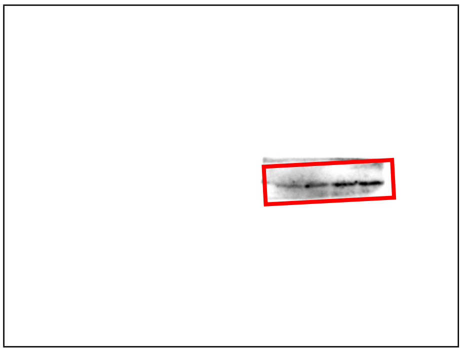

MLKL

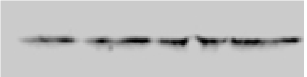

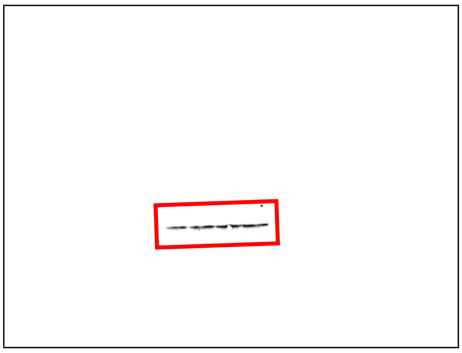

Supplementary Fig. 9A

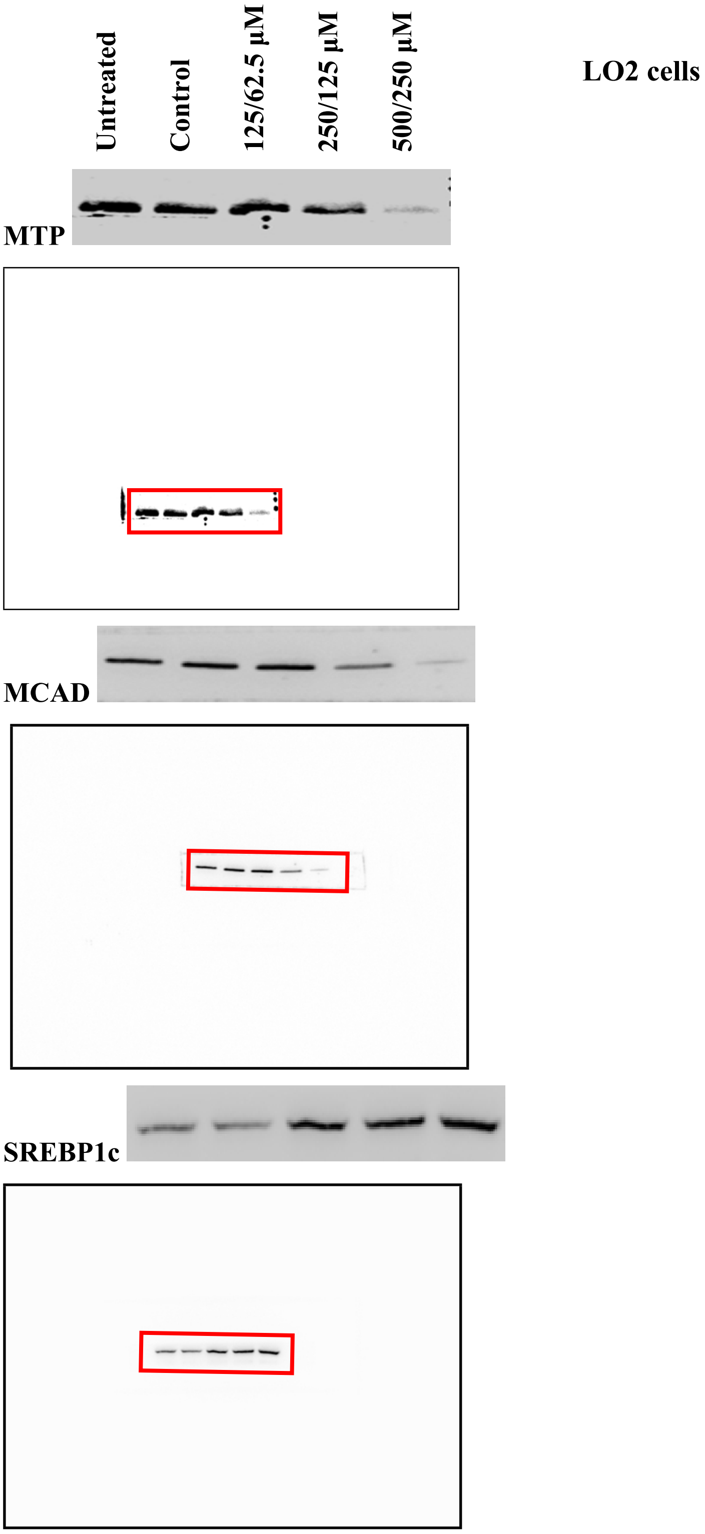

β-actin

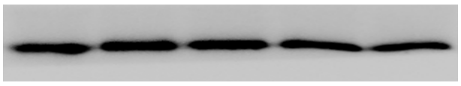

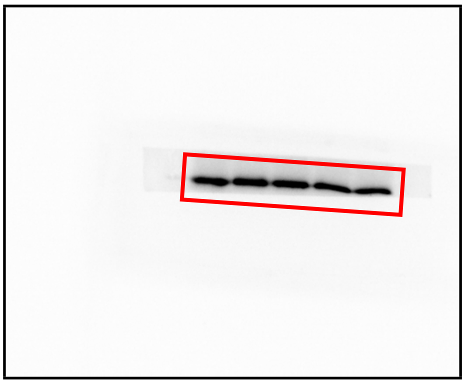

Supplementary Fig. 9B

0.5 µM THA 0h 12h 24h 48h

LO2 cells

MTP

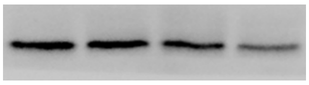

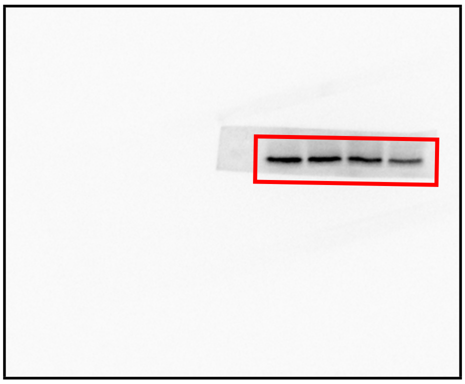

MCAD

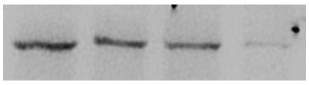

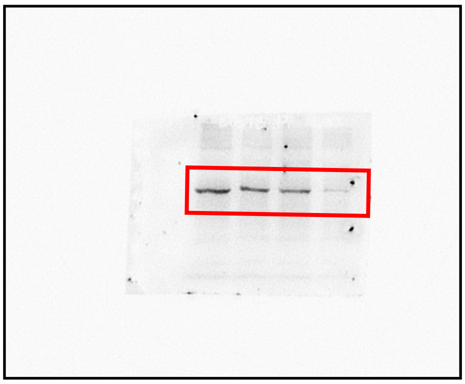

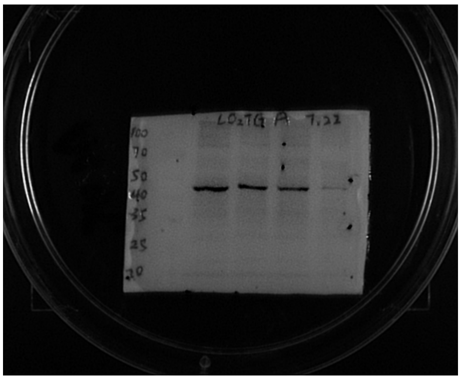

SREBP1c

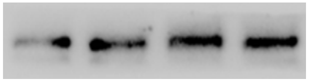

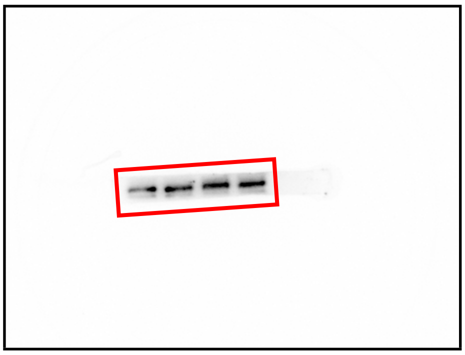

β-actin

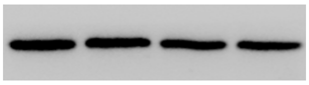

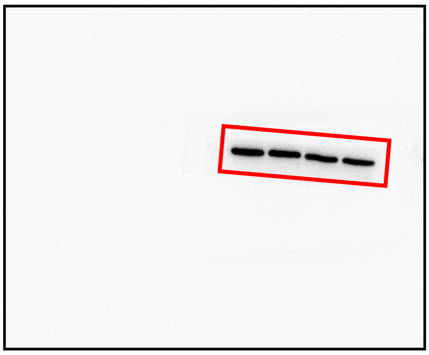

Supplementary Fig. 9C

LO2 cells

Control shRNA	+	−	+	−
RELA shRNA	−	+	−	+
BSA	+	+	+	+
250/125 μM SO/SP	−	−	+	+

MTP

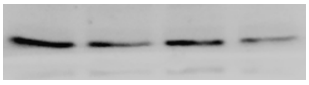

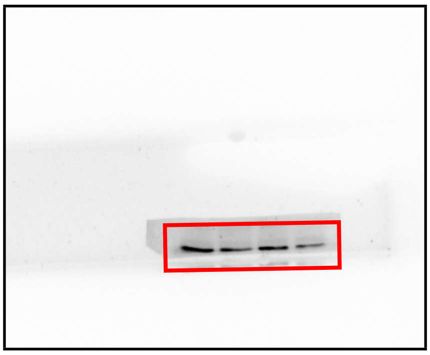

MCAD

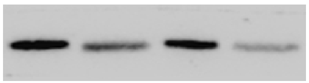

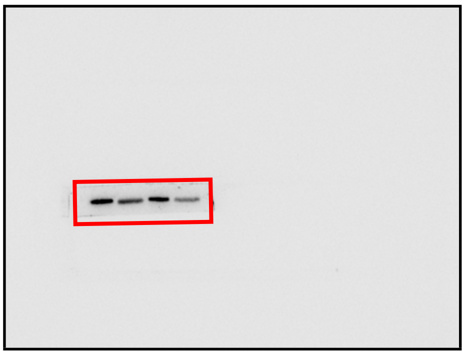

SREBP1c

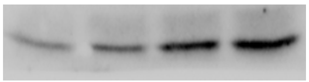

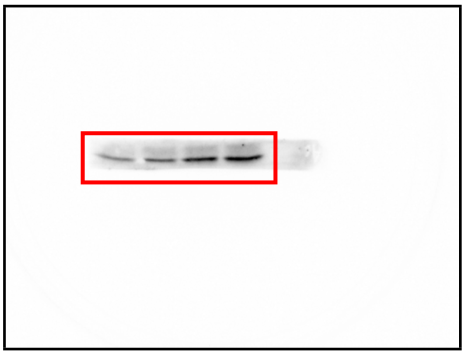

β-actin

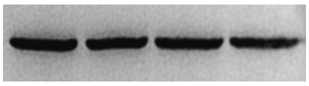

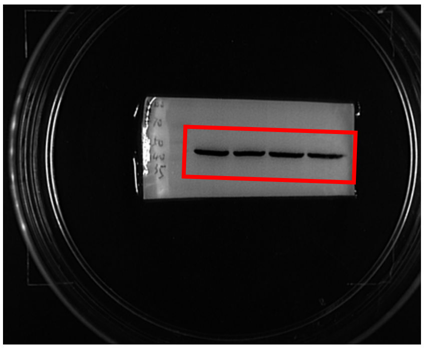

Supplementary Fig. 9D

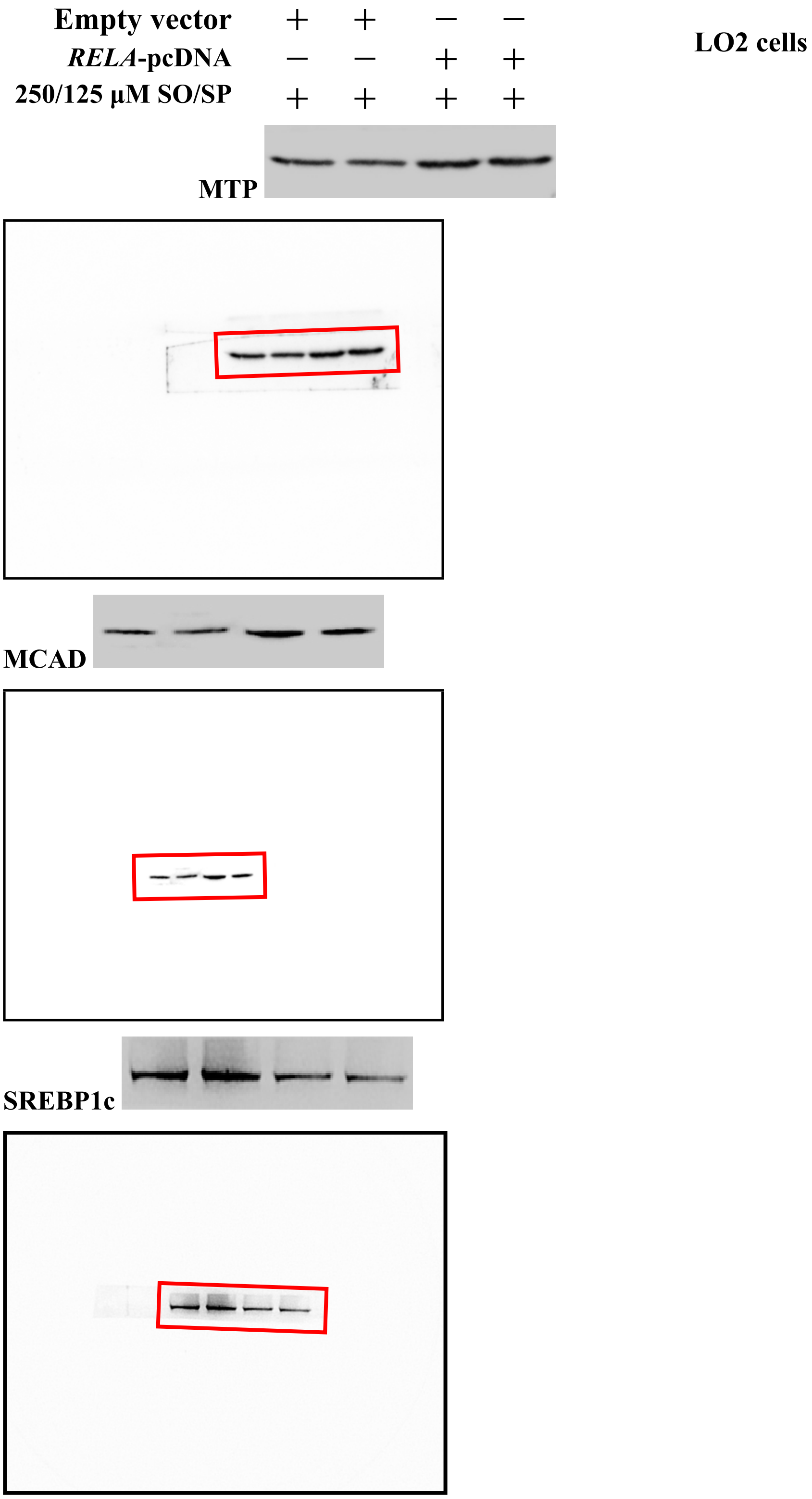

β-actin

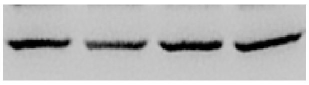

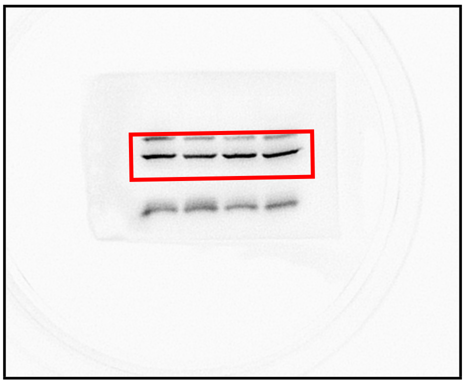

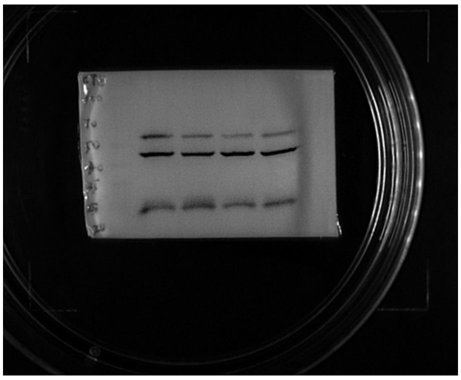

Supplementary Fig. 9I

Control TM Mice

MTP

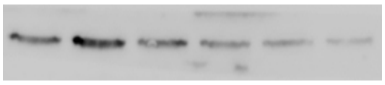

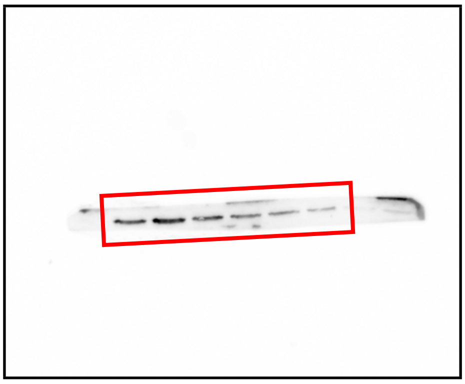

MCAD

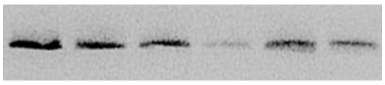

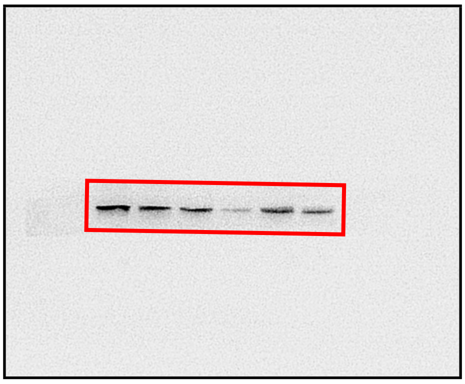

SREBP1c

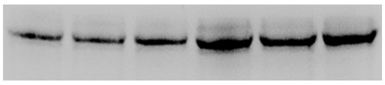

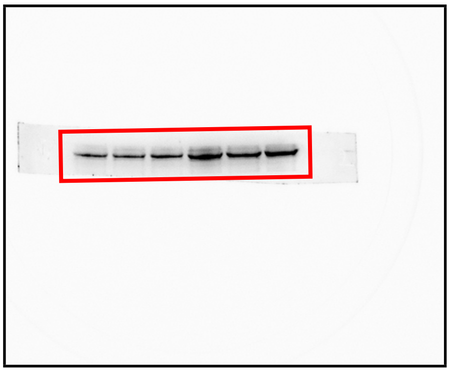

β-actin

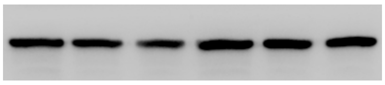

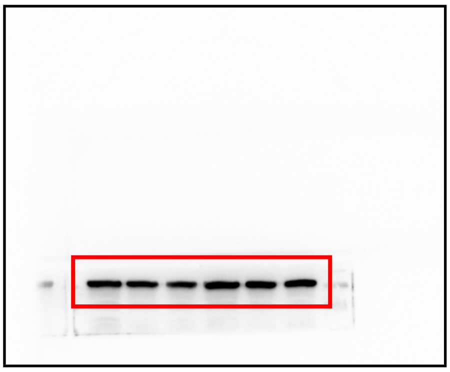

Supplementary Fig. 9J

Control shRNA	+	−	+	−	
Hnf1a shRNA	−	+	−	+	Mice
HFD	−	−	+	+	

MTP

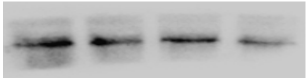

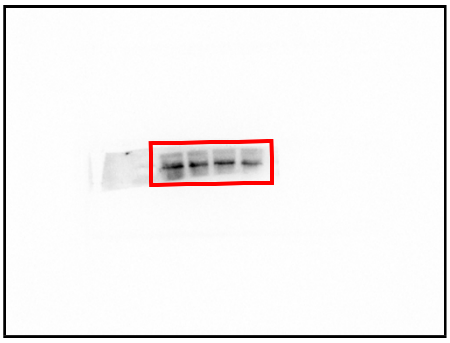

MCAD 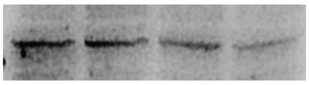

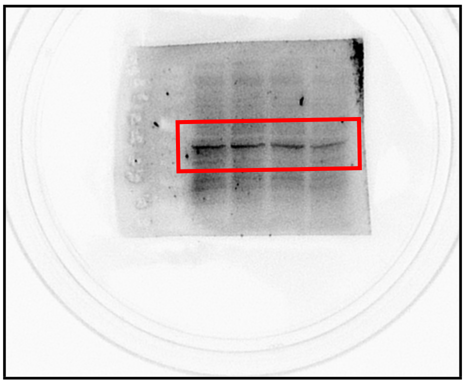 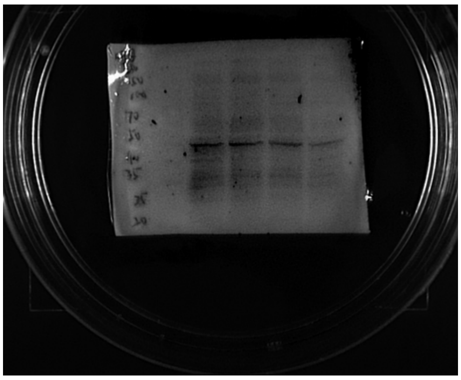

SREBP1c 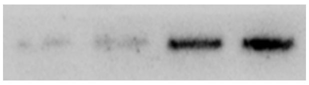

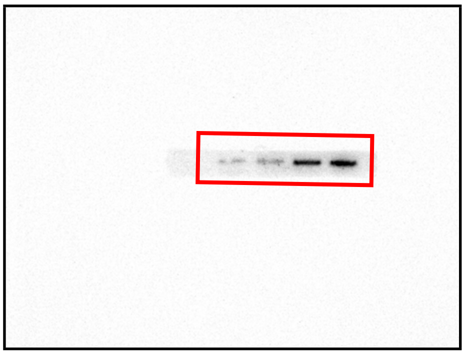

β-actin 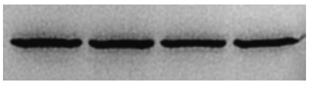

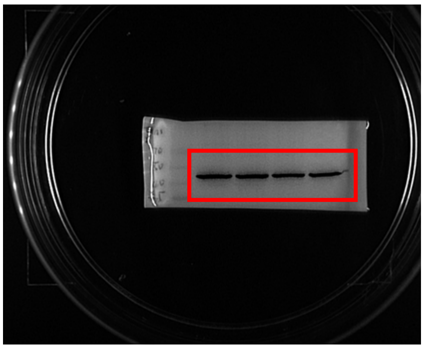

Fig. 9D

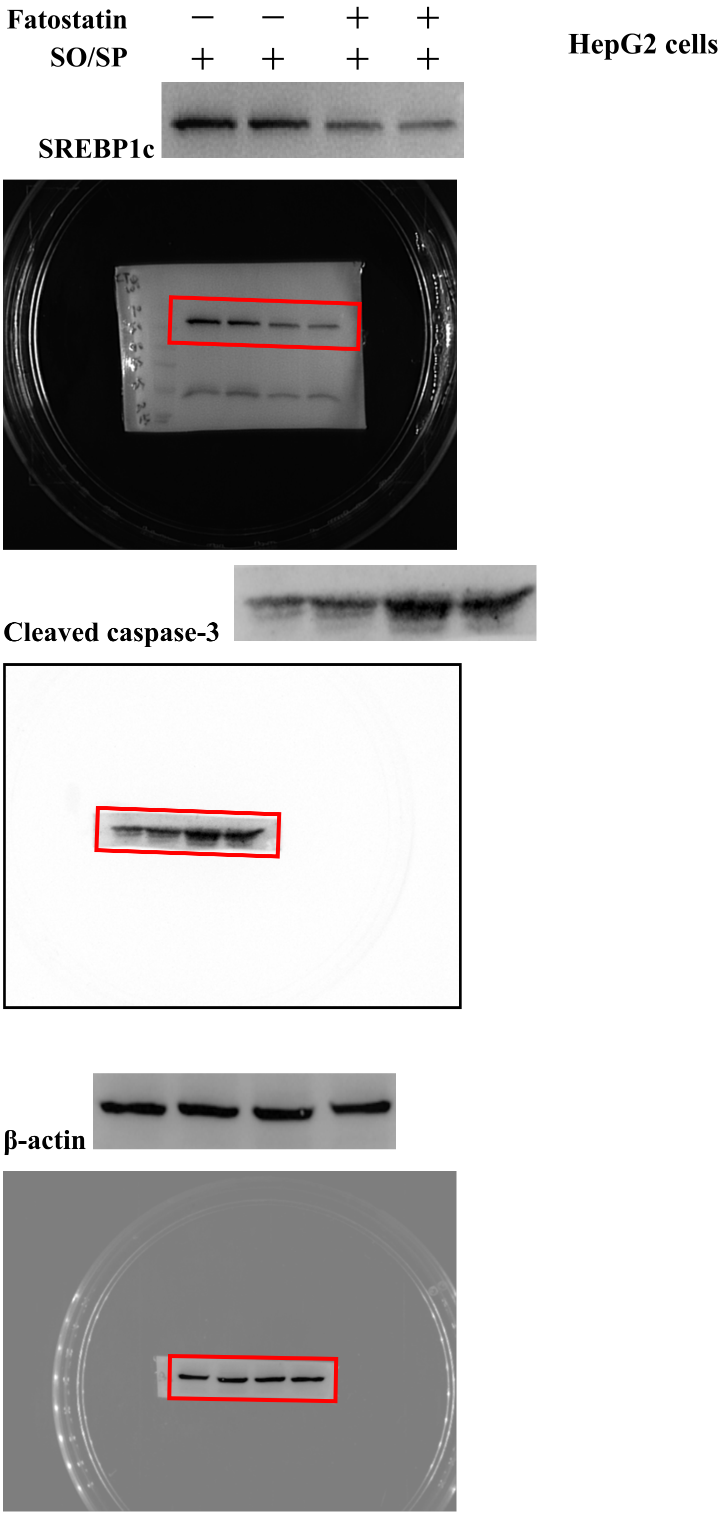
Fatostatin − − + +
SO/SP + + + +
HepG2 cells
SREBP1c
Cleaved caspase-3
β-actin

p-MLKL 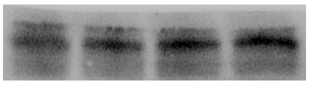

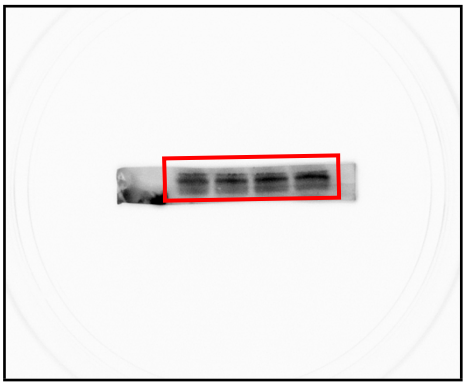

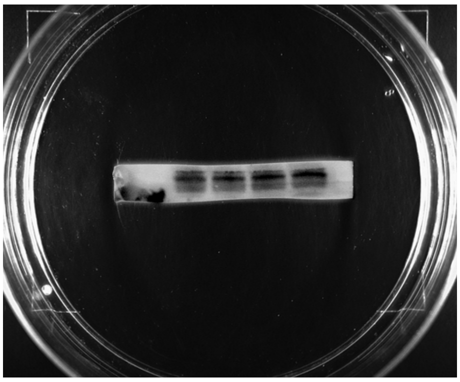

MLKL 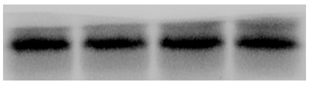

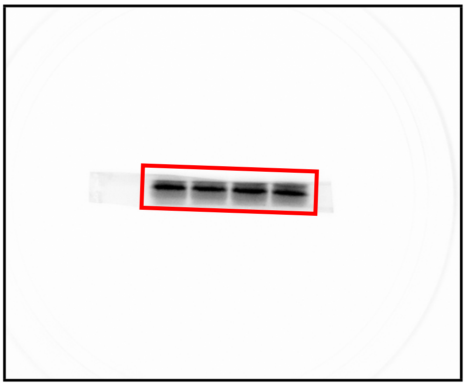

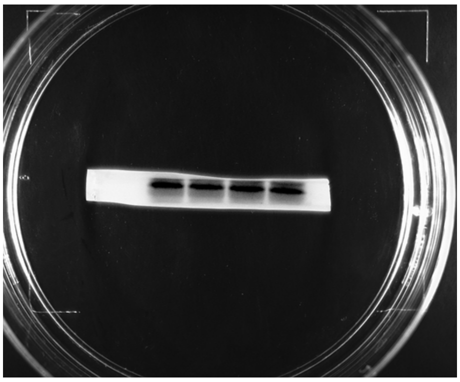

Supplementary Fig. 11H

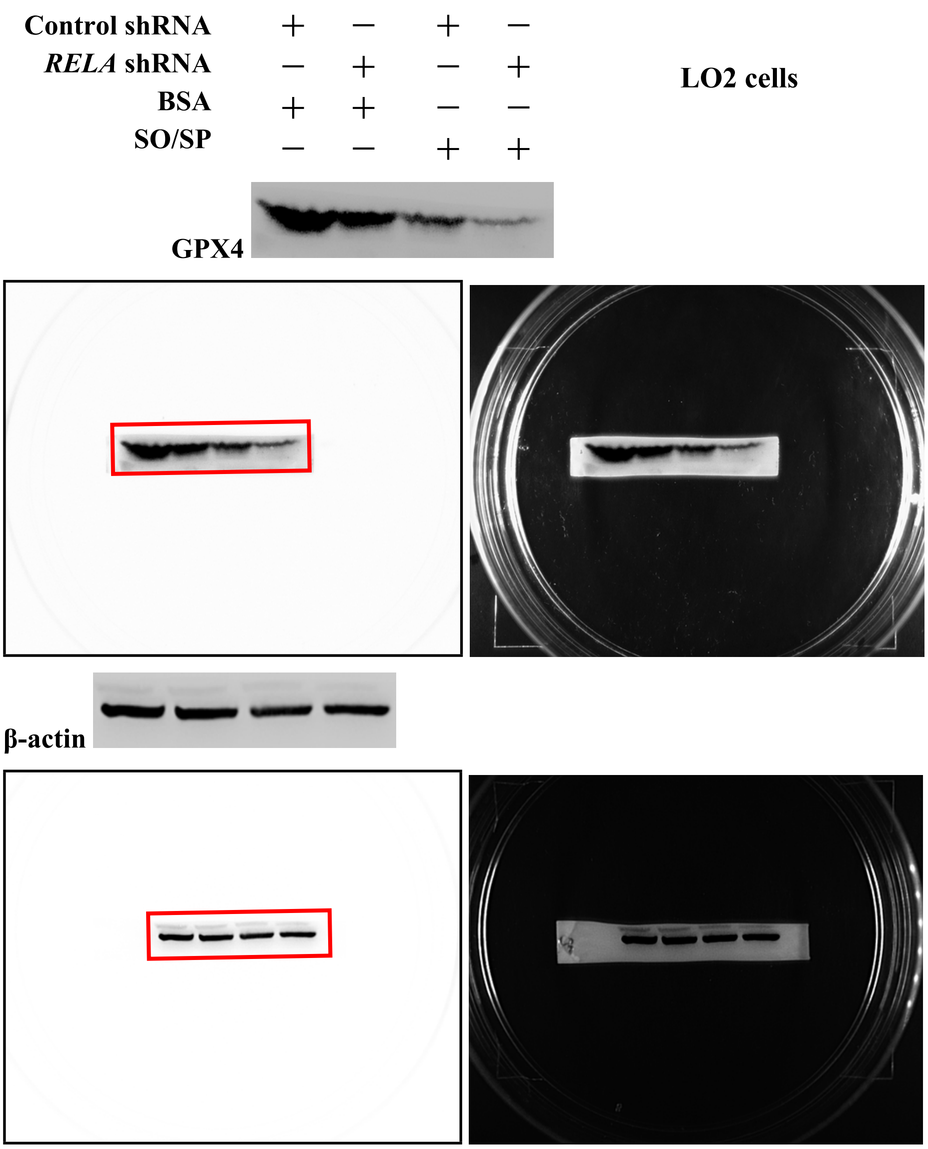

Supplementary Fig. 11I

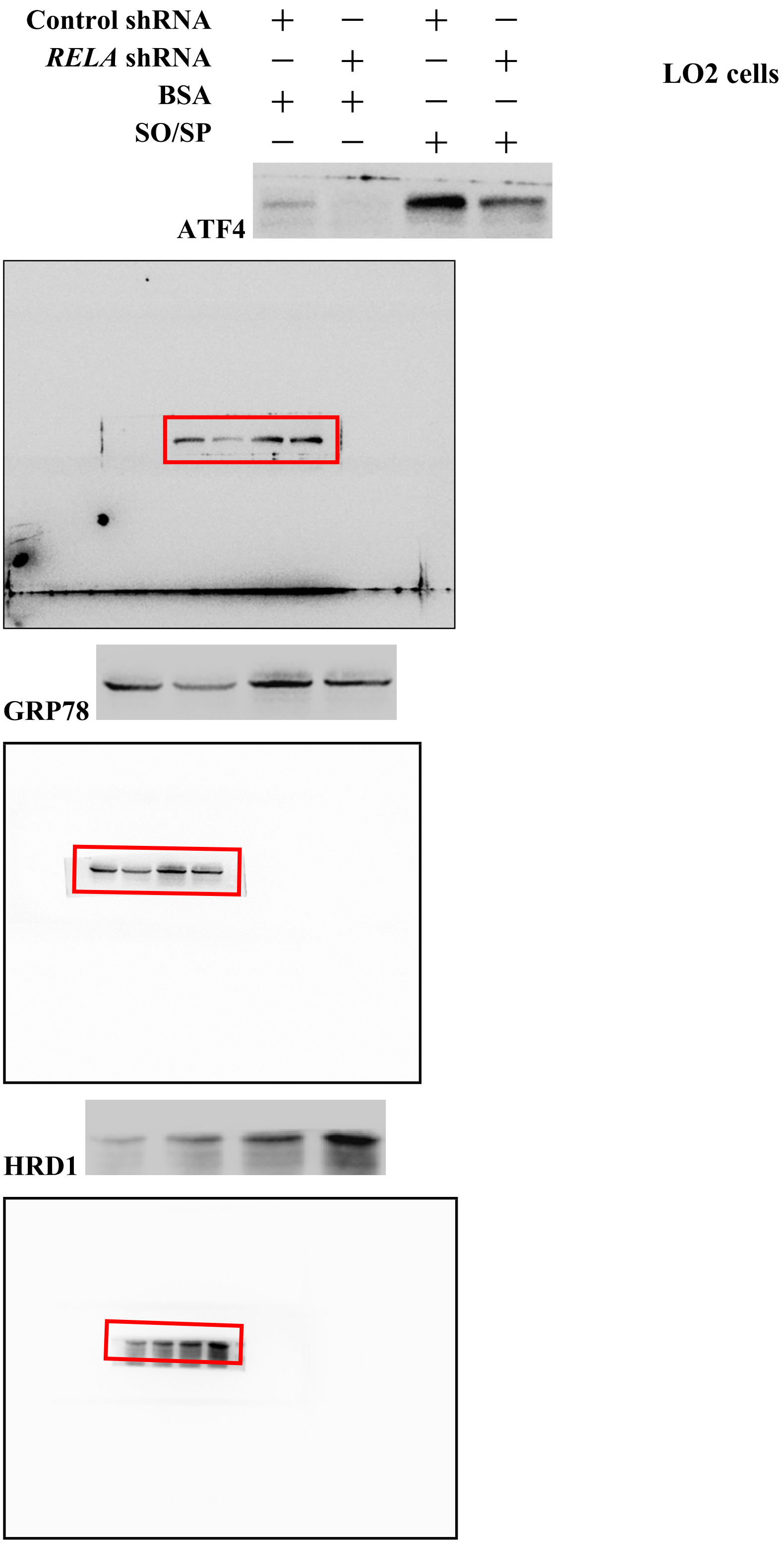

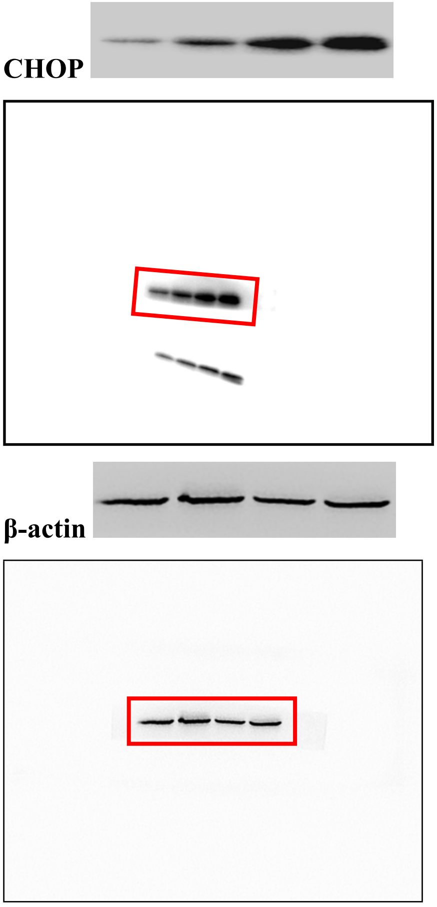

Supplementary Fig. 11J

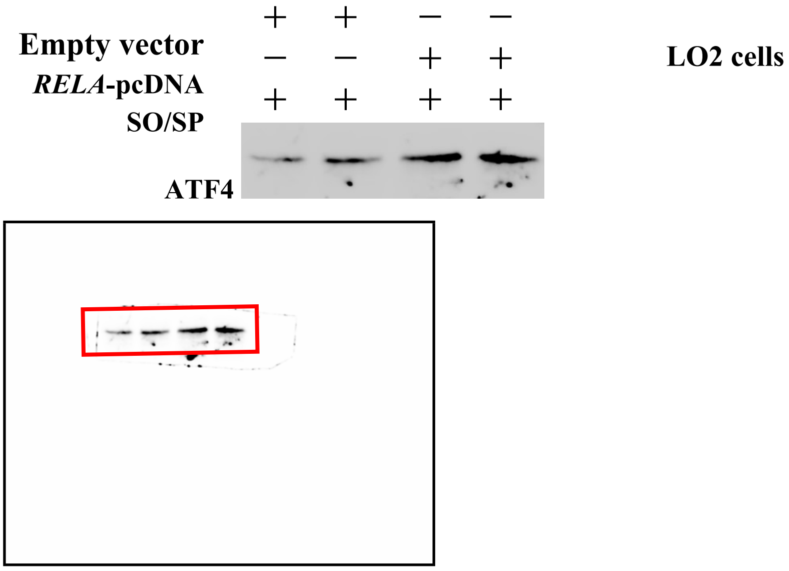

GRP78 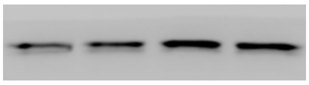

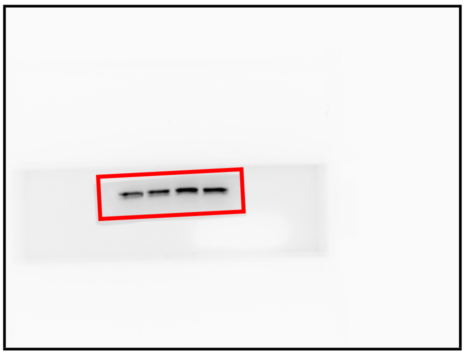

HRD1 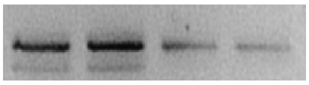

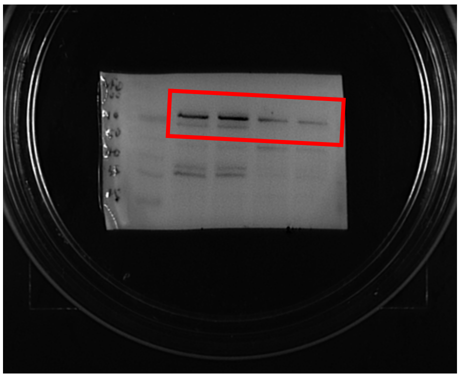

CHOP 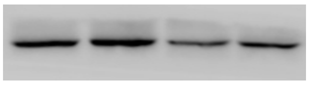

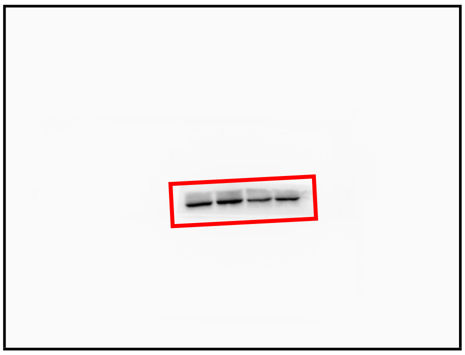

β-actin 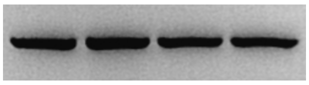

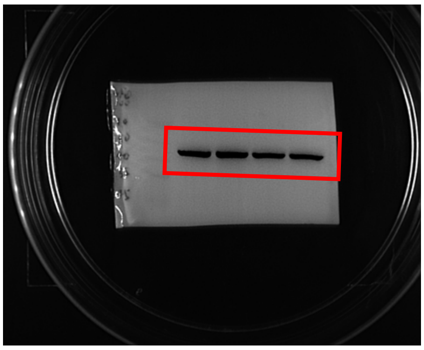

Supplementary Fig. 11K

Vehicle	+	+	–	–
HNF1A-KO	–	–	+	+
SO/SP	+	+	+	+

HepG2 cells

ATF4 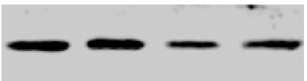

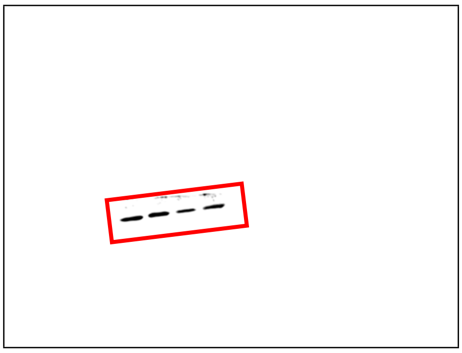

GRP78 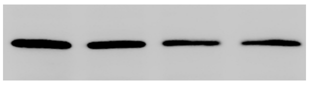

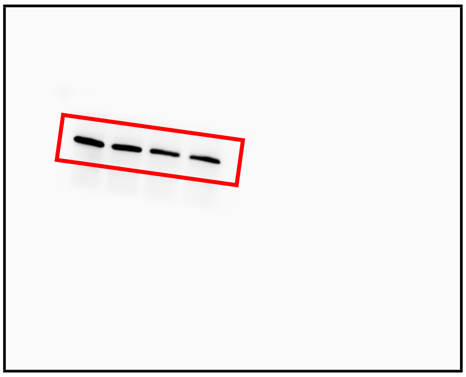

HRD1

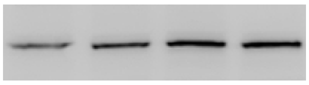

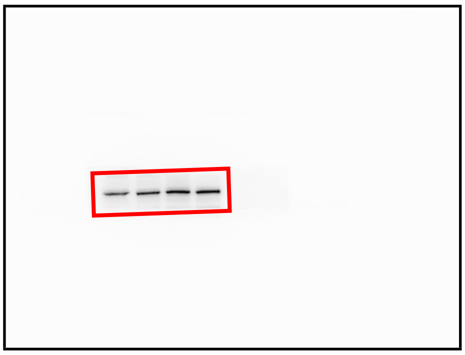

CHOP

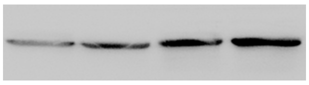

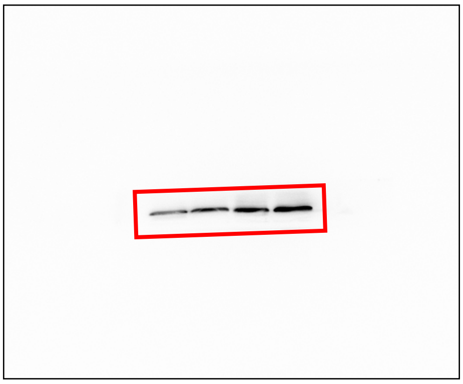

β-actin

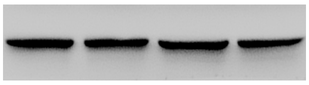

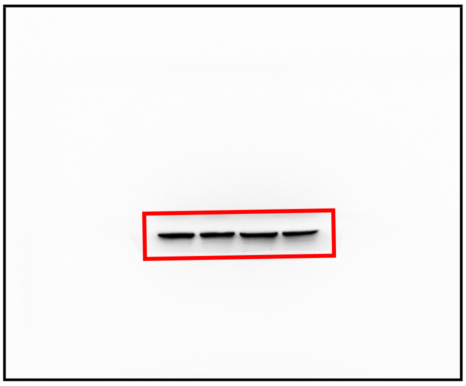

Supplementary Fig. 11L

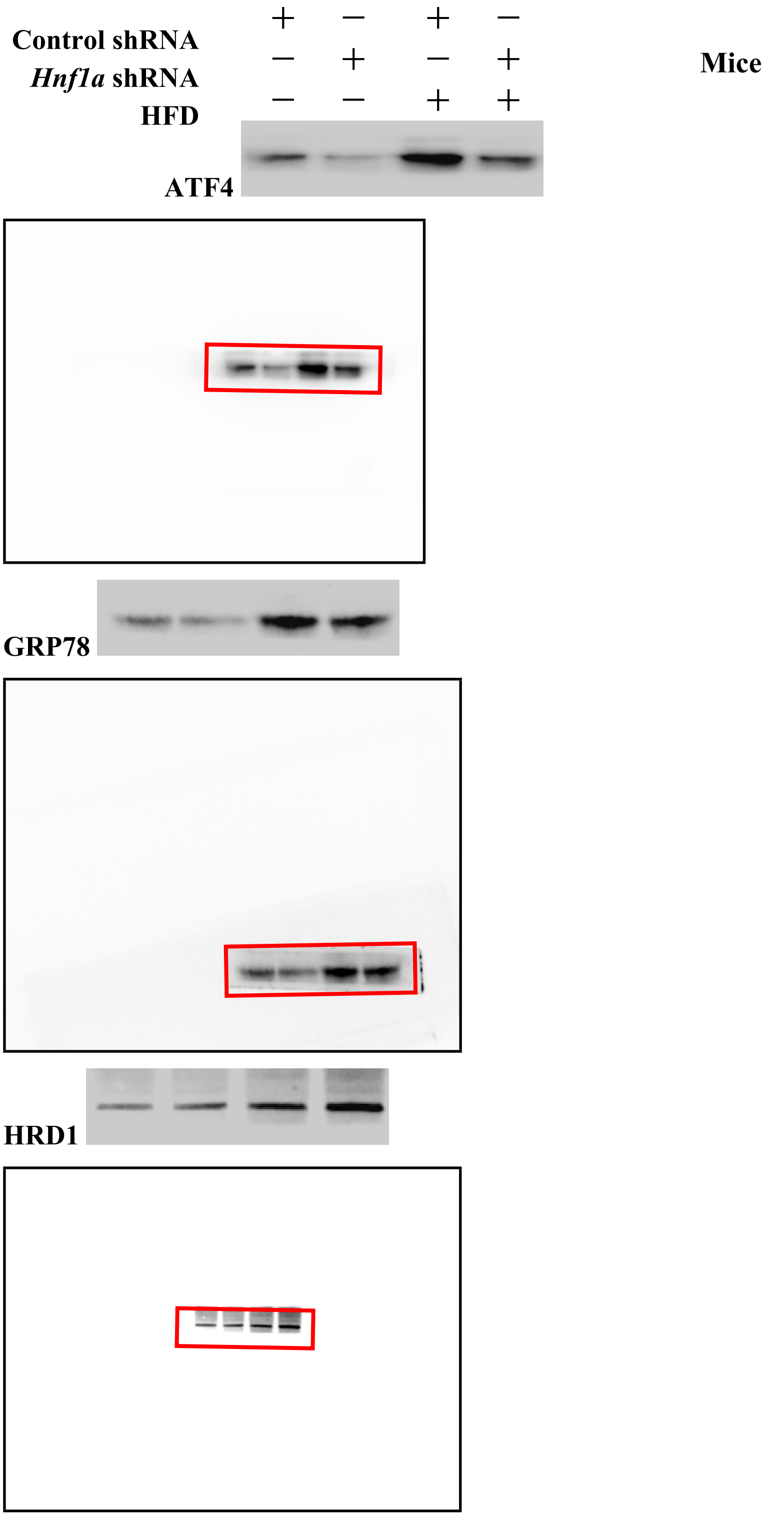

CHOP

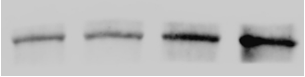

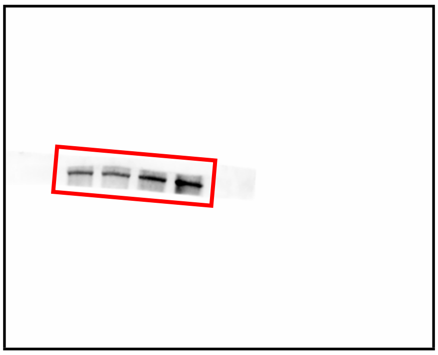

β-actin

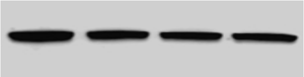

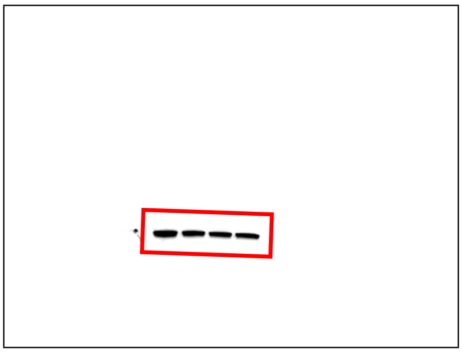

Supplement: Supplementary file 1 — Supplementary Materials [file 41420_2025_2312_MOESM1_ESM.pdf]
